# Prediction of Protein Complexes in *Trypanosoma brucei* by Protein Correlation Profiling Mass Spectrometry and Machine Learning*[Fn FN2]

**DOI:** 10.1074/mcp.O117.068122

**Published:** 2017-10-17

**Authors:** Thomas W. M. Crozier, Michele Tinti, Mark Larance, Angus I. Lamond, Michael A. J. Ferguson

**Affiliations:** From the ‡Division of Biological Chemistry and Drug Discovery and; §Centre for Gene Regulation and Expression, School of Life Sciences, University of Dundee, Dundee DD2 1NW, UK

## Abstract

A disproportionate number of predicted proteins from the genome sequence of the protozoan parasite *Trypanosoma brucei*, an important human and animal pathogen, are hypothetical proteins of unknown function. This paper describes a protein correlation profiling mass spectrometry approach, using two size exclusion and one ion exchange chromatography systems, to derive sets of predicted protein complexes in this organism by hierarchical clustering and machine learning methods. These hypothesis-generating proteomic data are provided in an open access online data visualization environment (http://134.36.66.166:8083/complex_explorer). The data can be searched conveniently via a user friendly, custom graphical interface. We provide examples of both potential new subunits of known protein complexes and of novel trypanosome complexes of suggested function, contributing to improving the functional annotation of the trypanosome proteome. Data are available via ProteomeXchange with identifier PXD005968.

Trypanosoma brucei is a unicellular trypanosomatid protozoan parasite and the etiological agent of sleeping sickness in sub-Saharan Africa, estimated to cause ∼20,000 cases per year (1). Current treatments are expensive, difficult to administer and toxic. In 2005, the genomic sequence of *T.brucei* was reported, with ∼9,100 genes identified, thereby providing a valuable resource for the trypansomatid research community (2). However, many of the identified genes encode predicted proteins that lack classifiable homology to known proteins in other organisms, hampering their functional classification. It has been previously estimated that ∼4900 *T. brucei* genes lack reliable orthologs in other organisms and are annotated as “hypothetical” (3, 4). This lack of functional genome annotation hinders our understanding of trypanosome biology and associated therapeutic possibilities.

Many intracellular biological processes are dependent on the stable physical association between two or more proteins (5). Indeed, many proteins require to be part of a complex to carry out their function, including the subunits of many well characterized complexes, such as the proteasome, ribosome and spliceosome. The global characterization of model organism interactomes has led to greater understanding of proteome organization and improved the functional annotation of uncharacterized proteins via “guilt by association” (6–10).

Protein correlation profiling mass spectrometry (PCP-MS)^1^ has been used to identify and predict the subunit composition of protein complexes through identifying proteins cofractionating by liquid chromatography and/or sedimentation methods (11–13). Recently, PCP-MS has been used to analyze soluble protein complexes through the fractionation of whole cell lysates by size-exclusion chromatography (SEC) (14–17) and ion-exchange chromatography (IEX) (18), identifying hundreds of protein complexes in single experiments. The combination of orthogonal chromatographic techniques has been shown to better resolve individual protein complexes, which may coelute by chance under the conditions of a single fractionation method (18).

During the course of the investigation described in this paper, Gazestani and colleagues reported using glycerol gradient centrifugation and ion-exchange chromatography to identify protein complexes in procyclic form *T.brucei* mitochondrial and cytoplasmic lysates, in single biological replicates (19). Taking a similar approach, we have used ultra-high performance liquid chromatography (uHPLC), to fractionate whole cell lysates from procyclic form *T. brucei*, using SEC and strong anion exchange chromatography (SAX), in up to four biological replicates. We identified and quantified elution profiles for 6004 protein groups. Computational analysis allowed us to predict 234 protein complexes. This data set includes many well-characterized complexes in *T. brucei*, supports the existence of other complexes that were predicted based on homology to known complexes from other organisms and additionally provides evidence for the existence of novel, previously uncharacterized complexes. By incorporating these data into a freely accessible, online database, we provide a useful resource for both trypanosome biologists and for all groups studying protein-protein interactions and complexes in other organisms.

## EXPERIMENTAL PROCEDURES

### 

#### 

##### SDM-79 Media Preparation

Powdered SDM-79 media was hydrated with 5 L of Milli-Q water, and supplemented with hemein to 7.5 mg/L and 2 g/L of sodium bicarbonate. The pH was adjusted to 7.3 with NaOH, and sterile filtered using Stericups 500. Under sterile conditions, heat inactivated and nondialyzed fetal bovine serum (PAA) was added to 15% (v/v) and Glutamax I to 2 mm, final concentrations, respectively. The antibiotics, G418 and hygromycin, were used at final concentrations of 15 μg/ml and 50 μg/ml respectively.

##### Cell Culture

Procyclic trypanosomes (clone 29.13.6) were cultured in SDM-79 media at 28 °C, without CO_2_, in fully capped culture flasks.

##### Size-exclusion Chromatography (SEC)

Procyclic trypanosomes (3 × 10^9^ cells/replicate) were washed three times in 50 ml of phosphate-buffered saline (PBS) and lysed using a Bioruptor Pico (Diagenode, Liege, Belgium) water bath sonicator for 10 cycles of 30 s on/off, in 0.75 ml of PBS containing 0.1 μm 1–5-chloro-3-tosylamido-7-amino-2-heptone (TLCK), 1 mm phenyl-methyl sulfonyl fluoride (PMSF), 1 μg/ml leupeptin, 1 μg/ml pepstatin and 5 mm ethylenediametetraacetic acid (EDTA). Lysates were centrifuged at 17,000 × *g* for 10 min and the supernatant filtered through a 0.45 μm filter unit, all at 4 °C. Bradford assays were performed on the filtrates for protein quantitation.

Filtered lysates (∼1.2 mg in 200 μl, ∼8 × 10^8^ cell equivalents) were injected onto either a BioBasic SEC 300, or a BioBasic SEC 1000 column (5 μm, 300 × 7.8 mm with either 30 nm, or 100 nm pore size, respectively), using a Dionex Ultimate 3000 uHPLC system and collected in 48 fractions of 120 μl. Columns were equilibrated with PBS and eluted at a flow rate of 0.3 ml/min at 4 °C.

Each fraction was made up to 0.1 m Tris-HCl (pH 8.0), 1 m urea and 5 mm dithiothreitol and incubated for 2 h at 37 °C, followed by addition of iodoacetamide at a final concentration of 25 mm at room temperature for 1 h. Trypsin and LysC were added, each at a ratio of 1:100 (enzyme to total average protein per fraction) and incubated overnight at 37 °C Each fraction was made up of 1% (v/v) trifluoroacetic acid and desalted using Sep-Pak tC18 plates, with peptides eluted in 50% acetonitrile, 0.1% trifluoroacetic acid. Peptides were dried using a GeneVac evaporator and resuspended in 5% formic acid and quantified using a 3-(4-carboxybenzoyl) quinoline-2-carboxaldehyde assay. Five biological replicates were performed in total for each SEC chromatography column used.

For a highly denaturing lysis, cells were lysed in 4% sodium dodecyl sulfate (SDS), 10 mm sodium phosphate (pH 6.0) and 100 mm NaCl, sonicated and filtered as above, followed by separation in a running buffer containing 0.2% SDS on a BioBasic SEC300 column.

##### SDS-PAGE

Aliquots of fractions from SEC runs were pooled into groups of three, made up to 1× lithium dodecyl sulfate sample loading buffer and 25 mm tris(2-carboxyethyl)phosphine (TCEP), heated to 95 °C for 10 min and resolved on 4–12% SDS-PAGE. Gels were run in MES buffer for 45 min at 200 V, then stained for total protein, using SYPRO Ruby, as per manufacturer's protocol.

##### Strong Anion Exchange (SAX)

Procyclic trypanosome cells were prepared in a similar manner as described for SEC analysis, with lysis in 1 ml 20 mm ethanolamine (pH 9.0) containing 0.1 μm TLCK, 1 mm PMSF, 1 μg/ml leupeptin, 1 μg/ml pepstatin and 5 mm EDTA. Lysates were centrifuged and filtered as described previously. Filtered lysate was injected onto a Protein-Pak Hi Res Q, 5 μm, 4.6 × 100 mm, column (Waters, Elstree, U.K.), equilibrated in 20 mm ethanolamine (pH 9.0). Proteins were resolved over a gradient of 0–100% 0.5 m NaCl in 20 mm ethanolamine (pH 9.0), over the course of 26 min, at a flow rate of 0.3 ml/min at 5 °C. Ninety-six 105 μl fractions were collected from 1.5 to 35 min.

Collected fractions were made up to 4% SDS and 25 mm TCEP, then heated to 65 °C for 30 min. Once samples had cooled to room temperature, N-ethylmaleimide was added to a final concentration of 50 mm and incubated for 1 h. The denatured, reduced and alkylated proteins in each fraction were prepared for digestion using a Kingfisher Flex Purification System (ThermoFisher Scientific, San Jose, CA) in combination with magnetic SP3 beads. Twenty microliters of a 1:1 mixture of hydrophobic and hydrophilic, carboxylate modified, Sera-Mag SpeedBead magnetic particles (20 mg/ml in H_2_O, GE) was added to each fraction, followed by the addition of 500 μl of acetonitrile and 15 μl of 10% formic acid. In a 96-well plate format, the Kingfisher Flex System was then used to wash the magnetic beads (protein bound) for each collected fraction, twice in 1 ml 70% ethanol, once in 1 ml 100% acetonitrile and then released into a precooled plate, containing 50 μl 0.1 m Tris-HCl (pH 8.0), 0.1% SDS, 1 mm CaCl_2_ and trypsin and LysC at a 1:100 ratio of protease to estimated protein per fraction. The plate was incubated overnight at 37 °C at 500 rpm in a ThermoMixer (Eppendorf, Hamburg, Germany). Following overnight digestion, the 96-well plate was thoroughly vortexed to ensure resuspension of SeraMag beads and 950 μl of acetonitrile added. Peptides bound to the magnetic beads were washed in 1 ml of acetonitrile, eluted in 40 μl of 2% dimethyl sulfoxide (DMSO) and beads removed from the sample again on the Kingfisher System. Formic acid was added to each sample to a final concentration of 5% and peptide concentration determined using a 3-(4-carboxybenzoyl) quinoline-2-carboxaldehyde assay.

##### LC-MS/MS and Analysis of Spectra

For each biological replicate of either 48 SEC, or 96 SAX fractions, 1 μg of peptide was injected from the most concentrated fraction and the equivalent volume injected for the remaining fractions. Peptides in 5% formic acid were injected onto a C18 nano-trap column using an Ultimate 3000 nanoHPLC system (ThermoFisher Scientific). Peptides were washed with 2% acetonitrile, 0.1% formic acid and resolved on a 150 mm × 75 μm C18 reverse phase analytical column over a gradient from 2–28% acetonitrile over 120 min at a flow rate of 200 nL/min. Peptides were ionized by nano-electrospray ionization at 2.5 kV. Tandem mass spectrometry analysis was carried out on a QExactive+ mass spectrometer (ThermoFisher Scientific), using HCD fragmentation of precursor peptides. A data-dependent method was used, acquiring MS/MS spectra for the top 15 most abundant precursor ions.

SEC RAW data files were analyzed using MaxQuant version 1.5.1.3, with the in-built Andromeda search engine (20, 21), supplied with the *T. brucei brucei* 927 annotated protein database from TriTrypsDB release 8.1, containing 11,567 entries. The mass tolerance was set to 4.5 ppm for precursor ions and MS/MS mass tolerance was set at 20 ppm. The enzyme was set to trypsin and endopeptidase LysC, allowing up to 2 missed cleavages. Carbamidomethyl on cysteine was set as a fixed modification. Acetylation of protein N termini, deamidation of asparagine and glutamine, pyro-glutamate (with N-terminal glutamine), oxidation of methionine and phosphorylation of serine, threonine and tyrosine, were set as variable modifications. Match between runs was enabled, allowing transfer of peptide identifications of sequenced peptides from one LC-MS run to nonsequenced ions, with the same mass and retention time, in another run. A 20-min time window was set for alignment of separate LC-MS runs and a 30-s time window for matching of identifications. The false-discovery rate for protein and peptide level identifications was set at 1%, using a target-decoy based strategy. Each individual SEC fraction was set as an individual experiment in MaxQuant parameters, to output IBAQ data for protein groups in every fraction. Only unique peptides were used for quantitation.

SAX RAW data files were analyzed using MaxQuant version 1.5.3.30, supplied with the *T. brucei brucei* 927 annotated protein database from TriTrypDB release 26.0, also containing 11,567 entries. All other settings were identical, apart from the fixed modification on cysteine, which was set to N-ethylmaleimide. The results can be viewed from the MS-Viewer website (22) by entering the following search keys: SAX: czyi4m7zoe SEC300: esvc3krys1 SEC1000: 5gt8lsrrv7.

##### Data Analysis of Protein Elution Profiles

Data analysis was performed using custom Python scripts, in conjunction with numpy, scikit-learn, pandas and matplotlib libraries. Elution profiles for individual proteins were created using label free quantitation (LFQ) intensities, normalized to the maximum intensity detected across all fractions, using the mean of either four, or three, biological replicates from SEC300 and SEC1000 experiments, respectively. From SEC experiments, proteins were required to be detected with at least one unique peptide found in two biological replicates and with Pearson correlation coefficients among elution profiles >0.6. A detailed report on the protein identified with only one unique peptide is provided in supplemental Table S19.

##### Experimental Design and Statistical Rationale

Five biological replicates were performed for both the SEC300 and SEC1000 experiments, based on the variance detected in previous experiments using SEC based analysis (17, 23). From the five biological replicates performed, one replicate from SEC300 and two replicates from SEC1000 fractionation were discarded from further data analysis, because of reduced reproducibility of elution profiles. One biological replicate was performed for the SAX experiment. The MaxQuant label free quantitation algorithm was used to create elution profiles for individual protein groups identified in each experiment type in each biological replicate (24).

##### Hierarchical Clustering

The mean LFQ profiles for each protein were hierarchically clustered, separately for each experiment type (SEC300, SEC1000 and SAX), using the Euclidean distance measurement and Ward's agglomeration method. The Gene Ontology (GO) term enrichment was computed for each cluster obtained by cutting the dendrogram tree at predetermined distances. Cutting distances from 0 to *n* were evaluated, in which *n* was the cutting distance producing only two clusters. GO term enrichment *p* values were computed with a Fisher test. The Bonferroni correction was applied and only the GO-terms with a *p* value <0.05 were accepted. The cutting distance producing the highest number of enriched GO terms was taken to produce the final clusters for each experiment type.

##### Machine Learning

A pipeline similar to that applied previously for protein correlation profiling (PCP) analysis (18) was used to predict protein complexes, using data from all three experiment types. The protein elution profiles were used to train a random forest predictor implemented with the scikit-learn python package. Protein pairs were scored according to four features, namely: the coapex score, Normalized Cross Correlation (NCC), Pearson Correlation Coefficient (PCC) and STRING scores. The first three features are based purely on the protein elution profiles.

The coapex score (18) is based on the number of biological replicates in which a protein pair shows maximum abundance in the same fraction. The coapex score was derived from the SEC300 and SEC1000 experiments, with four and three biological replicates, respectively, by using the scipy package. For the SEC1000 data, with three biological replicates, the possible coapex scores were: 1 (3 of 3 replicates), 0.6 (2 of 3 replicates), 0.3 (1 of 3 replicates) and 0 (none of the replicates). Similarly, for the SEC300 data, with four biological replicates, the possible coapex scores were 1, 0.75, 0.5, 0.25, and 0.

The NCC was derived in two steps. First, the maximum cross correlation between the two protein profile pairs P_1–2_CC was computed. Then the maximum self-cross-correlation of the first protein profile (P_1_CC) and the maximum self-cross-correlation of the second protein profile (P_2_CC) was determined. The NCC was finally derived as P_1–2_CC/max(P_1_CC, P_2_CC). The PCC was computed as the Pearson correlation score between the two elution profiles.

The PCC and NCC were calculated for the SEC300, SEC1000 and SAX experiments described here, and for experiments produced in (19). This includes ion exchange of mitochondrial extracts (IEX-mito) and cytoplasmic extracts (IEX-cyto) and glycerol gradient fractionation of whole cell lysates (GG-WCL) and mitochondrial extracts (GG-mito). We used the SEQUEST intensity values of the glycerol gradient experiments (GG-WCL, GG-mito), and the MaxQuant intensity values for the ion exchange chromatography experiments (IEX-mito, IEX-cyto) that were retrieved from the supplementary tables of (19). The STRING features (Neighborhood, Fusion, Cooccurence, Coexpression, Experimental, Database, Text Mining) are derived from version 10 of the STRING database (25). The STRING IDs were mapped to the TriTrypDB IDs, and the values were normalized from 0 to 1. The aforementioned scoring features were calculated for all the possible permutations of protein pairs that showed a NCC value greater than 0.15 in at least one of either the SEC300, SEC1000, or SAX experiments, creating a matrix of 609,100 protein pairs with 23 features.

For the machine learning analysis, a data set of “gold standard” true positive peak pairs (GD) was manually assembled. Thirty-one known protein complexes were derived from data deposited in CORUM (26), together with manual addition of protein complexes derived from information in the literature, producing 290 unique true positive pairs of interacting proteins (supplemental Table S1 and S21). A negative data set was extracted by random sampling of the 290 proteins annotated in different complexes (supplemental Table S21). As it would be possible to introduce false negative interactions in this step, the random sampling was repeated 100 times. Finally, using these true positive and true negative test pairs, 100 Random Forest classifiers were assembled based on the same true positive pairs, but with each using a different negative set. Two predictor sets were developed, one set of 100 predictors based on the features derived only from experiments performed in this paper (SEC300, SEC1000 and SAX) and a second set of predictors that implemented all the available features (including STRING, IEX-mito, IEX-cyto, GG-mito and GG-WCL).

All the classifiers were inspected to determine the area under the curve values of the receiver operator curves in 10-fold cross validation. The median values of the probability score outputs of the two 100 classifier sets were used as the final interaction prediction score for the protein pairs.

An interaction prediction score cut-off of 0.75 was used as this threshold selected protein pairs that contained 1% of true negative pairs (*i.e.* a 1% false positive rate). The protein pairs from the two predictor sets were separately fed to the ClusterONE algorithm (27). A search matrix was created for the ClusterONE program with the parameters (0.1 to 1, step 0.1), “haircut” (0.1 to 1, step 0.1) and “s” fixed to 2. The outputs were parsed to derive the parameters that were optimal to obtain the maximum number of GD true positive pairs grouped together. The complexes predicted by ClusterONE using the outputs of the two different predictor sets were merged together, joining predicted complexes that shared two or more proteins. For example, if proteins A-B-C are predicted in a complex in the first set and proteins B-C-D are predicted in the second set, these will be merged to complex A-B-C-D in the final output. All the proteins present in our final machine learning prediction are detected with ≥2 unique peptides in at least one of the experimental data set analyzed.

##### Comparison of Hierarchical Clustering and Machine Learning Predictions

To compare the performance of protein complex prediction between the hierarchical clustering and machine learning methods used in this study, the gold standard complexes (supplemental Table S1) retrieved by the machine learning pipeline, or by the clustering analysis of the SEC300, SEC1000, or SAX data sets were identified. For each experimentally identified gold standard complex, the number of proteins in common with the predicted group (supplemental Table S1) was calculated (COMMON) and divided by the total number of proteins in the predicted gold standard complex to compute the precision/specificity of predictions from each method. The sensitivity/recall was calculated by dividing the COMMON group by the number of proteins in the experimentally predicted complex. A mean was calculated for both precision/specificity and sensitivity/recall from all the gold standard complexes identified in each of the hierarchical clustering or machine learning analyses.

##### Comparison of Protein Complex Predictions to Prior Publications

The clustering results of TbCF-HC net reported in Table S5 in (19) were used for comparison to protein complex predictions made from the machine learning output in this study. Protein complexes in common were identified as sharing two or more proteins between both data sets. Unique protein complexes were identified as sharing one or no proteins between both data sets. The mean Pearson correlation coefficient of elution profiles of all permutated protein pairs within a complex was calculated for both shared and unique complexes. This score was computed from all the data sets presented here (SEC300, SEC1000, and SAX), and from the data sets in (19) (IEX-cyto, IEX-mito, GG-WCL, and GG-mito).

The probability of identifying a pair of protein profiles with a Pearson correlation coefficient greater than 0.7 was assessed for the ion exchange chromatography experiments using a bootstrap analysis. One hundred protein pairs were selected at random from the SAX data set produced in this work, and the IEX-cyto data set from (19), the number of protein pairs with a Pearson correlation coefficients >0.7 was counted, and this process was repeated 100 times.

## RESULTS

### 

#### 

##### SEC and SAX Chromatography of Trypanosoma brucei Lysates

**Fig. 1.**
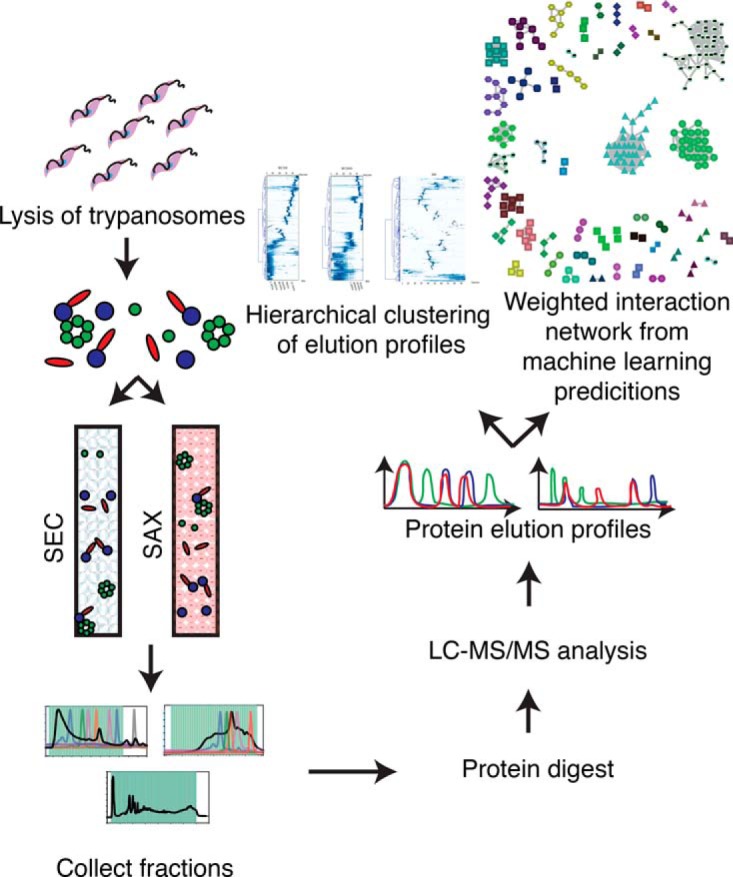
**Workflow for protein correlation profiling.** Lysates were produced containing a mixture of protein complexes, which were separated by either size exclusion chromatography (300 and 1000 Å pore size) or strong anion exchange chromatography. The proteins in each fraction were digested and identified by LC-MS/MS, from which protein elution profiles can be deduced. Putative protein-protein interactions were predicted via both hierarchical clustering of similar elution profiles and through machine learning analysis.

Procyclic form *Trypanosoma brucei brucei* were prepared for native protein complex analysis by resuspension in either ice-cold PBS (for SEC), or 20 mm ethanolamine (for SAX), containing protease inhibitors, followed by sonication lysis. The resulting lysates were centrifuged, filtered, and fractionated, either using BioBasic SEC300, or SEC1000 columns, separating protein complexes based on their size and shape, or a Protein-Pak HiRes SAX column, separating protein complexes based on their charge. The proteins in the fractions from each type of chromatography were reduced, S-alkylated and digested to peptides with trypsin and endopeptidase LysC. After desalting, the resulting peptides were analyzed by LC-MS/MS (Fig. 1).

Protein molecular weight standards were used to characterize the separation ranges of the BioBasic SEC300 and SEC1000 columns, indicating that the SEC300 column has an effective separation range from 8 kDa to 1.2 MDa, whereas the SEC1000 column separates material above 1.2 MDa (supplemental Fig. S1*A* and S1*B*). The retention times of each standard on the SEC300 column were used to generate a linear regression model, allowing the calculation of apparent molecular weights for the proteins and protein complexes found in our data set (supplemental Fig. S1*C*). A separate set of protein standards were used to characterize the resolution and separation of the SAX column (supplemental Fig. S2).

To assess the monomeric molecular weights of proteins eluting across the SEC300 fractionation range, fractions were pooled in groups of three, run on SDS-PAGE under reducing conditions and stained for total protein. Most proteins eluted at a higher apparent molecular weight by native SEC than expected from their monomeric, denatured and reduced molecular weights indicated by SDS-PAGE. This is consistent with many of the individual proteins participating as components of larger complexes. In contrast, when cells were lysed in a highly denaturing buffer, containing 4% SDS and the SEC was carried out in the presence of 0.2% SDS, there was a direct correlation between SEC and SDS-PAGE apparent molecular weights (supplemental Fig. S1*D*).

##### Reproducibility of Mass Spectrometry Based Elution Profiles

The reproducibility of individual biological replicates was assessed for both the SEC300 and SEC1000 experiments (supplemental Fig. S3*A*). Most biological replicates showed high reproducibility, with median Pearson correlation coefficients for each fraction typically >0.75. However, one and two replicates from the five SEC300 and SEC1000 data sets, respectively, deviated significantly and inspection of individual protein elution profiles indicated compromised chromatography (supplemental Fig. S3*B*). The data from these replicates were therefore excluded from further analyses, emphasizing the importance of checking inter-replicate reproducibility before combining data for downstream analysis.

From four biological replicates of SEC300 fractionation experiments, 64,077 peptides were identified, corresponding to 5583 protein groups, detected by at least one unique peptide. From three biological replicates of SEC1000 chromatography, 55,273 peptides were identified, corresponding to 4979 protein groups, detected by at least one unique peptide, and from a single SAX fractionation experiment, 38,135 peptides were detected, corresponding to 3007 protein groups, detected by at least one unique peptide.

##### Hierarchical Clustering of Protein Elution Profiles

**Fig. 2.**
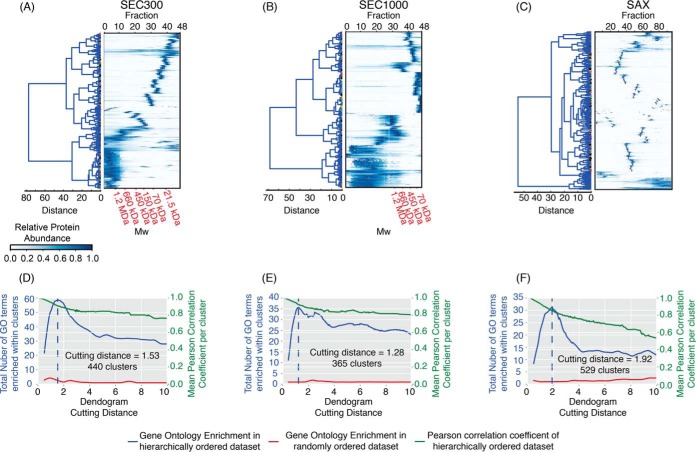
**Hierarchical clustering of protein elution profiles.** Heat-maps of hierarchically clustered elution profiles from lysates separated using either SEC with either (*A*) 300 or (*B*) 1000 Å pore size, and (*C*) SAX chromatography. Panels below the heat-maps demonstrate the effect of varying the dendrogram cutting distance on the mean Pearson correlation coefficient of proteins (green line), and the total number of gene ontology terms enriched (blue line) within clusters in the data from (*D*) SEC300, (*E*) SEC1000, and (*F*) SAX. The red line depicts the number of GO terms enriched within clusters with a random ordering of proteins in each data set.

Several individual protein elution profiles produced two peaks by SEC300 chromatography. The generally lower intensity lower molecular weight peaks likely correspond to protein degradation products or protein monomers/dimers that are in equilibrium with the higher molecular weight complex in which they appear. Because the hierarchical clustering algorithm selects for the most intense peak in a protein profile, these lower molecular weight peaks generally do not feature in the clustering analysis. Hierarchical clustering of all protein elution profiles was performed for each data set separately (Fig. 2*A*–2*C*); by cutting the resulting dendrograms, it was possible to define groups of proteins that have similar elution profiles and hence potentially interact. A range of cutting distances was simulated, and the within-cluster Gene Ontology (GO) term enrichment and the mean Pearson correlation coefficients of elution profiles were observed (Fig. 2*D*–2*F*). As the number of clusters was reduced, a sharp increase in the number of enriched GO terms was observed in all data sets, as functionally associated proteins were grouped together within a cluster. As clusters became larger and unrelated proteins were grouped together, the number of enriched GO terms decreased. The cutting distance producing the highest GO term enrichment within clusters across the data set for each form of fractionation was selected. Thus, for the SEC300, SEC1000, and SAX experiments, cutting distances of 1.53, 1.28, and 1.92 were chosen, producing, respectively, 440, 365, and 529 clusters of proteins. As a control, the order of proteins within the dendrograms of each data set was shuffled randomly and then the same analyses performed; under these conditions, a similar increase in GO term enrichment across cutting distance was not apparent (Fig. 2*D*–2*F*).

##### Characterization of Known and Highly Conserved Complexes

**Fig. 3.**
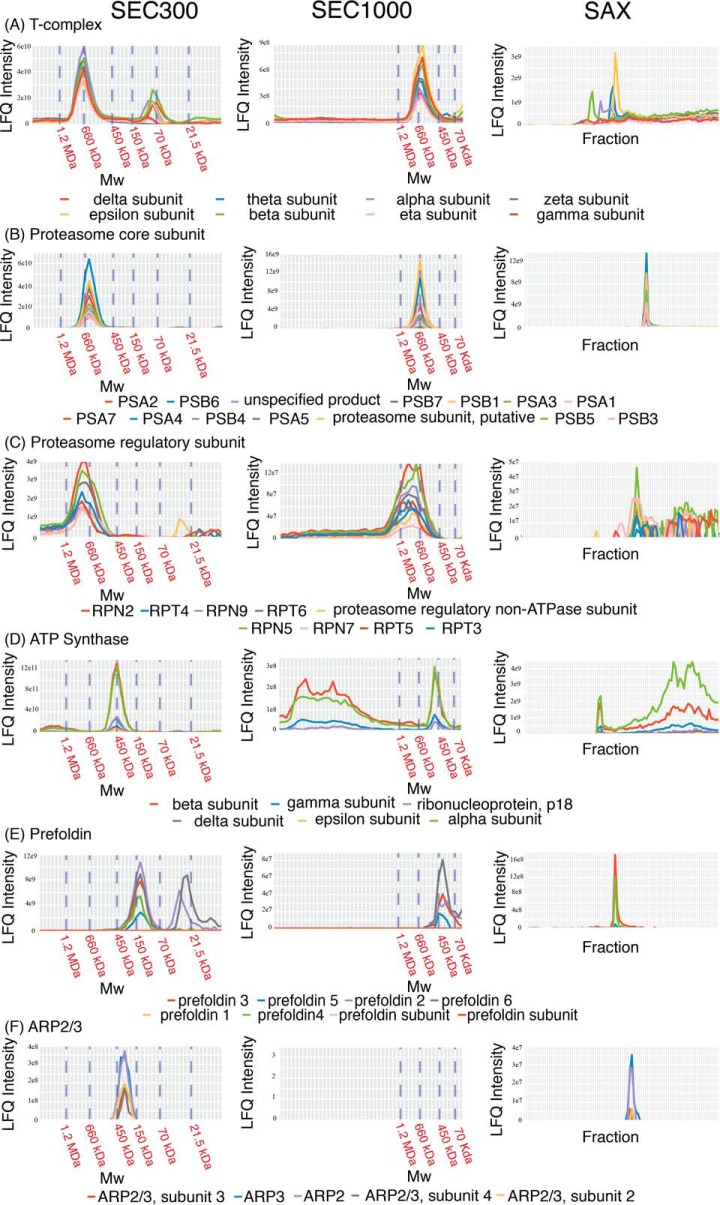
**Elution profiles of components of known protein complexes.** Proteins predicted to be in (*A*) proteasome core subunit, (*B*) proteasome regulatory subunit, (*C*) T-complex, (*D*) ATP synthase, (*E*) prefoldin, or (*F*) ARP 2/3 are plotted displaying their detected LFQ intensities across the fractionation ranges of SEC300 and/or SEC1000 and/or SAX chromatography columns.

To validate the assumption that protein cochromatography correlates with protein association in the data sets, the elution profiles of proteins expected to be present as stable complexes (either according to the *T. brucei* literature or by analogy with highly conserved complexes in other organisms) were inspected (Fig. 3). With respect to the former, the proteasome regulatory cap components all eluted with a peak at ∼770 kDa and the proteasome core components all eluted with a peak at ∼660 kDa (Fig. 3*A* and 3*B*), consistent both with the detection of stable complexes via cochromatography and with previous reports showing that the proteasome core and caps dissociate from each other in *T. brucei* lysates (28). With respect to other highly conserved protein complexes not previously characterized in *T. brucei*, cochromatography of the predicted subunits of the chaperonin T-complex, ATP synthase, the prefoldin chaperone complex and the ARP2/3 complex was also observed (Fig. 3*C*–3*F*, respectively).

In some instances, (*e.g.* the proteasome regulatory complex and the T-complex) the protein complexes were less stable when subjected to SAX chromatography than to SEC (Fig. 3*B* and 3*C*). This was not unexpected as SAX chromatography was performed at pH 9.0 and involves elution with a salt gradient, whereas SEC was performed at a more physiological pH and ionic strength. These observations were, therefore, interpreted as being consistent with the dissociation of a subset of native protein complexes when exposed to high salt and high pH during SAX chromatography.

Taken together, these PCP-MS data using trypanosome extracts confirmed that cochromatography and mass spectrometric protein identification and quantification can be used to provide evidence for the physical association of proteins in complexes.

##### Machine Learning Analysis to Predict Protein Complexes

**Fig. 4.**
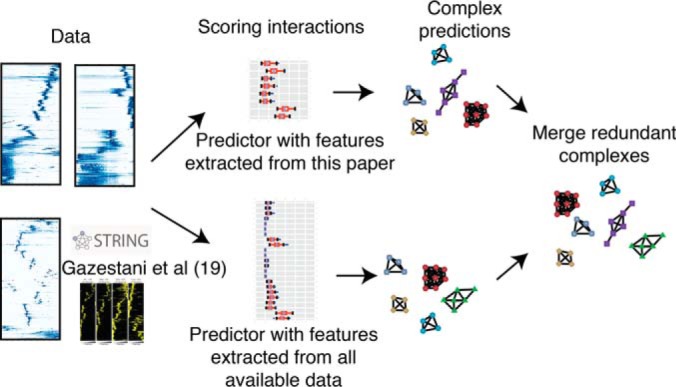
**Machine learning protein complex prediction pipeline.** Data produced either solely in this manuscript, or including data from STRING and other *Trypanosoma brucei* interactome publications (19), were used to train two sets of random forest predictors to score binary protein-protein interactions. The interaction prediction scores were used to predict protein complexes separately for each predictor, then merged to join redundant complexes.

To predict the likelihood of binary interactions among all pairs of coeluting proteins detected in our data sets, a scoring methodology was designed to quantify the similarity of elution patterns. Two random forest predictors were implemented. The first predictor was trained with features extracted from data produced in this project. The second predictor was trained by combining the first set of features with features extracted from a recently published interactome study in *T. brucei* (19). We also added to the second predictor, features retrieved from version 10 of the STRING interaction database (25), with the intention of promoting interacting protein pairs with orthogonal evidence for interaction from the literature (Fig. 4). Both predictors were trained using 31 protein complexes, comprising 290 true positive interaction pairs (supplemental Table S1 and S21), and 100 sets of randomly selected true negative interaction pairs (supplemental Table S21). At an interaction prediction score >0.75 there was a false positive rate <1% (supplemental Figs. S4*A* and S4*B*), hence this was used as the threshold for positive interaction across the whole data set. Receiver Operator Characteristic curves also demonstrate the high performance of the machine learning method (supplemental Fig. S4*C*).

**Fig. 5.**
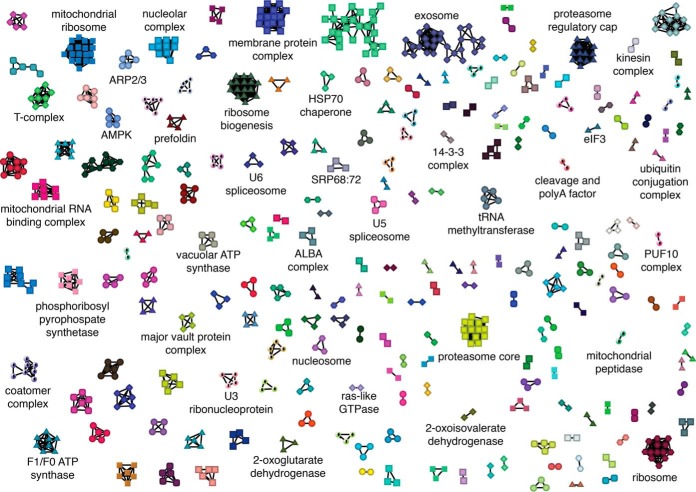
**Machine learning predictions of protein complexes.** ClusterOne output of merged predictions from machine learning with both sets of predictors. Known protein complexes, or complexes with proteins of similar function have been manually annotated.

Analysis of how often the random forest predictors used each feature to classify positive and negative interactions showed that the SEC300 coapex score, cross-correlation and Pearson correlation and SEC1000 coapex features had the highest predictive power (supplemental Fig. S4*D*). The outputs of the two random forest predictors were fed to ClusterOne (an algorithm used to identify protein complexes in protein-protein interaction networks (18, 27)) to derive two sets of predicted protein complexes. These two complex predictions were merged to assemble 234 predicted protein complexes, encompassing 805 proteins, with complexes ranging from 2–18 protein subunits (supplemental Fig. S5). We were able to rediscover again 28 out of the 32 gold standard protein complexes (supplemental Table S20) and we have ascribed either a putative function, or name, to the complexes when they contain proteins of either known or suggested biological function in the TriTrypDB genome database (Fig. 5, supplemental Tables S2 and S3) (29). Although, as expected, many of the predicted complexes are abundant core protein complexes conserved across eukaryotic evolution, we also detected novel complexes and protein-protein interactions not previously described in *T. brucei*. Some of these predicted interactions shed light on the functions of some of the many “hypothetical” proteins in the trypanosome genome. Some examples of previously uncharacterized associations (supplemental Figs. S6 and S7) are described in the Discussion.

##### Comparison of Hierarchical Clustering and Machine Learning

To compare the output from the hierarchical clustering and machine learning methods of predicting protein complexes presented in this study, the precision and sensitivity of each method in predicting the 31 gold standard complexes was calculated (supplemental Fig. S8 and supplemental Table S1). Protein complex predictions derived from the machine learning method clearly outperform hierarchical clustering of the SEC300, SEC1000, and SAX data sets in terms of both precision (mean value of 0.7) and sensitivity (mean value of 0.88). Hierarchical clustering of the SEC300 data set performs similarly to machine learning in terms of mean sensitivity (0.79), but has a much lower mean precision (0.46), indicating an ability to exclude false positive interactions, but missing out on many true positive interactions. Hierarchical clustering of SEC1000 or SAX data sets appear to produce similarly low mean values of precision (0.42 and 0.36 respectively), and perform worse than SEC300 in regard to sensitivity (0.58 each).

##### Comparison of Protein Complex Predictions to Published Data Sets

The comparison to TbCF-HC net, published in (19), reveals that 40 protein complexes (with a minimum of two proteins in common) are detected in common with the data set published here (supplemental Fig. S9). A further 90 protein complexes predicted in TbCF-HC net are not corroborated in our data set, and 190 are predicted in our data set and not corroborated in TbCF-HC net. For complexes uniquely predicted in TbCF-HC net, the mean Pearson correlation coefficient of elution profiles of constituent proteins within complexes, is below 0.5 when computed from the SEC300, SEC1000, and SAX data sets, and is below 0.7 when computed from IEX-cyto, IEX-mito, GG-WCL, and GG-mito (19) (supplemental Fig. S9). Focusing on complexes predicted in common between both data sets, or complexes unique to the data presented here, the SEC300 and SEC1000 experiments outperform all others, with the highest mean Pearson correlation of elution profiles within protein complexes (supplemental Fig. S9).

Furthermore, the SAX experiment also outperforms the IEX experiments performed in (19), in regard to mean Pearson correlation coefficients in complexes predicted in common to both data sets and unique from data presented here. Random sampling of elution profiles of the IEX-cyto experiment highlights that ∼20% of protein elution profiles will have a Pearson correlation coefficient >0.7, in comparison to ∼3% of elution profiles in the comparable SAX experiment (supplemental Fig. S9). This indicates that many elution profiles in the IEX-cyto experiment will have a similar shape, and hence appear to coelute, just by chance, negatively impacting the quality of the detected protein complexes. We think that this effect is related to the smaller number of elution fractions analyzed by Gazestani *et al.* (19 fractions) in comparison to our SAX experiments (96 fractions).

##### Data Visualization

**Fig. 6.**
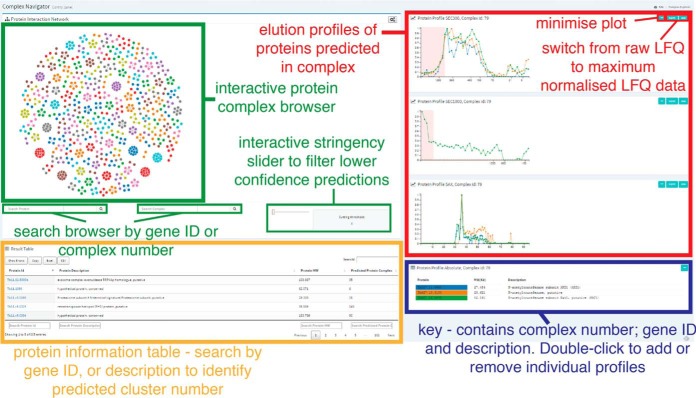
**Data visualization tools - Complex Explorer.** Interactive web visualization tool which allows users to dynamically browse high confidence protein complexes and protein-protein interactions, predicted through machine learning.

All of the processed MS and chromatography data and predictions have been made freely available via a custom, searchable database. The data can be browsed on a web server at (http://134.36.66.166:8083/complex_explorer). Thanks to a user-friendly graphical interface, researchers can conveniently explore and display all the predicted complexes and elution profiles reported in this study. There are three distinct applications with which to browse the data:

The “Complex Explorer” (Fig. 6) is a dynamic browser of the high-confidence predictions of protein complexes, derived from machine learning of exhaustive pairwise comparisons of all protein elution profiles, matching the data presented in (Fig. 5) and (supplemental Table S2). Each cluster is displayed as a network of interconnected nodes (protein groups), with the lines among the nodes indicating evidence of pairwise associations. The stringency (cutting threshold), for the associations can be varied with a slider below the browser and the cluster browser can be queried to highlight individual protein groups and cluster numbers. Mousing over the nodes brings up their GO-terms within a word cloud of the GO-terms for the other nodes in the complex, which can give a general impression of possible cluster function. The dynamic browser can be adjusted through “*settings*” to highlight any or all of the following:

- Nodes with human homologs
- Nodes identified as essential in cell culture (4)
- Nodes that were used as “gold-standards” for the machine-learning
- Clusters which agree with homologous associations in the STRING database (using a relatively high combined STRING score threshold value of >950)
- Clusters that are inter-related by the same STRING associations
- Clusters predicted by those STRING associations alone
- Nodes which appear in more than one cluster

Clicking on any node in a cluster brings up a table of the protein group components of that cluster, alongside the SEC300, SEC1000, and SAX chromatograms for those protein groups. The chromatograms are dynamic and can be expanded in the *x*- (time) axis and display either raw or normalized LFQ intensity data on the *y* axis. Further, the color-coded elution profiles of individual protein groups can be switched on or off by double-clicking on the gene IDs below the chromatograms. Below the dynamic cluster browser is a table of all the nodes in the browser, which can be searched in various ways and downloaded by the user for other applications.

The second application, “Profile Explorer” (supplemental Fig. S10) allows exploration of any potential protein-protein association of the user's choosing. The application allows the input of a list of up to twenty TriTrypsDB gene IDs and outputs the associated fractionation data in any of the SEC300, SEC1000 and SAX data sets, as well as in the density gradient and ion exchange fractionations recently published in (19).

The “Cluster Explorer” (supplemental Fig. S11) application allows exploration of putative protein-protein associations based on hierarchical clustering of the protein elution profiles from SEC300, SEC1000, and SAX chromatography (Fig. 2). These are lower confidence predictions of protein-protein associations than those based on machine learning, but they allow the user to ask whether there is any evidence for the *possible* association of two or more proteins by cochromatography. Thus, the application allows the input of a list of up to twenty TriTrypDB gene IDs and outputs graphs showing which hierarchical clusters they belong to. From there, selecting the cluster number will display the relevant chromatogram.

In addition, all the mass spectrometry proteomics data have been deposited with the ProteomeXchange Consortium via the PRIDE (30) partner repository with the data set identifier PXD005968.

## DISCUSSION

Information has been produced on the elution profiles across SEC and SAX chromatograms for 5845 protein groups identified in trypanosome extracts using quantitative MS-based proteomics. Computational analysis of these elution profiles allows us to predict 234 protein complexes, each containing between two and eighteen protein groups. Of these complexes, 77 contain at least one protein annotated as “hypothetical” and 19 are composed solely of hypothetical proteins, with no other orthogonal information on protein function. These data are provided, together with those of Gazestani and colleagues (19), in an open access, online database that can be browsed and queried. This provides a useful resource for trypanosome biologists and protein biochemists studying complexes and protein-protein interactions in other organisms.

In this study, protein complex predictions are produced using two distinct methodologies; hierarchical clustering of independent fractionation experiments, or machine learning, using scoring features derived from all of our fractionation experiments, together with orthogonal data from STRING and previously published *T. brucei* fractionation data sets (19). The comparison of these distinct methods of protein complex prediction indicates that machine learning is the most stringent and accurate, with the highest precision and specificity (supplemental Fig. S8). Therefore, the following discussion focuses on the protein complex predictions from the machine learning analysis. However, we believe that the individual protein elution profiles and hierarchically clustered data sets are of use to the wider *T. brucei* research community, providing wider groupings of proteins which are coeluting across one of the three fractionation methods used, that may still provide evidence for protein-protein interaction. To this end, these data sets and the predicted clusters are available for researchers to look at on our searchable online database, together with the machine learning predictions.

Novel insights into subunits of trypanosome protein complexes identified here include the proteasome core (complex 31) and regulatory unit (complex 30), the prefoldin complex (complex 29), the chaperonin T-complex (complex 129), AMPK (complex 3), vacuolar ATP synthase (complex 20), the exosome (complex 28), subcomponents of the spliceosome (complexes 86 and 180), the nucleosome (complex 87), ARP2/3 (complex 112), F_1_F_0_ ATP synthase (complex 130), and several others (supplemental Tables S2 and S3). Although these are all conserved eukaryotic protein complexes whose presence may, therefore, be expected in trypanosomes, the underlying complex protein group compositions also suggests novel components. For example, although most of the proteins detected in the proteasome complex (complex 31; supplemental Table S4) are annotated as proteasome alpha and beta subunits, one, at the time of this analysis, was annotated as “unspecified product” (Tb927.9.11310). A bespoke BLASTp search subsequently revealed that this gene product has 100% homology with the 20S proteasome beta subunit of *T. brucei gambiense* (XP_011776865.1). Thus, the cochromatography of Tb927.9.11310 with the proteasome core complex provided the impetus to re-evaluate its identity.

Another example is provided by complex 130 (Supplemental Table 5). This contains all the characterized subunits (α, β, γ, δ, and ε) of the F_1_ domain of the F_0_F_1_-ATP synthase complex, as well as ribonucleoprotein p1 (the b subunit of the F_0_ domain) (31), but also contains two other proteins (Tb927.11.13070 and Tb927.3.3410), which we therefore postulate are components of the *T. brucei* F_0_F_1_-ATP synthase complex. Additionally, complex 85 (supplemental Table S6) comprises three hypothetical proteins, two of which have been annotated by (31) as trypanosome specific ATP synthase components, but the third, Tb927.5.1780, has no functional annotation and may be a further novel component of the F_0_F_1_-ATP synthase.

Previous affinity purification-MS analyses of the mitochondrial ribosome of *T. brucei* identified 133 proteins, 77 of which were classed as large-subunit and 56 as small-subunit associated proteins (32). Twenty-six of these components were identified in complexes 4, 92, and 134 (supplemental Tables S7–S9), plus one hypothetical protein (Tb927.7.3030) in complex 134, suggesting this may be a novel mitochondrial ribosome subunit.

Complex 3 contains experimentally verified members (Tb927.8.2450 and Tb927.10.3700, β and γ subunits respectively) of the AMP-dependent protein kinase (AMPK) complex (supplemental Table S10) (33). It also contains Tb927.3.4560, annotated as the AMPKα subunit, Tb927.10.5310, a SNF1 related protein kinase with homology to AMPKα, and Tb927.9.9270, a hypothetical protein with little functional information. AMPK is generally a heterotrimeric complex, therefore it is possible that the two putative AMPKα subunits (Tb927.3.4560 and Tb927.10.5310) are isoforms, forming part of two independent AMPK protein complexes which coelute. Whether the hypothetical protein Tb927.9.9270 is either a novel component of the trypanosome AMPK complex, is a subunit isoform, or is simply a contaminating coeluted protein, warrants further investigation.

The aforementioned examples suggest some novel components of conserved complexes. Other examples either suggest or confirm trypanosome-specific protein-protein interactions. This is illustrated by our observation of the consistent coelution of the Pumillo homology domain protein PUF10 with a hypothetical protein (Tb927.7.2170) across SEC300, SEC1000, and SAX fractionation in complex 72 (supplemental Table S11; supplemental Fig. S6*A*). PUF proteins are known to bind to mRNA and the hypothetical protein has also been predicted to bind mRNA through capture on oligo(dT) beads (34). Taken together, these data indicate that Tb927.7.2170 interacts with PUF10 and may play a role in mRNA regulation.

In complex 174 two proteins were detected; a hypothetical protein (Tb927.8.1960) and subunit 10 of the CCR4-NOT complex (supplemental Table S12; supplemental Fig. S6*B*) (35). The hypothetical protein has recently been copurified with CAF1, a core component of the CAF1-NOT deadenylase complex (36) and suggested to be the homolog of a human protein (C2ORF29), which was recently classified as subunit 11 of the CCR4-NOT complex that interacts with subunit 10 (37). Thus, the cochromatography of Tb927.8.1960 with CCR4-NOT subunit 10 provides further evidence for its functional classification as subunit 11 of the trypanosome CCR4-NOT complex.

In complex 108, the trypanosome periodic tryptophan protein Pwp2 coelutes with Tb927.11.10480, Tb927.11.460, and Tb927.7.4220 that, on inspection, contain C-terminal Utp21, Utp13, and Utp12 domains, respectively, (supplemental Table S13; supplemental Fig. S6*C*). Research with yeast has shown that Pwp2 is known to associate with four other proteins (containing the same C-terminal Utp domains) in the U3 ribonucleoprotein assembly machinery, forming a complex necessary for pre-18S rRNA processing (38). It is therefore possible that complex 108 may perform a similar pre-18S rRNA processing function in *T. brucei*.

Three proteins were detected in complex 76: Tb927.10.170 (pseudouridine synthase, Cbf5p), Tb927.4.470 (snoRNP protein, GAR1 putative), and Tb927.4.750 (50S ribosomal protein L7Ae, putative) (supplemental Table S14; supplemental Fig. S6*D*). Cbf5 is the enzymatic component of the H/ACA ribonucleoprotein complex, which pseudouridylates target RNAs. In other eukaryotes, there are many H/ACA ribonucleoprotein complexes, formed through the interaction of different RNAs with the same four core proteins (Cbf5, Gar1, Nhp2 and Nop10) (39). The coelution of the three trypanosome proteins suggests that H/ACA ribonucleoprotein complexes exist in trypanosomatids and supports the putative identity of Tb927.4.470 as a Gar1 homolog. A BLASTp search of the putative ribosomal subunit, Tb927.4.750, also indicates homology to Nhp2, further supporting the identity of this complex. A search of TriTrypDB indicates that there is one annotated Nop10 homolog (Tb927.10.4740) in *T. brucei*. Although this protein was not detected in our high-confidence protein complex prediction, using our “Profile Explorer” data visualization tool, we can see this protein is detected in our SAX fractionation experiment, where it coelutes with the three other components of complex 76.

Previously published studies have demonstrated the constitutive interaction of a heat-shock protein 90 (HSP90), with protein phosphatase 5 (PP5) in *T. brucei* (40). Complex 12 contains five proteins, including an HSP90 (Tb927.3.3580), and the PP5 previously demonstrated to interact with HSP90 (Tb927.10.13670) (supplemental Table S15; supplemental Fig. S6*A*). We also observed the association of these proteins with a putative HSP70 protein (Tb927.9.9860). HSP90 chaperones function through their association with HSP70 proteins, which recruit and transfer substrate proteins to HSP90 (41). These associations, therefore, match our understanding of HSP90 function and identifies a putative function for a previously uncharacterized HSP70.

In complex 99 there is an association of proteins from two distinct protein complexes (supplemental Table S16; supplemental Fig. S6*B*). Two proteins have been experimentally verified as members of the spliced leader RNA cap methyltransferase (42), including the catalytic MTR1 subunit, and a hypothetical protein (Tb927.11.16490), whereas the other three proteins are translation elongation factors. These associations suggest an interesting link between the methyltransferase capping enzymes of spliced leader RNA and the mRNA translation machinery.

Complex 165 (supplemental Table S17; supplemental Fig. S6*C*) is dominated by nucleolar associated proteins but also contains two arginine-N-methyltransferases (TbPRMT1 and TbPRMT3). The latter two enzymes belong to the same complex and are mutually dependent on each other for stability (43, 44). The association of these enzymes with nucleolar proteins suggests they may have some function in pol1-mediated transcription.

In complex 164 (supplemental Table S18; supplemental Fig. S6*D*) the presence of two subunits of the GPI transamidase, together with signal peptidase, suggests an association between GPI transamidase and the translocon complex in the endoplasmic reticulum (ER) (45). The suggested colocation of these components is novel, but consistent with the known cotranslational addition of GPI anchors to nascent proteins in *T. brucei* (46). Interestingly, two other known ER proteins are also present in this complex: Dol-P-Man synthase and 3-keto-dihydrosphingosine reductase.

In summary, the trypanosome PCP-MS data presented here provide a valuable new resource for the research community to find and characterize either novel components of known complexes and/or to assess whether individual proteins of interest appear in larger complexes. The open access availability of the data via an interactive, online database should facilitate such searches. We also refer the interested reader to the recently published TrypsNetDB (47).

## DATA AVAILABILITY

All mass spectrometry data have been deposited with the ProteomeXchange Consortium via the PRIDE partner repository with the dataset identifier PXD005968, https://www.ebi.ac.uk/pride/archive/. Processed data and data exploration tools can be found at http://134.36.66.166:8083/complex_explorer. Annotated spectra can be viewed from the MS-Viewer website (http://msviewer.ucsf.edu/prospector/cgi-bin/msform.cgi?form=msviewer) by entering the following search keys: SAX: czyi4m7zoe; SEC300: esvc3krys1; SEC1000: 5gt8lsrrv7.

## Supplementary Material

Supplemental Data

## Footnotes

^1^ The abbreviations used are:

PCP: protein correlation profiling
SEC: size exclusion chromatography
IEX: ion exchange chromatography
SAX: strong anion exchange
TLCK: 1–5-chloro-3-tosylamido-7-amino-2-heptone
TCEP: tris(2-carboxyethyl)phosphine
IBAQ: intensity based absolute quantification
LFQ: label free quantitation
GO: gene ontology
NCC: normalized cross correlation
PCC: Pearson correlation coefficient.

## References

[1] Franco J. R., Simarro P. P., Diarra A., and Jannin J. G. (2014) Epidemiology of human African trypanosomiasis. Clin. Epidemiol. 6, 257–275 25125985 10.2147/CLEP.S39728 PMC4130665

[2] Berriman M., Ghedin E., Hertz-Fowler C., Blandin G., Renauld H., Bartholomeu D. C., Lennard N. J., Caler E., Hamlin N. E., Haas B., Bohme U., Hannick L., Aslett M. A., Shallom J., Marcello L., Hou L., Wickstead B., Alsmark U. C., Arrowsmith C., Atkin R. J., Barron A. J., Bringaud F., Brooks K., Carrington M., Cherevach I., Chillingworth T. J., Churcher C., Clark L. N., Corton C. H., Cronin A., Davies R. M., Doggett J., Djikeng A., Feldblyum T., Field M. C., Fraser A., Goodhead I., Hance Z., Harper D., Harris B. R., Hauser H., Hostetler J., Ivens A., Jagels K., Johnson D., Johnson J., Jones K., Kerhornou A. X., Koo H., Larke N., Landfear S., Larkin C., Leech V., Line A., Lord A., Macleod A., Mooney P. J., Moule S., Martin D. M., Morgan G. W., Mungall K., Norbertczak H., Ormond D., Pai G., Peacock C. S., Peterson J., Quail M. A., Rabbinowitsch E., Rajandream M. A., Reitter C., Salzberg S. L., Sanders M., Schobel S., Sharp S., Simmonds M., Simpson A. J., Tallon L., Turner C. M., Tait A., Tivey A. R., Van Aken S., Walker D., Wanless D., Wang S., White B., White O., Whitehead S., Woodward J., Wortman J., Adams M. D., Embley T. M., Gull K., Ullu E., Barry J. D., Fairlamb A. H., Opperdoes F., Barrell B. G., Donelson J. E., Hall N., Fraser C. M., Melville S. E., and El-Sayed N. M. (2005) The genome of the African trypanosome Trypanosoma brucei. Science 309, 416–422 16020726 10.1126/science.1112642

[3] Salavati R., and Najafabadi H. S. (2010) Sequence-based functional annotation: what if most of the genes are unique to a genome? Trends Parasitol. 26, 225–229 20211583 10.1016/j.pt.2010.02.001

[4] Alsford S., Turner D. J., Obado S. O., Sanchez-Flores A., Glover L., Berriman M., Hertz-Fowler C., and Horn D. (2011) High-throughput phenotyping using parallel sequencing of RNA interference targets in the African trypanosome. Genome Res. 21, 915–924 21363968 10.1101/gr.115089.110 PMC3106324

[5] Alberts B. (1998) The cell as a collection of protein machines: preparing the next generation of molecular biologists. Cell 92, 291–294 9476889 10.1016/s0092-8674(00)80922-8

[6] Gavin A. C., Bosche M., Krause R., Grandi P., Marzioch M., Bauer A., Schultz J., Rick J. M., Michon A. M., Cruciat C. M., Remor M., Hofert C., Schelder M., Brajenovic M., Ruffner H., Merino A., Klein K., Hudak M., Dickson D., Rudi T., Gnau V., Bauch A., Bastuck S., Huhse B., Leutwein C., Heurtier M. A., Copley R. R., Edelmann A., Querfurth E., Rybin V., Drewes G., Raida M., Bouwmeester T., Bork P., Seraphin B., Kuster B., Neubauer G., and Superti-Furga G. (2002) Functional organization of the yeast proteome by systematic analysis of protein complexes. Nature 415, 141–147 11805826 10.1038/415141a

[7] Ho Y., Gruhler A., Heilbut A., Bader G. D., Moore L., Adams S. L., Millar A., Taylor P., Bennett K., Boutilier K., Yang L., Wolting C., Donaldson I., Schandorff S., Shewnarane J., Vo M., Taggart J., Goudreault M., Muskat B., Alfarano C., Dewar D., Lin Z., Michalickova K., Willems A. R., Sassi H., Nielsen P. A., Rasmussen K. J., Andersen J. R., Johansen L. E., Hansen L. H., Jespersen H., Podtelejnikov A., Nielsen E., Crawford J., Poulsen V., Sorensen B. D., Matthiesen J., Hendrickson R. C., Gleeson F., Pawson T., Moran M. F., Durocher D., Mann M., Hogue C. W., Figeys D., and Tyers M. (2002) Systematic identification of protein complexes in Saccharomyces cerevisiae by mass spectrometry. Nature 415, 180–183 11805837 10.1038/415180a

[8] Gavin A. C., Aloy P., Grandi P., Krause R., Boesche M., Marzioch M., Rau C., Jensen L. J., Bastuck S., Dumpelfeld B., Edelmann A., Heurtier M. A., Hoffman V., Hoefert C., Klein K., Hudak M., Michon A. M., Schelder M., Schirle M., Remor M., Rudi T., Hooper S., Bauer A., Bouwmeester T., Casari G., Drewes G., Neubauer G., Rick J. M., Kuster B., Bork P., Russell R. B., and Superti-Furga G. (2006) Proteome survey reveals modularity of the yeast cell machinery. Nature 440, 631–636 16429126 10.1038/nature04532

[9] Krogan N. J., Cagney G., Yu H., Zhong G., Guo X., Ignatchenko A., Li J., Pu S., Datta N., Tikuisis A. P., Punna T., Peregrin-Alvarez J. M., Shales M., Zhang X., Davey M., Robinson M. D., Paccanaro A., Bray J. E., Sheung A., Beattie B., Richards D. P., Canadien V., Lalev A., Mena F., Wong P., Starostine A., Canete M. M., Vlasblom J., Wu S., Orsi C., Collins S. R., Chandran S., Haw R., Rilstone J. J., Gandi K., Thompson N. J., Musso G., St Onge P., Ghanny S., Lam M. H., Butland G., Altaf-Ul A. M., Kanaya S., Shilatifard A., O'Shea E., Weissman J. S., Ingles C. J., Hughes T. R., Parkinson J., Gerstein M., Wodak S. J., Emili A., and Greenblatt J. F. (2006) Global landscape of protein complexes in the yeast Saccharomyces cerevisiae. Nature 440, 637–643 16554755 10.1038/nature04670

[10] Hu P., Janga S. C., Babu M., Diaz-Mejia J. J., Butland G., Yang W., Pogoutse O., Guo X., Phanse S., Wong P., Chandran S., Christopoulos C., Nazarians-Armavil A., Nasseri N. K., Musso G., Ali M., Nazemof N., Eroukova V., Golshani A., Paccanaro A., Greenblatt J. F., Moreno-Hagelsieb G., and Emili A. (2009) Global functional atlas of Escherichia coli encompassing previously uncharacterized proteins. PLoS Biol. 7, e96 19402753 10.1371/journal.pbio.1000096 PMC2672614

[11] Andersen J. S., Wilkinson C. J., Mayor T., Mortensen P., Nigg E. A., and Mann M. (2003) Proteomic characterization of the human centrosome by protein correlation profiling. Nature 426, 570–574 14654843 10.1038/nature02166

[12] Dunkley T. P., Watson R., Griffin J. L., Dupree P., and Lilley K. S. (2004) Localization of organelle proteins by isotope tagging (LOPIT). Mol. Cell. Proteomics 3, 1128–1134 15295017 10.1074/mcp.T400009-MCP200

[13] Foster L. J., de Hoog C. L., Zhang Y., Zhang Y., Xie X., Mootha V. K., and Mann M. (2006) A mammalian organelle map by protein correlation profiling. Cell 125, 187–199 16615899 10.1016/j.cell.2006.03.022

[14] Li S. S., and Giometti C. S. (2007) A combinatorial approach to studying protein complex composition by employing size-exclusion chromatography and proteome analysis. J. Separation Sci. 30, 1549–1555 10.1002/jssc.200700011 17623436

[15] Olinares P. D., Ponnala L., and van Wijk K. J. (2010) Megadalton complexes in the chloroplast stroma of Arabidopsis thaliana characterized by size exclusion chromatography, mass spectrometry, and hierarchical clustering. Mol. Cell. Proteomics 9, 1594–1615 20423899 10.1074/mcp.M000038-MCP201 PMC2938090

[16] Kristensen A. R., Gsponer J., and Foster L. J. (2012) A high-throughput approach for measuring temporal changes in the interactome. Nat. Methods 9, 907–909 22863883 10.1038/nmeth.2131 PMC3954081

[17] Kirkwood K. J., Ahmad Y., Larance M., and Lamond A. I. (2013) Characterization of native protein complexes and protein isoform variation using size-fractionation-based quantitative proteomics. Mol. Cell. Proteomics 12, 3851–3873 24043423 10.1074/mcp.M113.032367 PMC3861729

[18] Havugimana P. C., Hart G. T., Nepusz T., Yang H., Turinsky A. L., Li Z., Wang P. I., Boutz D. R., Fong V., Phanse S., Babu M., Craig S. A., Hu P., Wan C., Vlasblom J., Dar V. U., Bezginov A., Clark G. W., Wu G. C., Wodak S. J., Tillier E. R., Paccanaro A., Marcotte E. M., and Emili A. (2012) A census of human soluble protein complexes. Cell 150, 1068–1081 22939629 10.1016/j.cell.2012.08.011 PMC3477804

[19] Gazestani V. H., Nikpour N., Mehta V., Najafabadi H. S., Moshiri H., Jardim A., and Salavati R. (2016) A Protein Complex Map of Trypanosoma brucei. PLoS Neglected Tropical Dis. 10, E0004533 10.1371/journal.pntd.0004533 PMC4798371 26991453

[20] Cox J., and Mann M. (2008) MaxQuant enables high peptide identification rates, individualized p.p.b.-range mass accuracies and proteome-wide protein quantification. Nature Biotechnol. 26, 1367–1372 19029910 10.1038/nbt.1511

[21] Cox J., Neuhauser N., Michalski A., Scheltema R. A., Olsen J. V., and Mann M. (2011) Andromeda: a peptide search engine integrated into the MaxQuant environment. J. Proteome Res. 10, 1794–1805 21254760 10.1021/pr101065j

[22] Baker P. R., and Chalkley R. J. (2014) MS-viewer: a web-based spectral viewer for proteomics results. Mol. Cell. Proteomics 13, 1392–1396 24591702 10.1074/mcp.O113.037200 PMC4014294

[23] Larance M., Kirkwood K. J., Tinti M., Brenes Murillo A., Ferguson M. A., and Lamond A. I. (2016) Global Membrane Protein Interactome Analysis using In vivo Crosslinking and Mass Spectrometry-based Protein Correlation Profiling. Mol. Cell. Proteomics 15, 2476–2490 27114452 10.1074/mcp.O115.055467 PMC4937518

[24] Cox J., Hein M. Y., Luber C. A., Paron I., Nagaraj N., and Mann M. (2014) Accurate proteome-wide label-free quantification by delayed normalization and maximal peptide ratio extraction, termed MaxLFQ. Mol. Cell. Proteomics 13, 2513–2526 24942700 10.1074/mcp.M113.031591 PMC4159666

[25] Szklarczyk D., Franceschini A., Wyder S., Forslund K., Heller D., Huerta-Cepas J., Simonovic M., Roth A., Santos A., Tsafou K. P., Kuhn M., Bork P., Jensen L. J., and von Mering C. (2015) STRING v10: protein-protein interaction networks, integrated over the tree of life. Nucleic Acids Res. 43, D447–D452 25352553 10.1093/nar/gku1003 PMC4383874

[26] Ruepp A., Brauner B., Dunger-Kaltenbach I., Frishman G., Montrone C., Stransky M., Waegele B., Schmidt T., Doudieu O. N., Stumpflen V., and Mewes H. W. (2008) CORUM: the comprehensive resource of mammalian protein complexes. Nucleic Acids Res. 36, D646–D650 17965090 10.1093/nar/gkm936 PMC2238909

[27] Nepusz T., Yu H., and Paccanaro A. (2012) Detecting overlapping protein complexes in protein-protein interaction networks. Nat. Methods 9, 471–472 22426491 10.1038/nmeth.1938 PMC3543700

[28] Li Z., Zou C. B., Yao Y., Hoyt M. A., McDonough S., Mackey Z. B., Coffino P., and Wang C. C. (2002) An easily dissociated 26 S proteasome catalyzes an essential ubiquitin-mediated protein degradation pathway in Trypanosoma brucei. J. Biol. Chem. 277, 15486–15498 11854272 10.1074/jbc.M109029200

[29] Aslett M., Aurrecoechea C., Berriman M., Brestelli J., Brunk B. P., Carrington M., Depledge D. P., Fischer S., Gajria B., Gao X., Gardner M. J., Gingle A., Grant G., Harb O. S., Heiges M., Hertz-Fowler C., Houston R., Innamorato F., Iodice J., Kissinger J. C., Kraemer E., Li W., Logan F. J., Miller J. A., Mitra S., Myler P. J., Nayak V., Pennington C., Phan I., Pinney D. F., Ramasamy G., Rogers M. B., Roos D. S., Ross C., Sivam D., Smith D. F., Srinivasamoorthy G., Stoeckert C. J. Jr, Subramanian S., Thibodeau R., Tivey A., Treatman C., Velarde G., and Wang H. (2010) TriTrypDB: a functional genomic resource for the Trypanosomatidae. Nucleic Acids Res. 38, D457–D462 19843604 10.1093/nar/gkp851 PMC2808979

[30] Vizcaino J. A., Csordas A., Del-Toro N., Dianes J. A., Griss J., Lavidas I., Mayer G., Perez-Riverol Y., Reisinger F., Ternent T., Xu Q. W., Wang R., and Hermjakob H. (2016) 2016 update of the PRIDE database and its related tools. Nucleic Acids Res. 44, 11033 27683222 10.1093/nar/gkw880 PMC5159556

[31] Zikova A., Schnaufer A., Dalley R. A., Panigrahi A. K., and Stuart K. D. (2009) The F(0)F(1)-ATP synthase complex contains novel subunits and is essential for procyclic Trypanosoma brucei. PLoS Pathogens 5, e1000436 19436713 10.1371/journal.ppat.1000436 PMC2674945

[32] Zikova A., Panigrahi A. K., Dalley R. A., Acestor N., Anupama A., Ogata Y., Myler P. J., and Stuart K. (2008) Trypanosoma brucei mitochondrial ribosomes: affinity purification and component identification by mass spectrometry. Mol. Cell. Proteomics 7, 1286–1296 18364347 10.1074/mcp.M700490-MCP200 PMC2493383

[33] Clemmens C. S., Morris M. T., Lyda T. A., Acosta-Serrano A., and Morris J. C. (2009) Trypanosoma brucei AMP-activated kinase subunit homologs influence surface molecule expression. Exp. Parasitol. 123, 250–257 19647733 10.1016/j.exppara.2009.07.010 PMC2774744

[34] Lueong S., Merce C., Fischer B., Hoheisel J. D., and Erben E. D. (2016) Gene expression regulatory networks in Trypanosoma brucei: insights into the role of the mRNA-binding proteome. Mol. Microbiol. 100, 457–471 26784394 10.1111/mmi.13328

[35] Schwede A., Ellis L., Luther J., Carrington M., Stoecklin G., and Clayton C. (2008) A role for Caf1 in mRNA deadenylation and decay in trypanosomes and human cells. Nucleic Acids Res. 36, 3374–3388 18442996 10.1093/nar/gkn108 PMC2425496

[36] Farber V., Erben E., Sharma S., Stoecklin G., and Clayton C. (2013) Trypanosome CNOT10 is essential for the integrity of the NOT deadenylase complex and for degradation of many mRNAs. Nucleic Acids Res. 41, 1211–1222 23221646 10.1093/nar/gks1133 PMC3553956

[37] Mauxion F., Preve B., and Seraphin B. (2013) C2ORF29/CNOT11 and CNOT10 form a new module of the CCR4-NOT complex. RNA Biol. 10, 267–276 23232451 10.4161/rna.23065 PMC3594285

[38] Dosil M., and Bustelo X. R. (2004) Functional characterization of Pwp2, a WD family protein essential for the assembly of the 90 S pre-ribosomal particle. J. Biol Chem. 279, 37385–37397 15231838 10.1074/jbc.M404909200

[39] Meier U. T. (2006) How a single protein complex accommodates many different H/ACA RNAs. Trends Biochem. Sci. 31, 311–315 16647858 10.1016/j.tibs.2006.04.002 PMC4314714

[40] Jones C., Anderson S., Singha U. K., and Chaudhuri M. (2008) Protein phosphatase 5 is required for Hsp90 function during proteotoxic stresses in Trypanosoma brucei. Parasitol. Res. 102, 835–844 18193284 10.1007/s00436-007-0817-z

[41] Folgueira C., and Requena J. M. (2007) A postgenomic view of the heat shock proteins in kinetoplastids. FEMS Microbiol. Rev. 31, 359–377 17459115 10.1111/j.1574-6976.2007.00069.x

[42] Zamudio J. R., Mittra B., Chattopadhyay A., Wohlschlegel J. A., Sturm N. R., and Campbell D. A. (2009) Trypanosoma brucei spliced leader RNA maturation by the cap 1 2′-O-ribose methyltransferase and SLA1 H/ACA snoRNA pseudouridine synthase complex. Mol. Cell. Biol. 29, 1202–1211 19103757 10.1128/MCB.01496-08 PMC2643836

[43] Lott K., Zhu L., Fisk J. C., Tomasello D. L., and Read L. K. (2014) Functional interplay between protein arginine methyltransferases in Trypanosoma brucei. MicrobiologyOpen 3, 595–609 25044453 10.1002/mbo3.191 PMC4234254

[44] Kafkova L., Debler E. W., Fisk J. C., Jain K., Clarke S. G., and Read L. K. (2017) The Major Protein Arginine Methyltransferase in Trypanosoma brucei Functions as an Enzyme-Prozyme Complex. J. Biol. Chem. 292, 2089–2100 27998975 10.1074/jbc.M116.757112 PMC5313084

[45] Johnson A. E., and van Waes M. A. (1999) The translocon: a dynamic gateway at the ER membrane. Ann. Rev. Cell Develop. Biol. 15, 799–842 10.1146/annurev.cellbio.15.1.799 10611978

[46] Ferguson M. A., Low M. G., and Cross G. A. (1985) Glycosyl-sn-1,2-dimyristylphosphatidylinositol is covalently linked to Trypanosoma brucei variant surface glycoprotein. J. Biol. Chem. 260, 14547–14555 4055788

[47] Gazestani V. H., Yip C. W., Nikpour N., Berghuis N., and Salavati R. (2017) TrypsNetDB: An integrated framework for the functional characterization of trypanosomatid proteins. PLoS Neglected Tropical Dis. 11, e0005368 10.1371/journal.pntd.0005368 PMC5310917 28158179

