# Supplementary material for: Prediction of Protein Complexes in Trypanosoma brucei by Protein Correlation Profiling Mass Spectrometry and Machine Learning
Source: Mol Cell Proteomics. 2017 Oct 17;16(12):2254–67. doi: 10.1074/mcp.O117.068122 (PMC5724185; doi:10.1074/mcp.O117.068122)
Supplement: Supplemental Data [file supp_16_12_2254__index.html]

Prediction of protein complexes in Trypanosoma brucei by protein correlation profiling mass spectrometry and machine learning. — Prediction of Protein Complexes in Trypanosoma brucei by Protein Correlation Profiling Mass Spectrometry and Machine Learning — Protein Complex Prediction in Trypanosoma brucei — Supplemental Data 

# Prediction of Protein Complexes in *Trypanosoma brucei* by Protein Correlation Profiling Mass Spectrometry and Machine Learning

## Supplemental Data

- Supplementary\_Table\_19 (.xlsx, 281 KB) - Supplemental Table 19
- Supplementary\_Table\_20 (.xlsx, 16 KB) - Supplemental Table 20
- Supplemental Data (.xlsx, 849 KB) - Supplemental Table 21
- Supplemental Tables, Figures and Legends (.pdf, 10.0 MB) - Supplemental Tables, Figures and Legends
- Supplementary Table 22 (.xlsx, 12.7 MB) - Supplementary Table 22
