# Supplementary material for: Prediction of Protein Complexes in Trypanosoma brucei by Protein Correlation Profiling Mass Spectrometry and Machine Learning
Source: Mol Cell Proteomics. 2017 Oct 17;16(12):2254–67. doi: 10.1074/mcp.O117.068122 (PMC5724185; doi:10.1074/mcp.O117.068122)
Supplement: Supplemental Data [file 10.1074_O117.068122_mcp.O117.068122-4.pdf]

# Supplementary Tables

**Supp Table 1. Gold standard protein complexes.**

List of protein complexes used as true positive interactors in random forest machine learning analysis. A representative protein complex name is shown along with the gene IDs of component proteins.

| Protein Complex | Gene IDs |
|---|---|
| Acidic Ribosomal | Tb927.10.3380; Tb927.9.5690 |
| ALBA | Tb927.4.2040; Tb927.11.4460; Tb927.4.2030; Tb927.11.4450 |
| Arginine N-methyltransferase | Tb927.1.4690; Tb927.10.3560 |
| ARP2/3 | Tb927.10.4540; Tb927.9.5350; Tb927.10.15800; Tb927.2.2900; Tb927.8.4410 |
| ATP synthase | Tb927.3.1380; Tb927.10.180; Tb927.5.1710; Tb927.6.4990; Tb927.10.5050; Tb927.7.7430 |
| Dihydrolipoamide dehydrogenase | Tb927.8.7380; Tb927.3.4390; Tb927.4.5040; Tb927.5.3800 |
| DNA polymerase | Tb927.11.8890; Tb11.v5.0480 |
| Exosome | Tb927.10.7450; Tb927.11.11030; Tb927.11.16600; Tb927.4.1630; Tb927.5.1200; Tb927.6.670; Tb927.9.7070 |
| Fibrillarin | Tb927.10.14750; Tb927.10.7500 |
| Nucleosome | Tb927.10.10590; Tb927.5.4260; Tb927.7.2940 |
| Mitochondrial peptidase | Tb927.11.3980; Tb927.5.1060 |
| Mitochondrial RNABP | Tb927.11.13280; Tb927.11.1710 |
| Mitochondrial SSU | Tb927.11.2530; Tb927.10.3580; Tb927.7.3050 |
| MRB1 complex | Tb927.2.3800; Tb927.7.2570 |
| MVP | Tb927.5.4460; Tb927.10.1990; Tb927.10.6310 |
| N-acetyltransferase | Tb927.10.5670; Tb927.11.4530; Tb927.10.3150 |
| Nucleosome assembly | Tb927.10.15180; Tb927.9.5730 |
| Oxoglutarate dehydrogenase | Tb927.11.1450; Tb927.11.9980; Tb927.11.11680 |
| Oxoisovalerate dehydrogenase | Tb927.10.4330; Tb927.10.660 |

| | |
|---|---|
| **Phosphoribosylpyrophosphate synthetase** | Tb927.5.2960; Tb927.11.3030; Tb927.10.9430; Tb927.5.3170 |
| **Prefoldin** | Tb927.7.570; Tb927.7.2590; Tb927.11.12680; Tb927.5.580; Tb927.6.2280; Tb927.11.800; Tb927.11.16040; Tb927.11.12910 |
| **Proteasome core** | Tb927.10.290; Tb927.7.4790; Tb927.9.11310; Tb927.4.430; Tb927.6.1260; Tb927.7.4420; Tb927.9.9670; Tb927.11.7020; Tb927.3.780; Tb927.10.4710; Tb927.10.230; Tb11.v5.0196; Tb927.10.6080; Tb927.11.7270 |
| **Proteasome regulatory** | Tb927.11.9220; Tb927.10.3520; Tb927.10.15720; Tb927.10.9740; Tb927.11.14430; Tb927.10.1550; Tb927.11.16030; Tb927.7.2550; Tb927.6.1090 |
| **Pyruvate dehydrogenase** | Tb927.10.12700; Tb927.3.1790; Tb927.10.7570 |
| **Ribosome 60S** | Tb927.9.3990; Tb927.8.1340; Tb927.3.3320; Tb927.7.1730 |
| **ruvB-like DNA helicase** | Tb927.4.1270; Tb927.4.200 |
| **SMD** | Tb927.2.4540; Tb927.2.5850; Tb927.4.890 |
| **Spliceosome** | Tb927.5.2290; Tb927.11.15430; Tb927.9.11110 |
| **Succinyl-CoA** | Tb927.3.2230; Tb927.10.2560 |
| **T-Complex** | Tb927.10.1060; Tb927.10.8190; Tb927.11.16760; Tb927.11.3240; Tb927.11.14250; Tb927.11.1900; Tb927.9.11270; Tb927.8.3150 |
| **Translation elongation** | Tb927.10.5840; Tb927.4.3590; Tb927.11.13190 |
| **tRNA processing complex** | Tb927.9.5210; Tb927.8.5330; Tb927.10.1250; Tb927.10.10030 |

**Supp Table 2. Machine learning protein complex predictions.**

Protein complex numbers match those on 'Complex Explorer' web visualisation tool.

| Predicted Protein Complex | Protein Id | Protein Description |
|---|---|---|
| **1** | Tb927.10.2290 | chaperone protein DNAj, putative |
| | Tb927.11.7380 | glycerol-3-phosphate dehydrogenase (FAD-dependent), mitochondrial |
| | Tb927.8.6080 | Glycerophosphoryl diester phosphodiesterase family, putative (POMP42) |
| | Tb927.9.15000 | proteasome complex subunit Rpn13 ubiquitin receptor, putative |
| **2** | Tb927.4.1330 | DNA topoisomerase IB, large subunit |
| | Tb927.9.10530 | hypothetical protein, conserved |
| **3** | Tb927.10.3700 | AMP-activated protein kinase, gamma regulatory subunit, SNF1-related protein kinase regulatory subunit gamma, AMPK subunit gamma (AMPKG) |
| | Tb927.10.5310 | SNF1-related protein kinases, putative |

| | | |
|---|---|---|
| | Tb927.3.4560 | 5'-AMP-activated protein kinase catalytic subunit alpha, putative, AMPK subunit alpha, putative, SNF1-related protein kinase catalytic subunit alpha, putative |
| | Tb927.8.2450 | SNF1-related protein kinase regulatory subunit beta, 5'-AMP-activated protein kinase subunit beta, AMPK subunit beta (AMPKB) |
| | Tb927.9.9270 | hypothetical protein, conserved |
| **4** | Tb927.11.12930 | DEAD-box helicase, putative |
| | Tb927.11.5990 | hypothetical protein, conserved |
| | Tb927.4.1070 | 50S ribosomal protein L13, putative |
| | Tb927.6.4080 | hypothetical protein, conserved |
| | Tb927.7.1640 | ras-like small GTPase, putative (TbEAR) |
| | Tb927.7.3460 | hypothetical protein, conserved |
| **5** | Tb927.11.11290 | heat shock protein 70, putative |
| | Tb927.11.7150 | NGG1 interacting factor 3-like |
| | Tb927.5.3520 | queuine tRNA-ribosyltransferase, putative |
| | Tb927.6.4920 | S-adenosylmethionine synthetase, putative (METK1) |
| | Tb927.9.5190 | proliferative cell nuclear antigen (PCNA), putative |
| | Tb927.9.9820 | glyceraldehyde-3-phosphate dehydrogenase, putative |
| **6** | Tb11.1390 | hypothetical protein, conserved |
| | Tb927.1.4050 | protein phosphatase with EF-Hand domains (PPEF), ser/thr protein phosphatase, putative |
| | Tb927.10.12500 | P-type H+-ATPase, putative |
| | Tb927.10.12510 | P-type H+-ATPase, putative |
| | Tb927.10.7700 | ABC transporter, putative |
| | Tb927.10.8530 | glucose transporter 2A (THT2A) |
| | Tb927.10.9080 | pteridine transporter, putative |
| | Tb927.11.540 | ABC transporter, mitochondrial, putative, multidrug resistance protein, mitochondrial, putative (ABCT) |
| | Tb927.11.5970 | phosphoinositide-specific phospholipase C, putative |
| | Tb927.11.6040 | Nodulin-like, putative |
| | Tb927.4.4360 | monoglyceride lipase, putative |
| | Tb927.4.4490 | multidrug resistance protein E, p-glycoprotein (MRPE) |
| | Tb927.8.2160 | multidrug resistance protein A, p-glycoprotein (PGPA) |
| | Tb927.9.15460 | calcium motive p-type ATPase, putative |
| | Tb927.9.6310 | ABC transporter, putative |
| **7** | Tb11.v5.0807 | ribonuclease, putative |
| | Tb927.10.5620 | fructose-bisphosphate aldolase, glycosomal (ALD) |
| | Tb927.11.6830 | Domain of unknown function(DUF2779), putative |
| | Tb927.11.7310 | RNA binding protein, putative |
| | Tb927.2.820 | retrotransposon hot spot protein (RHS, pseudogene), putative, retrotransposon hot spot protein 1 (RHS1), interrupted |
| | Tb927.9.9580 | tubulin tyrosine ligase protein, putative |
| **8** | Tb927.10.11530 | hypothetical protein, conserved |
| | Tb927.11.10300 | hypothetical protein, conserved |

| | | |
|---|---|---|
| | Tb927.11.5510 | dynein light chain p28, axonemal, putative |
| | Tb927.3.5580 | tryptophanyl-tRNA synthetase |
| | Tb927.7.4570 | inosine-guanine nucleoside hydrolase (IG-NH) |
| | Tb927.9.12290 | Peroxin 19 |
| **9** | Tb927.1.870 | deoxyhypusine synthase, putative |
| | Tb927.10.2750 | deoxyhypusine synthase, putative |
| **10** | Tb927.11.10240 | hslVU complex proteolytic subunit, threonine peptidase, Clan T(1), family T1B, ATP-dependent protease subunit HslV (HsIV) |
| | Tb927.11.2230 | carnitine O-acetyltransferase, putative (CAT) |
| | Tb927.4.1270 | ruvB-like DNA helicase, putative |
| | Tb927.4.2000 | ruvB-like DNA helicase, putative (RUVBL) |
| | Tb927.6.3610 | SET domain containing protein, putative |
| | Tb927.6.950 | cysteinyl-tRNA synthetase, putative |
| | Tb927.7.5890 | hypothetical protein, conserved |
| | Tb927.9.5900 | glutamate dehydrogenase (GDH) |
| **11** | Tb927.1.1670 | ARM-like helical domain-containing protein |
| | Tb927.4.1890 | hypothetical protein, conserved |
| | Tb927.5.1090 | threonyl-tRNA synthetase, putative |
| | Tb927.7.760 | hypothetical protein, conserved |
| **12** | Tb927.10.13670 | serine/threonine protein phosphatase 5 |
| | Tb927.10.14030 | hypothetical protein, conserved |
| | Tb927.3.3580 | heat shock protein 90, putative (LPG3) |
| | Tb927.5.3260 | WD domain, G-beta repeat, putative |
| | Tb927.9.9860 | Hsp70 protein, putative |
| **13** | Tb927.5.2250 | META domain/Domain of unknown function (DUF1935), putative |
| | Tb927.6.4950 | mago nashi-like protein, putative |
| | Tb927.7.1170 | RNA-binding protein, putative (Y14) |
| **14** | Tb927.11.15370 | hypothetical protein, conserved (TbKap123) |
| | Tb927.3.1670 | hypothetical protein, conserved |
| | Tb927.3.4250 | hypothetical protein, conserved |
| **15** | Tb927.10.10030 | hypothetical protein, conserved |
| | Tb927.10.1250 | Aminoacyl-tRNA editing domain containing protein, putative |
| | Tb927.5.4450 | hypothetical protein, conserved |
| | Tb927.8.5330 | tyrosyl/methionyl-tRNA synthetase, putative |
| | Tb927.9.5210 | glutaminyl-tRNA synthetase, putative |
| **16** | Tb927.10.12700 | pyruvate dehydrogenase E1 alpha subunit, putative |
| | Tb927.10.7570 | dihydrolipoamide acetyltransferase E2 subunit, putative |
| | Tb927.3.1790 | pyruvate dehydrogenase E1 beta subunit, putative |
| | Tb927.5.1090 | threonyl-tRNA synthetase, putative |
| **17** | Tb927.1.1670 | ARM-like helical domain-containing protein |
| | Tb927.10.15180 | nucleosome assembly protein, putative |
| | Tb927.11.6370 | leucine-rich repeat protein (LRRP), putative |

| | | |
|---|---|---|
| | Tb927.9.5730 | nucleosome assembly protein-like protein |
| **18** | Tb927.10.2620 | CS domain containing protein, putative |
| | Tb927.2.1890 | E2-like ubiquitin-conjugation enzyme (ATG3) |
| **19** | Tb927.10.15170 | hypothetical protein, conserved |
| | Tb927.10.1990 | major vault protein, putative (MVP) |
| | Tb927.10.6310 | major vault protein, putative (MVP) |
| | Tb927.5.4460 | major vault protein, putative (MVP) |
| **20** | Tb927.8.5900 | Sedlin, N-terminal conserved region, putative |
| | Tb927.9.12150 | transport protein particle (TRAPP) subunit, putative |
| **21** | Tb927.10.3710 | proteasome activator protein PA26 (pa26) |
| | Tb927.3.4040 | Ankyrin repeats (3 copies), putative |
| **22** | Tb927.10.11390 | 60S ribosomal protein L6, putative |
| | Tb927.3.3320 | 60S ribosomal protein L13, putative |
| | Tb927.4.2180 | 60S ribosomal protein L35a, putative |
| | Tb927.6.720 | 40S ribosomal protein L14, putative |
| | Tb927.7.1730 | 60S ribosomal protein L7, putative |
| | Tb927.8.1340 | 60S ribosomal protein L7a, putative |
| | Tb927.9.3990 | ribosomal protein S7, putative |
| **23** | Tb927.11.9710 | 60S ribosomal protein L10a, putative (RPL10A) |
| | Tb927.5.2770 | SET domain containing protein, putative |
| **24** | Tb927.10.10960 | heat shock protein, putative |
| | Tb927.8.3100 | coronin, putative (CRN12) |
| **25** | Tb927.10.1100 | 60S ribosomal protein L9, putative |
| | Tb927.11.14130 | ribosomal protein L18, putative |
| | Tb927.11.3600 | 40S ribosomal protein S4, putative |
| | Tb927.11.9730 | 60S ribosomal protein L34, putative |
| | Tb927.4.1100 | ribosomal protein L21E (60S), putative |
| | Tb927.4.1860 | ribosomal protein S19, putative |
| | Tb927.5.1610 | 60S ribosomal protein L13a, putative |
| | Tb927.7.1050 | 40S ribosomal protein S16, putative |
| | Tb927.7.5020 | 60S ribosomal protein L19, putative |
| | Tb927.8.6160 | 40S ribosomal protein S8, putative |
| | Tb927.9.11410 | 60S ribosomal protein L23, putative |
| | Tb927.9.11490 | 60S ribosomal protein L27a, 60S ribosomal protein L28, 60S ribosomal protein L29 (RPL27A) |
| | Tb927.9.8420 | 60S ribosomal protein L10, putative, QM-like protein |
| **26** | Tb927.10.3760 | vacuolar ATP synthase subunit d, putative |
| | Tb927.10.730 | ATP synthase, putative |
| | Tb927.11.11690 | Vacuolar proton pump subunit B, putative, V-type proton ATPase subunit B, putative |
| | Tb927.11.9420 | ATP synthase, putative |
| **27** | Tb927.10.9020 | Gcd10p family, putative |
| | Tb927.11.11660 | conserved protein |

| | | |
|---|---|---|
| | Tb927.11.9210 | NOL1/NOP2/sun family, putative |
| | Tb927.2.380 | retrotransposon hot spot protein 2 (RHS2), putative |
| | Tb927.2.830 | retrotransposon hot spot protein (RHS, pseudogene), putative, retrotransposon hot spot protein 1 (RHS1), interrupted |
| **28** | Tb927.1.2580 | RNasePH-like protein, exosome-associated protein 1 (EAP1) |
| | Tb927.10.5840 | translation elongation factor 1-beta, putative |
| | Tb927.10.7450 | exosome complex exonuclease RRP41A, Ribosomal RNA processing protein 41A (RRP41A) |
| | Tb927.11.16600 | exosome-associated protein 2 (EAP2) |
| | Tb927.11.630 | RNA polymerase I second largest subunit (RPA135) |
| | Tb927.3.1150 | Conserved hypothetical ATP binding protein, putative |
| | Tb927.3.1300 | hypothetical protein, conserved |
| | Tb927.5.1200 | exosome component CSL4 (CSL4) |
| | Tb927.6.670 | ribosomal RNA processing protein 45, exosome complex exonuclease (RRP45) |
| | Tb927.7.4670 | ribosomal RNA processing protein 4, exosome complex exonuclease (RRP4) |
| | Tb927.7.710 | heat shock 70 kDa protein, putative (HSP70) |
| | Tb927.9.7070 | exosome complex exonuclease RRP40 (RRP40) |
| **29** | Tb927.11.12680 | prefoldin subunit 2, putative |
| | Tb927.5.580 | prefoldin subunit, putative |
| | Tb927.7.2590 | prefoldin, putative |
| | Tb927.7.570 | prefoldin, putative |
| **30** | Tb927.10.1550 | proteasome regulatory non-ATP-ase subunit 5 (RPN5) |
| | Tb927.10.15720 | proteasome regulatory non-ATP-ase subunit 9 (RPN9) |
| | Tb927.10.3030 | proteasome regulatory non-ATPase subunit 11 (RPN11) |
| | Tb927.10.3520 | protease regulatory ATPase subunit 4 (RPT4) |
| | Tb927.10.9740 | Regulatory particle triple-A ATPase subunit 6, 19S proteasome regulatory subunit (RPT6) |
| | Tb927.11.14430 | proteasome regulatory non-ATP-ase subunit |
| | Tb927.11.16030 | proteasome regulatory non-ATP-ase subunit 7 (RPN7) |
| | Tb927.11.3740 | proteasome regulatory ATPase subunit 2 (RPT2) |
| | Tb927.11.8310 | class I transcription factor A, subunit 4 (CITFA-4) |
| | Tb927.2.2440 | proteasome regulatory non-ATPase subunit 6 (RPN6) |
| | Tb927.6.1090 | proteasome regulatory ATPase subunit 3 (RPT3) |
| | Tb927.7.2500 | proteasome regulatory ATPase subunit 1 |
| | Tb927.7.2550 | proteasome regulatory ATPase subunit 5 (RPT5) |
| | Tb927.8.570 | proteasome regulatory non-ATP-ase subunit 10 |
| **31** | Tb11.v5.0196 | Proteasome subunit A N-terminal signature/Proteasome subunit, putative |
| | Tb927.10.230 | proteasome subunit alpha type-5, putative |
| | Tb927.10.290 | proteasome alpha 2 subunit, putative |
| | Tb927.10.4710 | 20S proteasome subunit, proteasome subunit beta type-2, putative (PSB4) |
| | Tb927.10.6080 | proteasome subunit beta type-5, putative, proteasome subunit beta type-5, putative |

| | | |
|---|---|---|
| | Tb927.11.7020 | proteasome alpha 7 subunit, putative (PSA4) |
| | Tb927.11.7270 | proteasome beta 3 subunit, putative (PSB3) |
| | Tb927.3.780 | proteasome alpha 7 subunit (TbPSA7) |
| | Tb927.4.430 | proteasome beta 7 subunit |
| | Tb927.6.1260 | proteasome beta-1 subunit, putative (PSB1) |
| | Tb927.7.4420 | proteasome alpha 3 subunit, putative |
| | Tb927.7.4790 | proteasome beta 6 subunit, 20S proteasome beta 6 subunit, putative (BETA6) |
| | Tb927.9.11310 | unspecified product |
| | Tb927.9.9670 | proteasome alpha 1 subunit, putative, 20S proteasome subunit alpha-6, (putative) (TbPSA6) |
| **32** | Tb927.10.3280 | 60S ribosomal proteins L38, putative |
| | Tb927.2.6090 | 60S ribosomal protein L44 (RPL44) |
| **33** | Tb927.10.3380 | 60S acidic ribosomal protein P2, putative |
| | Tb927.9.5690 | 60S acidic ribosomal protein, putative |
| **34** | Tb927.10.2200 | hypothetical protein, conserved |
| | Tb927.10.4220 | hypothetical protein, conserved |
| **35** | Tb11.02.5380b | exosome complex exonuclease RRP44p homologue, putative |
| | Tb927.10.7630 | transportin2- like protein |
| **36** | Tb927.10.9250 | adenylyl cyclase-associated protein, putative |
| | Tb927.7.880 | RNA-binding protein, putative (RPB25) |
| **37** | Tb927.11.14190 | Staphylococcal nuclease homologue/Tudor domain containing protein, putative |
| | Tb927.11.2650 | heat shock protein 84, putative |
| **38** | Tb927.11.11680 | 2-oxoglutarate dehydrogenase E2 component, putative |
| | Tb927.11.1450 | 2-oxoglutarate dehydrogenase E1 component, putative |
| | Tb927.11.9980 | 2-oxoglutarate dehydrogenase E1 component, putative |
| **39** | Tb927.11.3660 | Dynein light chain Tctex-type, putative |
| | Tb927.7.4820 | Trm112p-like protein, putative |
| **40** | Tb927.11.3980 | mitochondrial processing peptidase alpha subunit, putative, metallo-peptidase, Clan ME, Family M16 |
| | Tb927.5.1060 | mitochondrial processing peptidase, beta subunit, putative, metallo-peptidase, Clan ME, Family M16 |
| **41** | Tb927.11.4360 | Protein of unknown function (DUF1014), putative |
| | Tb927.3.1920 | NOT5 protein (NOT5) |
| **42** | Tb927.11.4480 | radial spoke protein RSP4/6, putative |
| | Tb927.3.2890 | radial spoke protein RSP10, putative |
| **43** | Tb927.11.4920 | hypothetical protein, conserved |
| | Tb927.5.1900 | hypothetical protein, conserved |
| **44** | Tb927.11.9610 | eukaryotic translation initiation factor 3 subunit 2, putative (eIF-3 beta) |
| | Tb927.5.2570 | translation initiation factor, putative (EIF3B) |
| **45** | Tb927.2.2230 | hypothetical protein, conserved |
| | Tb927.2.2390 | hypothetical protein, conserved |
| **46** | Tb927.10.1890 | cysteine peptidase, Clan CA, family C2, putative |

| | | |
|---|---|---|
| | Tb927.2.5810 | Holliday-junction resolvase-like of SPT6/SH2 domain containing protein, putative |
| **47** | Tb927.3.1210 | protein transport protein Sec24 (SEC24.1) |
| | Tb927.3.3890 | hypothetical protein, conserved |
| **48** | Tb927.3.2660 | TatD related DNase, putative |
| | Tb927.9.8200 | Pescadillo N-terminus/BRCA1 C Terminus (BRCT) domain containing protein, putative |
| **49** | Tb927.3.3630 | Elongation factor Ts, mitochondrial, putative (EF-Ts) |
| | Tb927.7.1340 | 10 kDa heat shock protein, putative (HSP10) |
| **50** | Tb927.3.5310 | paraflagellar rod protein |
| | Tb927.5.940 | NADH-dependent fumarate reductase, putative |
| **51** | Tb927.3.5370 | hypothetical protein, conserved |
| | Tb927.6.1870 | eukaryotic translation initiation factor 4e, putative |
| **52** | Tb927.11.3850 | AMP deaminase, putative |
| | Tb927.4.1680 | ZFP family member, putative (ZC3H10) |
| **53** | Tb927.4.2630 | ATP-dependent DEAD/H RNA helicase, putative |
| | Tb927.4.4160 | mitochondrial RNA binding protein (MRB4160) |
| **54** | Tb927.6.1200 | hypothetical protein, conserved |
| | Tb927.7.930 | zinc finger CCCH domain containing protein 17 (ZC3H17) |
| **55** | Tb927.5.3900 | Galactose oxidase, central domain/Domain of unknown function (DUF4110), putative |
| | Tb927.6.1990 | hypothetical protein, conserved |
| **56** | Tb927.11.4160 | predicted C2 domain protein |
| | Tb927.6.3310 | calpain-like cysteine peptidase, putative, cysteine peptidase, Clan CA, family C2, putative |
| **57** | Tb927.6.2170 | co-chaperone GrpE, putative |
| | Tb927.6.4000 | small glutamine-rich tetratricopeptide repeat protein, putative, (SGT) |
| **58** | Tb927.10.600 | hypothetical protein, conserved |
| | Tb927.6.4200 | hypothetical protein, conserved |
| **59** | Tb927.11.2640 | ras-like small GTPase, putative (TbNST) |
| | Tb927.6.4750 | hypothetical protein, conserved |
| **60** | Tb927.7.1360 | hypothetical protein, conserved |
| | Tb927.7.7140 | Vta1 like, putative |
| **61** | Tb927.11.10330 | Regulator of chromosome condensation (RCC1) repeat, putative |
| | Tb927.7.970 | NMD3 family, putative |
| **62** | Tb927.8.1420 | acyl-CoA dehydrogenase, mitochondrial precursor, putative |
| | Tb927.8.1740 | hypothetical protein, conserved |
| **63** | Tb927.11.10760 | kinesin-like protein, putative |
| | Tb927.8.2630 | kinesin, putative |
| **64** | Tb927.8.4400 | hypothetical protein, conserved |
| | Tb927.9.8820 | hypothetical protein, conserved |
| **65** | Tb927.8.1090 | NPAPL (NPAPL) |
| | Tb927.8.4870 | DIGIT |
| **66** | Tb927.10.15410 | glycosomal malate dehydrogenase (gMDH) |

| | | |
|---|---|---|
| | Tb927.9.11940 | replication factor A protein 3, putative |
| **67** | Tb927.7.4500 | PX domain containing protein, putative |
| | Tb927.9.13380 | phosphoinositide-binding protein, putative |
| **68** | Tb927.9.5040 | cAMP-specific phosphodiesterase (PDEB1) |
| | Tb927.9.5100 | cAMP-specific phosphodiesterase (PDEB2) |
| **69** | Tb927.11.1840 | hypothetical protein, conserved |
| | Tb927.6.5070 | hypothetical protein, conserved |
| **70** | Tb927.10.8940 | flagellum targeting protein kharon1, putative (KH1) |
| | Tb927.3.3750 | paraflagellar rod component, putative (PFC7) |
| **71** | Tb927.10.4330 | 2-oxoisovalerate dehydrogenase beta subunit, mitochondrial precursor, putative |
| | Tb927.10.660 | 2-oxoisovalerate dehydrogenase alpha subunit, putative |
| **72** | Tb927.11.6740 | pumilio/PUF RNA binding protein 10, putative |
| | Tb927.7.2170 | hypothetical protein, conserved |
| **73** | Tb927.1.1000 | developmentally regulated phosphoprotein |
| | Tb927.11.4780 | pyruvate dehydrogenase (lipoamide) kinase, putative |
| **74** | Tb927.10.15520 | signal recognition particle protein, putative |
| | Tb927.4.1850 | hypothetical protein, conserved |
| | Tb927.5.3800 | glutamine hydrolysing (not ammonia-dependent) carbomoyl phosphate synthase, putative |
| **75** | Tb927.10.14150 | nuclear segregation protein, putative |
| | Tb927.11.6440 | hypothetical protein, conserved |
| | Tb927.6.4770 | protein mkt1, putative (MKT1) |
| **76** | Tb927.10.170 | pseudouridine synthase, Cbf5p |
| | Tb927.4.470 | snoRNP protein GAR1, putative |
| | Tb927.4.750 | 50S ribosomal protein L7Ae, putative |
| **77** | Tb927.10.2720 | hypothetical protein, conserved |
| | Tb927.10.6680 | member of the NOL1/NOP2/sun family of proteins |
| | Tb927.10.9050 | pseudouridine synthase TruD, putative, tRNA pseudouridine synthase TruD, putative (pus7) |
| **78** | Tb927.11.13740 | receptor-type adenylate cyclase GRESAG 4, putative |
| | Tb927.11.17040 | expression site-associated gene 4 (ESAG4) protein, putative, receptor-type adenylate cyclase, putative |
| | Tb927.7.7470 | receptor-type adenylate cyclase GRESAG 4, putative |
| **79** | Tb927.10.3150 | N-acetyltransferase, putative |
| | Tb927.10.5670 | N-acetyltransferase subunit Nat1, putative (NAT1) |
| | Tb927.11.4530 | N-acetyltransferase subunit ARD1 (ARD1) |
| **80** | Tb927.11.11330 | heat shock protein 70 |
| | Tb927.11.7510 | glucose-regulated protein 78, putative, luminal binding protein 1 (BiP), putative (BiP) |
| | Tb927.6.3800 | heat shock 70 kDa protein, mitochondrial precursor, putative |
| **81** | Tb927.2.4540 | Small nuclear ribonucleoprotein-associated protein B (snRNP-B) (Sm protein B) (Sm-B) (SmB), putative (TbSmB) |
| | Tb927.2.5850 | small nuclear ribonucleoprotein SmD2 (Sm-D2) |
| | Tb927.4.890 | small nuclear ribonucleoprotein SmD3, putative (SmD3) |

| | | |
|---|---|---|
| **82** | Tb927.3.2280 | vacuolar sorting protein 33 , putative |
| | Tb927.8.3370 | Ran-binding protein, putative |
| | Tb927.8.6270 | hypothetical protein, conserved |
| **83** | Tb927.11.5840 | Protein translation factor SUI1 homolog, putative |
| | Tb927.3.3300 | hypothetical protein, conserved |
| | Tb927.8.6240 | STOP axonemal protein |
| **84** | Tb927.11.4460 | ALBA-Domain Protein (ALBA1) |
| | Tb927.4.2030 | ALBA-Domain Protein (ALBA4) |
| | Tb927.4.2040 | ALBA-Domain Protein (ALBA3) |
| **85** | Tb927.10.8030 | hypothetical protein, conserved |
| | Tb927.11.6250 | hypothetical protein, conserved |
| | Tb927.5.1780 | hypothetical protein, conserved |
| **86** | Tb927.11.13960 | U6 snRNA-associated Sm-like protein LSm4p (TbLSm4) |
| | Tb927.5.4030 | U6 snRNA-associated Sm-like protein LSm7p (TbLSm7) |
| | Tb927.8.2850 | Poly(A)-specific ribonuclease PARN-1 |
| **87** | Tb927.10.10590 | histone H2B, putative |
| | Tb927.5.4260 | histone H4, putative |
| | Tb927.7.2940 | histone H2A, putative |
| **88** | Tb927.10.10010 | 60S acidic ribosomal protein, putative |
| | Tb927.10.1900 | DNA topoisomerase IA, putative |
| | Tb927.5.4420 | nucleolar RNA helicase II, putative, nucleolar RNA helicase Gu, putative |
| **89** | Tb927.7.6280 | Domain of unknown function (DUF3508), putative |
| | Tb927.8.1560 | hypothetical protein, conserved |
| **90** | Tb11.v5.0394 | hypothetical protein, conserved |
| | Tb927.6.2010 | AMP-binding enzyme, putative |
| | Tb927.6.740 | ATP-dependent DEAH-box RNA helicase, putative |
| **91** | Tb927.3.1010 | hypothetical protein, conserved |
| | Tb927.7.2640 | hypothetical protein, conserved |
| | Tb927.9.10400 | hypothetical protein, conserved |
| **92** | Tb927.10.3580 | hypothetical protein, conserved |
| | Tb927.11.2530 | Mitochondrial SSU ribosomal protein, putative, mitochondrial RNA binding complex 1 subunit |
| | Tb927.7.3050 | hypothetical protein, conserved |
| **93** | Tb927.11.1680 | vesicular-fusion protein SEC18, putative |
| | Tb927.7.1100 | hypothetical protein, conserved |
| | Tb927.7.3810 | 'Cold-shock' DNA-binding domain containing protein, putative |
| **94** | Tb927.2.4230 | NUP-1 protein, putative |
| | Tb927.7.3330 | hypothetical protein, conserved |
| | Tb927.8.3870 | SRP40, C-terminal domain containing protein, putative |
| **95** | Tb927.11.3830 | hypothetical protein, conserved |
| | Tb927.8.5090 | DNA-directed RNA polymerase I largest subunit (RPA190) |
| | Tb927.9.2120 | hypothetical protein, conserved |

| | | |
|---|---|---|
| **96** | Tb927.10.7230 | hypothetical protein, conserved |
| | Tb927.3.3520 | hypothetical protein, conserved (POMP25) |
| | Tb927.9.3400 | endo-beta-N-acetylglucosaminidase, putative |
| **97** | Tb927.11.16200 | cytoskeleton-associated protein 17, corset-associated protein 17 (CAP17) |
| | Tb927.8.6440 | RNA-binding protein, putative (RPB20) |
| | Tb927.9.9060 | Lsm12 protein, putative |
| **98** | Tb927.3.1600 | Tim10/DDP family zinc finger, putative |
| | Tb927.9.1350 | hypothetical protein, conserved |
| **99** | Tb927.10.5840 | translation elongation factor 1-beta, putative |
| | Tb927.10.7940 | methyltransferase, putative |
| | Tb927.11.13190 | elongation factor 1 gamma, putative |
| | Tb927.11.16490 | hypothetical protein, conserved |
| | Tb927.4.3590 | translation elongation factor 1-beta, putative |
| **100** | Tb927.11.12670 | Methyltransferase TYW3, putative |
| | Tb927.11.7080 | acidocalcisomal pyrophosphatase |
| | Tb927.8.2050 | GDP-mannose pyrophosphorylase |
| | Tb927.9.1880 | WD domain, G-beta repeat, putative |
| **101** | Tb927.1.1030 | leucine-rich repeat protein (LRRP), putative |
| | Tb927.10.2570 | lysosomal alpha-mannosidase precursor, putative |
| | Tb927.11.6670 | hypothetical protein, conserved |
| | Tb927.4.1520 | Pestivirus Npro endopeptidase C53, putative |
| **102** | Tb927.11.1050 | Ribosome production factor 1, putative |
| | Tb927.11.3120 | nucleolar GTP-binding protein 1 (NOG1) |
| | Tb927.11.6790 | predicted WD40 repeat protein |
| | Tb927.6.3790 | valosin-containing protein homolog, putative, AAA ATPase |
| **103** | Tb927.10.11170 | hypothetical protein, conserved |
| | Tb927.11.10840 | hypothetical protein, conserved |
| | Tb927.11.7770 | oxidoreductase-like protein |
| | Tb927.9.12780 | Leucine carboxyl methyltransferase/Cupin-like domain containing protein, putative |
| **104** | Tb927.5.1840 | hypothetical protein, conserved |
| | Tb927.8.6900 | transport protein particle (TRAPP) component, putative |
| **105** | Tb927.10.6120 | Peptidase M76 family, putative |
| | Tb927.3.2300 | DNL zinc finger, putative |
| | Tb927.6.2990 | Putative papain-like cysteine peptidase (DUF1796), putative |
| **106** | Tb927.4.360 | 1,2-Dihydroxy-3-keto-5-methylthiopentene dioxygenase, putative |
| | Tb927.6.3600 | hypothetical protein, conserved |
| **107** | Tb927.6.4530 | RNA-binding protein, putative (RBP17) |
| | Tb927.7.5760 | nuclear transport factor 2 protein, putative, mRNA transport regulator MTR2, putative, nuclear transport factor, nuclear transport factor 2 protein, putative (MTR2) |
| | Tb927.9.9450 | zinc finger protein family member, putative (ZC3H28) |
| **108** | Tb927.10.13270 | Periodic tryptophan protein 2 homolog, putative |

| | | |
|---|---|---|
| | Tb927.11.10480 | PQQ-like domain/WD domain, G-beta repeat/Utp21 specific WD40 associated putative domain containing protein, putative |
| | Tb927.11.460 | predicted WD40 repeat protein |
| | Tb927.7.4220 | WD domain, G-beta repeat/Dip2/Utp12 Family, putative |
| **109** | Tb927.10.13720 | RNA-binding protein 29, putative (RBP29) |
| | Tb927.11.10540 | hypothetical protein, conserved |
| **110** | Tb927.3.2490 | hypothetical protein, conserved |
| | Tb927.6.1770 | kinesin, putative |
| | Tb927.9.15470 | kinesin, putative |
| | Tb927.9.9730 | hypothetical protein, conserved |
| **111** | Tb927.6.4300 | glyceraldehyde 3-phosphate dehydrogenase, glycosomal (GAPDH) |
| | Tb927.8.2000 | cyclophilin, putative (NCP1) |
| | Tb927.8.3530 | glycerol-3-phosphate dehydrogenase [NAD+], glycosomal |
| | Tb927.9.8720 | fructose-1,6-bisphosphatase (FBPase) |
| **112** | Tb927.10.15800 | actin related protein 2, putative |
| | Tb927.10.4540 | ARP2/3 complex subunit, putative |
| | Tb927.2.2900 | ARP2/3 complex subunit, putative |
| | Tb927.8.4410 | ARP2/3 complex subunit, putative |
| | Tb927.9.5350 | actin related protein 3, putative |
| **113** | Tb927.11.6630 | 3-methylcrotonoyl-CoA carboxylase beta subunit, putative |
| | Tb927.3.4390 | dihydrolipoamide dehydrogenase, putative (GCVL-1) |
| | Tb927.4.5040 | dihydrolipoamide dehydrogenase, putative |
| | Tb927.8.6970 | 3-methylcrotonyl-CoA carboxylase alpha subunit, putative |
| | Tb927.8.7380 | dihydrolipoamide dehydrogenase, point mutation, acetoin dehydrogenase e3 component, putative |
| **114** | Tb927.1.3180 | 40S ribosomal protein S11, putative |
| | Tb927.10.14930 | Zinc finger CCCH domain-containing protein 39 (ZC3H39) |
| | Tb927.11.6300 | 40S ribosomal protein S5, putative |
| | Tb927.7.4910 | hypothetical protein, conserved |
| **115** | Tb927.11.8030 | hypothetical protein, conserved |
| | Tb927.3.3030 | START domain containing protein, putative |
| | Tb927.5.780 | hypothetical protein, conserved |
| | Tb927.7.3370 | hypothetical protein, conserved |
| **116** | Tb927.5.2320 | Mak10 subunit, NatC N(alpha)-terminal acetyltransferase, putative |
| | Tb927.7.2080 | methyltransferase, putative, mRNA cap methyltransferase-like protein |
| | Tb927.7.2360 | N-acetyltransferase, putative |
| | Tb927.8.960 | hypothetical protein, conserved |
| | Tb927.9.4500 | heat shock protein, putative, HSP70-like protein |
| **117** | Tb927.11.11080 | Nucleoporin (TbNup149) |
| | Tb927.11.6170 | protein transport protein SEC31, putative |
| | Tb927.11.7900 | mitochondrial RNA binding protein 16 (RBP16) |
| | Tb927.7.320 | hypothetical protein, conserved (TbRBP8) |
| | Tb927.9.3760 | poly(A) export protein, putative (TbGLE2) |

| | | |
|---|---|---|
| **118** | Tb927.11.8270 | hypothetical protein, conserved |
| | Tb927.5.2530 | Domain of unknown function (DUF1726)/Helicase/GNAT acetyltransferase 2/Possible tRNA binding domain containing protein, putative |
| | Tb927.8.1980 | UTP15 C terminal, putative |
| **119** | Tb927.5.4270 | ATP-dependent DEAD/H RNA helicase, putative |
| | Tb927.8.4820 | eukaryotic translation initation factor 4 gamma, putative (eIF4G3) |
| **120** | Tb927.10.2770 | eukaryotic translation initiation factor 5, putative |
| | Tb927.11.11010 | hypothetical protein, conserved |
| **121** | Tb927.10.11210 | hypothetical protein, conserved |
| | Tb927.11.12230 | ATP-dependent protease ATPase subunit HslU2 (HslU2) |
| | Tb927.2.3800 | MRB1-associated protein, guide RNA associated protein 1 (GAP1) |
| | Tb927.3.2900 | eukaryotic initiation factor 2a, putative |
| | Tb927.7.2570 | guide RNA associated protein, GAP2, mitochondrial RNA binding protein 1 |
| **122** | Tb927.10.14700 | hypothetical protein, conserved |
| | Tb927.3.3610 | peroxisomal targeting signal 2 receptor, putative, Peroxin-7, putative, PTS2 receptor, putative (PEX7) |
| | Tb927.8.5780 | phosphatase of regenerating liver-type phosphatase, putative |
| **123** | Tb927.10.5860 | hypothetical protein, conserved |
| | Tb927.11.16220 | hypothetical protein, conserved |
| | Tb927.4.1080 | V-type ATPase, A subunit, putative |
| **124** | Tb927.11.8100 | Ankyrin repeats (3 copies), putative |
| | Tb927.5.1280 | alanine racemase, putative |
| **125** | Tb927.11.14490 | RNA polymerase subunit, putative (RPB7) |
| | Tb927.8.6580 | succinate dehydrogenase flavoprotein, putative |
| | Tb927.8.6920 | hypothetical protein, conserved |
| **126** | Tb927.1.3050 | tRNA (Uracil-5-)-methyltransferase, putative |
| | Tb927.10.3630 | hypothetical protein, conserved |
| | Tb927.11.13280 | mitochondrial RNA binding protein 2 (GBP25) |
| | Tb927.11.1550 | XRN 5'-3' exonuclease N-terminus, putative |
| | Tb927.11.1710 | mitochondrial RNA binding protein 1, guide RNA-binding protein of 21 kDa (gBP21) |
| | Tb927.3.1590 | mitochondrial RNA binding complex 1 subunit (MRB1590) |
| | Tb927.3.3230 | NOL1/NOP2/sun family, putative |
| **127** | Tb927.10.6220 | 5'-3' exoribonuclease D (XRND) |
| | Tb927.3.1040 | unspecified product |
| | Tb927.5.2790 | mitochondrial DNA polymerase beta-PAK (Pol beta-PAK) |
| | Tb927.5.4040 | hypothetical protein, conserved |
| | Tb927.7.6800 | Alpha/beta hydrolase family, putative |
| | Tb927.9.5590 | DNA topoisomerase ii (TOP2) |
| **128** | Tb927.10.5770 | Transitional endoplasmic reticulum ATPase, putative, Valosin-containing protein, Cell division control protein 48 (VCP) |
| | Tb927.10.9430 | phosphoribosylpyrophosphate synthetase, putative (PRS) |
| | Tb927.11.3030 | phosphoribosylpyrophosphate synthetase, putative (PRS) |

| | | |
|---|---|---|
| | Tb927.5.2960 | phosphoribosylpyrophosphate synthetase, putative (PRS) |
| | Tb927.5.3170 | ribose-phosphate pyrophosphokinase, putative (PRPS5) |
| | Tb927.7.6910 | Phosphatidylinositol 4-phosphate 5-kinase, putative, MORN repeat-containing protein |
| | Tb927.8.7100 | acetyl-CoA carboxylase |
| **129** | Tb927.10.1060 | T-complex protein 1, delta subunit, putative (TCP-1-delta) |
| | Tb927.10.8190 | T-complex protein 1, theta subunit, putative, CCT-theta, putative |
| | Tb927.11.14250 | T-complex protein 1, epsilon subunit, putative (TCP-1-epsilon) |
| | Tb927.11.16760 | T-complex protein 1, alpha subunit, putative (TCP-1-alpha) |
| | Tb927.11.1900 | T-complex protein 1, beta subunit, putative |
| | Tb927.11.3240 | T-complex protein 1, zeta subunit, putative (TCP-1-zeta) |
| | Tb927.8.3150 | T-complex protein 1, gamma subunit, putative (TCP-1-gamma) |
| | Tb927.9.11270 | T-complex protein 1, eta subunit, putative, t- complex protein 1 (eta subunit), putative (TCP-1-eta) |
| **130** | Tb927.10.180 | ATP synthase F1 subunit gamma protein, putative |
| | Tb927.10.5050 | Mitochondrial ATP synthase epsilon chain, putative |
| | Tb927.11.13070 | O-phosphoseryl-tRNA(Sec) selenium transferase, putative |
| | Tb927.3.1380 | ATP synthase subunit beta, mitochondrial, ATP synthase F1, beta subunit (ATPB) |
| | Tb927.3.3410 | aspartyl aminopeptidase, putative, metallo-peptidase, Clan MH, Family M20 |
| | Tb927.5.1710 | ribonucleoprotein p18, mitochondrial precursor, putative |
| | Tb927.6.4990 | ATP synthase, epsilon chain, putative |
| | Tb927.7.7430 | ATP synthase alpha chain, mitochondrial precursor, ATP synthase F1, alpha subunit |
| **131** | Tb927.3.1680 | hypothetical protein, conserved |
| | Tb927.3.5470 | hypothetical protein, conserved |
| | Tb927.4.3560 | protein phoshatase 1, putative |
| | Tb927.8.4220 | hypothetical protein, conserved |
| **132** | Tb927.10.5860 | hypothetical protein, conserved |
| | Tb927.5.3970 | adenylate kinase, putative (ADKE) |
| | Tb927.6.2790 | L-threonine 3-dehydrogenase, putative |
| | Tb927.7.5160 | deoxyuridine triphosphatase, putative, dUTP diphosphatase |
| **133** | Tb927.10.13950 | tubulin-specific chaperone, putative |
| | Tb927.11.8770 | ATP-dependent RNA helicase FAL1, putative |
| | Tb927.8.1990 | peroxidoxin (TRYP2) |
| **134** | Tb927.1.1200 | SSU ribosomal protein, mitochondrial (MRPS15) |
| | Tb927.10.7380 | hypothetical protein, conserved |
| | Tb927.11.11630 | hypothetical protein, conserved |
| | Tb927.11.1250 | Mitochondrial SSU ribosomal protein, putative |
| | Tb927.11.6000 | ribosomal protein L4/L1 family, putative |
| | Tb927.11.870 | hypothetical protein, conserved |
| | Tb927.2.4890 | ribosomal protein L11, putative |
| | Tb927.3.5610 | ribosomal protein L3 mitochondrial, putative |
| | Tb927.5.3980 | hypothetical protein, conserved |

| | | |
|---|---|---|
| | Tb927.5.4120 | hypothetical protein, conserved |
| | Tb927.6.2080 | hypothetical protein, conserved |
| | Tb927.6.4560 | hypothetical protein, conserved |
| | Tb927.7.2760 | ribosomal protein L22p/L17e, putative |
| | Tb927.7.3030 | hypothetical protein, conserved |
| | Tb927.7.4140 | ribosomal protein L21, putative |
| | Tb927.8.5200 | hypothetical protein, conserved |
| | Tb927.9.7170 | Mitochondrial 39-S ribosomal protein L47 (MRP-L47), putative |
| | Tb927.9.8290 | hypothetical protein, conserved |
| **135** | Tb927.11.7170 | seryl-tRNA synthetase |
| | Tb927.5.4560 | guanine deaminase, putative, guanase, putative, guanine aminase, putative, guanine aminohydrolase, putative |
| | Tb927.8.1020 | 6-phosphofructo-2-kinase/fructose-2,6-biphosphatase, putative |
| | Tb927.9.7550 | adenylosuccinate lyase, putative (ADSL) |
| **136** | Tb927.10.8880 | hypothetical protein |
| | Tb927.9.12910 | unspecified product |
| **137** | Tb927.10.2350 | pyruvate dehydrogenase complex E3 binding protein, putative |
| | Tb927.9.7550 | adenylosuccinate lyase, putative (ADSL) |
| **138** | Tb927.10.10140 | paraflagellar rod component, putative (PFC19) |
| | Tb927.10.3710 | proteasome activator protein PA26 (pa26) |
| | Tb927.10.8060 | SET domain containing protein, putative |
| | Tb927.10.8360 | Poly(A)-specific ribonuclease PARN-2 |
| | Tb927.11.2630 | diphthamide biosynthesis enzyme Dph1/Dph2 domain containing protein, putative |
| **139** | Tb927.10.1930 | hypothetical protein, conserved |
| | Tb927.6.1830 | WD domain, G-beta repeat, putative |
| | Tb927.6.2380 | hypothetical protein, conserved |
| | Tb927.7.2450 | hypothetical protein, conserved |
| | Tb927.7.2520 | Uncharacterised protein family UPF0066, putative |
| | Tb927.7.4970 | glutamine synthetase, putative (GS) |
| **140** | Tb927.10.7550 | hypothetical protein, conserved |
| | Tb927.11.7520 | hypothetical protein, conserved |
| | Tb927.3.3330 | heat shock protein 20, putative |
| | Tb927.8.2020 | agmatinase, putative |
| **141** | Tb927.2.1890 | E2-like ubiquitin-conjugation enzyme (ATG3) |
| | Tb927.6.4950 | mago nashi-like protein, putative |
| **142** | Tb927.11.14120 | phenylalanyl-tRNA synthetase alpha chain, putative |
| | Tb927.4.1270 | ruvB-like DNA helicase, putative |
| | Tb927.4.2000 | ruvB-like DNA helicase, putative (RUVBL) |
| | Tb927.6.4670 | MORN repeat-containing protein 1 (MORN1) |
| | Tb927.7.5290 | hypothetical protein, conserved |
| | Tb927.9.5900 | glutamate dehydrogenase (GDH) |
| **143** | Tb927.1.3000 | amidohydrolase, putative |

| | | |
|---|---|---|
| | Tb927.10.14140 | pyruvate kinase 1 (PYK1) |
| | Tb927.11.6590 | aminopeptidase, putative, metallo-peptidase, Clan MF, Family M17 |
| | Tb927.6.2970 | pseudouridine synthase A-like protein, putative |
| | Tb927.7.3770 | YjeF family N-terminal domain/YjeF family C-terminal domain containing protein, putative |
| | Tb927.9.7540 | calpain-like cysteine peptidase, putative, cysteine peptidase, Clan CA, family C2, putative |
| **144** | Tb927.10.14680 | ribosome biogenesis protein, putative |
| | Tb927.10.8200 | Ribosomal protein L1p/L10e family, putative |
| | Tb927.11.1390 | class I transcription factor A, subunit 1 (CITFA-1) |
| | Tb927.11.14020 | RNA-binding protein (NRBD2) |
| | Tb927.11.1410 | class I transcription factor A, subunit 3 (CITFA-3) |
| | Tb927.11.14960 | pumilio/PUF RNA binding protein 7, putative (PUF7) |
| | Tb927.11.1670 | cysteine desulfurase |
| | Tb927.3.1350 | hypothetical protein, conserved |
| | Tb927.3.2830 | brix domain containing protein, putative |
| | Tb927.5.2080 | guanosine monophosphate reductase, putative |
| | Tb927.5.840 | KRI1-like family/KRI1-like family C-terminal, putative |
| | Tb927.5.970 | class I transcription factor A, subunit 6 (CITFA-6) |
| | Tb927.6.2050 | ribosome biogenesis regulatory protein (RRS1), putative |
| | Tb927.7.270 | ribosome biogenesis protein, putative |
| | Tb927.8.5040 | EMG1/NEP1 methyltransferase, putative |
| | Tb927.9.15060 | rRNA processing protein, putative |
| **145** | Tb927.10.11130 | SET domain containing protein, putative |
| | Tb927.10.12710 | heat shock protein 110, putative |
| | Tb927.11.6370 | leucine-rich repeat protein (LRRP), putative |
| **146** | Tb927.11.16020 | RNA-binding protein, putative (DRBD10) |
| | Tb927.11.16400 | kinetoplast-associated protein 3, putative (KAP3) |
| | Tb927.11.4690 | mitochondrial DNA polymerase I protein B (POLIB) |
| **147** | Tb927.10.1930 | hypothetical protein, conserved |
| | Tb927.10.2490 | glucose-6-phosphate 1-dehydrogenase (G6PD) |
| | Tb927.10.7420 | bromodomain factor 2 protein, putative |
| **148** | Tb11.v5.0325 | retrotransposon hot spot (RHS) protein, putative |
| | Tb11.v5.0713 | retrotransposon hot spot (RHS) protein, putative |
| | Tb927.11.2630 | diphthamide biosynthesis enzyme Dph1/Dph2 domain containing protein, putative |
| | Tb927.3.4040 | Ankyrin repeats (3 copies), putative |
| **149** | Tb927.11.15280 | tRNA-sepcific adenosine deaminase (ADAT3) |
| | Tb927.8.4180 | tRNA-specific adenosine deaminase (ADAT2) |
| **150** | Tb927.5.1270 | hypothetical protein, conserved |
| | Tb927.9.10200 | hypothetical protein, conserved |
| | Tb927.9.10880 | hypothetical protein, conserved |
| **151** | Tb927.10.7140 | membrane-bound acid phosphatase 2 (MBAP2) |
| | Tb927.8.5720 | Met-10+ like-protein, putative |

| | | |
|---|---|---|
| **152** | Tb927.1.1220 | RWD domain-containing protein |
| | Tb927.10.13650 | ARF-like 2-binding protein, putative |
| | Tb927.11.13690.1 | unspecified product |
| | Tb927.6.2860 | WD domain, G-beta repeat, putative |
| | Tb927.6.4420 | tRNA (Guanine-1)-methyltransferase, putative |
| | Tb927.9.9020 | ribosome-interacting GTPase 2, putative (RBG2) |
| **153** | Tb927.11.1300 | UBA/TS-N domain containing protein, putative |
| | Tb927.3.4340 | diphthamide synthesis protein, putative |
| | Tb927.6.2940 | phosphopantothenoylcysteine decarboxylase, putative (PPCDC) |
| | Tb927.9.12830 | hypothetical protein, conserved |
| **154** | Tb927.10.2990 | nuclear cap binding complex subunit CBP110 (CBP110) |
| | Tb927.7.6270 | peptidase t, putative, aminotripeptidase, putative |
| **155** | Tb927.10.8060 | SET domain containing protein, putative |
| | Tb927.11.11290 | heat shock protein 70, putative |
| | Tb927.7.6620 | Probable N6-adenine methyltransferase, putative |
| **156** | Tb927.10.3950 | hypothetical protein, conserved |
| | Tb927.5.1460 | Possible lysine decarboxylase, putative |
| | Tb927.5.3520 | queuine tRNA-ribosyltransferase, putative |
| | Tb927.6.3130 | queuine tRNA-ribosyltransferase, putative |
| | Tb927.9.5190 | proliferative cell nuclear antigen (PCNA), putative |
| **157** | Tb927.11.16510 | hypothetical protein, conserved |
| | Tb927.6.4210 | aldehyde dehydrogenase, putative (ALDH) |
| | Tb927.9.9710 | Histidine phosphatase superfamily (branch 1), putative |
| **158** | Tb927.10.8290 | eukaryotic translation initiation factor 3 subunit 8, putative |
| | Tb927.7.6090 | hypothetical protein, conserved |
| **159** | Tb927.6.4920 | S-adenosylmethionine synthetase, putative (METK1) |
| | Tb927.7.6450 | tRNA-dihydrouridine synthase 3, putative |
| **160** | Tb927.10.12710 | heat shock protein 110, putative |
| | Tb927.6.670 | ribosomal RNA processing protein 45, exosome complex exonuclease (RRP45) |
| | Tb927.7.710 | heat shock 70 kDa protein, putative (HSP70) |
| **161** | Tb927.11.10240 | hslVU complex proteolytic subunit, threonine peptidase, Clan T(1), family T1B, ATP-dependent protease subunit HslV (HsIV) |
| | Tb927.11.14120 | phenylalanyl-tRNA synthetase alpha chain, putative |
| | Tb927.11.2360 | phenylalanyl-tRNA synthetase (beta subunit), putative |
| **162** | Tb927.10.7110 | inositol-3-phosphate synthase, putative |
| | Tb927.11.16770 | glucosamine-6-phosphate isomerase, putative |
| **163** | Tb927.11.7060 | acidocalcisomal pyrophosphatase |
| | Tb927.2.450 | retrotransposon hot spot protein 4 (RHS4), putative |
| | Tb927.3.1550 | Rab3 GTPase-activating protein catalytic subunit, putative |
| | Tb927.5.3430 | ubiquitin-activating enzyme E1, putative |
| | Tb927.9.7540 | calpain-like cysteine peptidase, putative, cysteine peptidase, Clan CA, family C2, putative |
| **164** | Tb927.10.11310 | intraflagellar transport protein 57/55 (IFT57/55) |

| | | |
|---|---|---|
| | Tb927.10.13860 | GPI-anchor transamidase subunit 8 (GPI8) |
| | Tb927.10.4040 | 3-keto-dihydrosphingosine reductase |
| | Tb927.10.4610 | dolicholphosphate-mannose synthase, putative (DPMS) |
| | Tb927.11.13820 | hypothetical protein, conserved |
| | Tb927.11.15760 | GPI transamidase subunit Tta1 (TTA1) |
| | Tb927.2.1810 | transcription silencer (ISWI) |
| | Tb927.5.1930 | signal peptidase subunit, putative |
| | Tb927.8.5760 | Ankyrin repeats (many copies)/Alpha/beta hydrolase family, putative |
| **165** | Tb927.1.4690 | arginine N-methyltransferase (PRMT1) |
| | Tb927.10.12980 | Multisite-specific tRNA:(cytosine-C(5))-methyltransferase, putative |
| | Tb927.10.14750 | fibrillarin, putative |
| | Tb927.10.1960 | hypothetical protein, conserved |
| | Tb927.10.3560 | arginine N-methyltransferase, putative |
| | Tb927.10.7500 | fibrillarin (NOP1) |
| | Tb927.8.3750 | Nucleolar protein 56, putative (NOP56) |
| | Tb927.8.900 | splicing factor TSR1 (TSR1) |
| | Tb927.9.5320 | nucleolar RNA binding protein, putative |
| | Tb927.9.6870 | RNA-binding protein, putative (RBSR1) |
| **166** | Tb927.3.640 | hypothetical protein, conserved |
| | Tb927.5.1020 | disulfide isomerase, putative |
| | Tb927.6.1140 | dolichyl-P-Man:GDP-Man5GlcNAc2-PP-dolichyl alpha-1,2-mannosyltransferase, putative (ALG9) |
| | Tb927.6.1810 | Alpha/beta hydrolase family, putative |
| | Tb927.9.3770 | hypothetical protein, conserved |
| **167** | Tb927.10.2940 | Soluble NSF attachment protein, SNAP, putative |
| | Tb927.7.2260 | SEP domain containing protein, putative |
| **168** | Tb927.10.390 | DUF2407 ubiquitin-like domain containing protein, putative |
| | Tb927.7.6850 | trans-sialidase (TS) |
| **169** | Tb927.11.10960 | 2OG-Fe(II) oxygenase superfamily, putative |
| | Tb927.8.4100 | hypothetical protein, conserved |
| **170** | Tb927.5.930 | NADH-dependent fumarate reductase (FRDg) |
| | Tb927.7.2440 | pyrroline-5-carboxylate reductase, putative (P5CR) |
| **171** | Tb927.11.11380 | hypothetical protein, conserved |
| | Tb927.6.650 | hypothetical protein, conserved |
| **172** | Tb927.11.9700 | nascent polypeptide associated complex alpha subunit, putative |
| | Tb927.7.5490 | arginine N-methyltransferase, type III (PRMT7) |
| **173** | Tb927.1.2750 | hypothetical protein, conserved |
| | Tb927.7.5680 | deoxyribose-phosphate aldolase, putative |
| **174** | Tb927.10.8720 | CCR4-NOT transcription complex subunit 10, putative |
| | Tb927.8.1960 | hypothetical protein, conserved |
| **175** | Tb927.8.1590 | ubiquitin-protein ligase, putative (upl3) |

| | | |
|---|---|---|
| | Tb927.9.13610 | helicase, putative |
| **176** | Tb927.1.120 | retrotransposon hot spot protein 4 (RHS4), putative |
| | Tb927.9.13990 | RNA-binding protein, putative (DRBD2) |
| **177** | Tb927.9.15150 | unspecified product |
| | Tb927.9.4200 | fatty acyl CoA synthetase 2 (ACS2) |
| **178** | Tb927.4.1250 | peroxisome biogenesis factor 1, putative |
| | Tb927.5.3920 | peroxisome assembly protein, putative |
| **179** | Tb11.v5.0746 | tatD related deoxyribonuclease, putative |
| | Tb927.10.4640 | eukaryotic translation initiation factor 3 subunit L, putative (EIF3L) |
| | Tb927.11.15420 | COP9 signalosome, subunit CSN8, putative |
| **180** | Tb927.11.15430 | U5 small nuclear ribonucleoprotein component, putative, U5 snrnp-specific protein, putative |
| | Tb927.5.2290 | ATP-dependent RNA helicase, putative |
| | Tb927.9.11110 | PRP8 protein homologue, U5 snRNA-associated splicing factor |
| **181** | Tb927.11.14200 | ubiquitin-conjugating enzyme E2, putative |
| | Tb927.7.1020 | hypothetical protein, conserved |
| | Tb927.8.5880 | eukaryotic translation initiation factor 1A, putative |
| **182** | Tb927.11.2340 | hypothetical protein, conserved |
| | Tb927.11.2370 | mRNA export factor MEX67, Nuclear RNA export factor (MEX67) |
| | Tb927.5.4380 | kinetoplastid-specific phospho-protein phosphatase, putative |
| | Tb927.8.1840 | Sec7 domain containing protein, putative |
| **183** | Tb927.3.4850 | enoyl-CoA hydratase, mitochondrial precursor, putative |
| | Tb927.6.4540 | 3-hydroxy-3-methylglutaryl-CoA reductase, putative |
| | Tb927.7.1080 | hypothetical protein, conserved |
| | Tb927.8.5510 | apurinic/apyrimidinic endonuclease, putative |
| **184** | Tb927.10.14180 | protein transport protein SEC13, putative |
| | Tb927.11.3770 | Dpy-30 motif containing protein, putative |
| | Tb927.8.4790 | Putative snoRNA binding domain containing protein, putative |
| | Tb927.9.5410 | hypothetical protein, conserved |
| **185** | Tb927.10.1490 | Temperature dependent protein affecting M2 dsRNA replication, putative |
| | Tb927.6.2640 | importin alpha subunit, putative (TbKap60) |
| **186** | Tb927.11.230 | cleavage and polyadenylation specificity factor, putative |
| | Tb927.4.1340 | cleavage and polyadenylation specificity factor subunit, putative (CPSF3) |
| **187** | Tb927.10.11300 | paraflagellar rod component, putative (PFC16) |
| | Tb927.6.4140 | paraflagellar rod component, putative (PFC4) |
| **188** | Tb927.11.9880 | Protein of unknown function (DUF2009), putative |
| | Tb927.9.10690 | Protein of unknown function (DUF2009), putative |
| **189** | Tb927.1.2570 | coatomer beta subunit (beta-coP) |
| | Tb927.10.7060 | nucleoporin interacting component (NUP93), putative |
| | Tb927.11.11900 | Coatomer subunit gamma (COPG) |
| | Tb927.11.14970 | hypothetical protein, conserved |

| | | |
|---|---|---|
| | Tb927.11.5400 | signal recognition particle 54 kDa (SRP54) |
| | Tb927.7.1920 | paraflagellar rod component, putative (PFC5) |
| **190** | Tb927.1.3830 | glucose-6-phosphate isomerase, glycosomal (PGI) |
| | Tb927.10.13430 | citrate synthase, putative |
| | Tb927.9.12110 | 6-phosphogluconate dehydrogenase, decarboxylating (gnD) |
| **191** | Tb927.4.3950 | cytoskeleton-associated protein CAP5.5, putative, cysteine peptidase, Clan CA, family C2, putative, Calpain-like protein 1 (CAP5.5) |
| | Tb927.9.2470 | nucleolar protein (NOP86) |
| **192** | Tb927.4.2670 | Cysteine peptidase, Clan CF, family C15, pyroglutamyl-peptidase I, putative (PPI) |
| | Tb927.5.950 | monothiol glutaredoxin, putative |
| | Tb927.7.6890 | methyltransferase domain containing protein, putative |
| | Tb927.8.4930 | hypothetical protein, conserved |
| | Tb927.9.7000 | methyltransferase domain containing protein, putative |
| **193** | Tb927.3.860 | Acyl carrier protein, mitochondrial, NADH-ubiquinone oxidoreductase complex I subunit, putative (ACP) |
| | Tb927.9.9840 | lipoic acid containing carrier protein, putative (GCVH) |
| **194** | Tb927.11.180 | electron transfer flavoprotein, putative |
| | Tb927.11.7540 | electron-transfer-flavoprotein, alpha polypeptide, putative |
| **195** | Tb927.11.14870 | NAD+ synthase, putative |
| | Tb927.4.2700 | Hydroxymethylglutaryl-CoA lyase, mitochondrial, putative |
| **196** | Tb927.4.3350 | N2227-like protein, putative |
| | Tb927.9.1510 | D-ala D-ala ligase C-terminus/SET domain containing protein, putative |
| **197** | Tb927.5.2690 | inositol-1(or 4)-monophosphatase 1, putative (IMPase 1) |
| | Tb927.9.6350 | inositol-1(or 4)-monophosphatase, putative (IMPase) |
| **198** | Tb927.5.1000 | ubiquitin-conjugating enzyme E2, putative, ubiquitin carrier protein, putative, ubiquitin-protein ligase, putative |
| | Tb927.9.1520 | hypothetical protein, conserved |
| **199** | Tb927.7.4360 | hypothetical protein, conserved |
| | Tb927.8.6930 | serine/threonine-protein kinase NrkA (NRKB) |
| **200** | Tb927.6.2330 | RGG protein (RGG1) |
| | Tb927.7.1790 | Adenine phosphoribosyltransferase, putative |
| **201** | Tb927.6.2200 | DJ-1 family protein, putative |
| | Tb927.8.1440 | maoC-like dehydratase, putative |
| **202** | Tb927.10.8920 | ras-like small GTPase, putative (TbGRP) |
| | Tb927.11.3320 | ras-like small GTPase, putative (TbGTR) |
| **203** | Tb927.11.3310 | ubiquitin-conjugating enzyme, putative |
| | Tb927.9.8000 | ubiquitin-conjugating enzyme E2, putative, ubiquitin carrier protein, putative, ubiquitin-protein ligase, putative |
| **204** | Tb927.3.3450 | ADP-ribosylation factor-like protein 3, putative (arl3) |
| | Tb927.3.3780 | tryparedoxin 1a, putative (TXN1a) |
| **205** | Tb927.10.8360 | Poly(A)-specific ribonuclease PARN-2 |
| | Tb927.3.4490 | protein farnesyltransferase alpha subunit, putative |
| | Tb927.7.460 | protein farnesyltransferase beta subunit (TbPFT) |

| | | |
|---|---|---|
| **206** | Tb927.3.4040 | Ankyrin repeats (3 copies), putative |
| | Tb927.7.4000 | glutathione synthetase, putative |
| **207** | Tb927.10.7060 | nucleoporin interacting component (NUP93), putative |
| | Tb927.10.8170 | nuclear pore complex protein (NUP155), putative, nucleoporin, putative |
| | Tb927.9.2320 | methyltransferase domain containing protein, putative (POMP1) |
| **208** | Tb927.11.7840 | ribonucleoside-diphosphate reductase large chain (RNR1) |
| | Tb927.3.610 | N-acetyltransferase complex ARD1 subunit, putative |
| | Tb927.6.1950 | N-acetyltransferase B complex (NatB) non catalytic subunit, putative |
| **209** | Tb927.7.1120 | trypanothione/tryparedoxin dependent peroxidase 1, cytosolic, glutathione peroxidase-like protein 1 (TDPX1) |
| | Tb927.7.1130 | trypanothione/tryparedoxin dependent peroxidase 2, glutathione peroxidase-like 2 (TDPX2) |
| **210** | Tb927.11.7100 | cytoplasmic translation machinery associated protein, putative |
| | Tb927.8.4330 | small GTP-binding protein Rab11 (RAB11) |
| **211** | Tb927.11.2090 | choline kinase (EK1) |
| | Tb927.6.1530 | Glutamine amidotransferase class-I, putative |
| **212** | Tb927.10.3260 | Long-chain-fatty-acid--CoA ligase 5 (EC 6.2.1.3) (Long-chain acyl-CoA synthetase 5) (LACS 5), putative |
| | Tb927.2.4130 | enoyl-CoA hydratase/Enoyl-CoA isomerase/3-hydroxyacyl-CoA dehydrogenase, putative |
| **213** | Tb927.1.1270 | homocysteine S-methyltransferase, putative |
| | Tb927.11.13730 | ornithine decarboxylase (ODC) |
| | Tb927.4.1740 | hypothetical protein, conserved |
| | Tb927.6.1780 | mitogen-activated protein kinase, putative, protein kinase, putative |
| | Tb927.8.2690 | SET domain containing protein, putative |
| **214** | Tb927.11.5440 | malic enzyme |
| | Tb927.11.5450 | malic enzyme |
| **215** | Tb927.10.7930 | 2,3-bisphosphoglycerate-independent phosphoglycerate mutase (PGAM) |
| | Tb927.11.8970 | ribose 5-phosphate isomerase, putative |
| | Tb927.4.3320 | uracil phosphoribosyltransferase, putative |
| | Tb927.9.7770 | spermidine synthase (SpSyn) |
| **216** | Tb927.6.4480 | valyl-tRNA synthetase, putative (ValRS) |
| | Tb927.6.4920 | S-adenosylmethionine synthetase, putative (METK1) |
| **217** | Tb927.10.6970 | dipeptidyl-peptidase 8-like serine peptidase, serine peptidase, Clan SC, Family S9B |
| | Tb927.10.8060 | SET domain containing protein, putative |
| | Tb927.11.7150 | NGG1 interacting factor 3-like |
| | Tb927.7.1910 | pyridoxal phosphate containing glycine decarboxylase, putative (GCVP) |
| | Tb927.9.9820 | glyceraldehyde-3-phosphate dehydrogenase, putative |
| **218** | Tb927.11.6210 | sterol 14-alpha-demethylase (CYP51) |
| | Tb927.7.210 | proline dehydrogenase |
| | Tb927.9.4190 | fatty acyl CoA syntetase 1 (ACS1) |

| | | |
|---|---|---|
| **219** | Tb927.2.4110 | metallo-peptidase, Clan ME, Family M16, Mitochondrial-processing peptidase subunit alpha (MPPA) |
| | Tb927.4.600 | Alpha/beta hydrolase family, putative |
| | Tb927.9.4520 | metallo-peptidase, Clan ME, Family M16, Mitochondrial-processing peptidase subunit beta (MPPB) |
| **220** | Tb927.3.2090 | aminopeptidase P1, putative, metallo-peptidase, Clan MG, Family M24, Xaa-Pro aminopeptidase, putative |
| | Tb927.8.2640 | ubiquitin-activating enzyme E1, putative (UBA1) |
| **221** | Tb927.1.1380 | serine/threonine protein phosphatase 2A regulatory subunit, putative |
| | Tb927.6.4760 | T-complex protein 11, putative |
| **222** | Tb927.1.3950 | alanine aminotransferase (ALAT) |
| | Tb927.11.3570 | aminopeptidase, putative, metallo-peptidase, Clan MA(E) Family M1 |
| | Tb927.11.5090 | aspartate aminotransferase, mitochondrial |
| **223** | Tb927.11.11250 | cytosolic malate dehydrogenase (cMDH) |
| | Tb927.8.4430 | uridine phosphorylase |
| **224** | Tb927.10.4000 | methylglutaconyl-CoA hydratase, mitochondrial precursor, putative |
| | Tb927.2.4590 | branched-chain amino acid aminotransferase, putative |
| | Tb927.7.2100 | GMP synthase, putative, glutamine amidotransferase, putative |
| | Tb927.8.980 | phosphoacetylglucosamine mutase, putative, acetylglucosaminephosphomutase, putative, N-acetylglucosamine-phosphate mutase, putative |
| **225** | Tb927.10.13130 | UTP-glucose-1-phosphate uridylyltransferase |
| | Tb927.10.14780 | mitogen-activated protein kinase kinase kinase, putative (CBPK1) |
| | Tb927.9.1960 | nitrilase, putative |
| **226** | Tb927.11.16480 | enoyl-CoA hydratase/isomerase family protein, putative |
| | Tb927.6.2360 | adenosine kinase, putative |
| | Tb927.8.5600 | transaldolase, putative |
| **227** | Tb927.11.1560 | 1,2-Dihydroxy-3-keto-5-methylthiopentene dioxygenase, putative |
| | Tb927.11.15910 | iron superoxide dismutase |
| | Tb927.7.1780 | Adenine phosphoribosyltransferase, putative |
| **228** | Tb927.9.13490 | aminopeptidase P, putative, metallo-peptidase, Clan MG, Family M24 |
| | Tb927.9.2010 | kynureninase, putative |
| **229** | Tb927.7.5210 | Putative Phosphatase/Protein of unknown function DUF89, putative |
| | Tb927.8.1860 | pitrilysin-like metalloprotease, metallo-peptidase, Clan ME, Family M16C |
| **230** | Tb927.10.10390 | trypanothione reductase |
| | Tb927.10.11970 | glutamine aminotransferase (GlnAT) (GlnAT) |
| **231** | Tb927.3.2960 | inosine-adenosine-guanosine-nucleosidehydrolase, IAG-nucleoside hydrolase (IAGNH) |
| | Tb927.5.3830 | dihydroorotate oxidase |
| | Tb927.6.2740 | pyridoxal kinase (pdxK) |
| **232** | Tb927.11.6870 | 14-3-3 protein |
| | Tb927.11.9530 | 14-3-3-I protein |
| **233** | Tb927.10.14840 | Mitochondrial ADP/ATP carrier protein 5a, putative (MCP5a) |

| | | |
|---|---|---|
| | Tb927.2.2520 | voltage-dependent anion-selective channel 2, Mitochondrial outer membrane protein porin 2 (VDAC2) |
| **234** | Tb927.10.11120 | hypothetical protein, conserved |
| | Tb927.11.1600 | tatD related deoxyribonuclease, putative |
| | Tb927.8.3550 | mitogen-activated protein kinase 3, putative |

**Supp Table 3. List of suggested names for complexes predicted by machine learning analysis.**

Complex number matches those described in Supp Table 2. The proportion of proteins within the complex  annotated as ‘hypothetical’ or classed as ‘essential’ in any life-cycle stage (4) are also displayed. The description highlights the proteins identified within the complex and denoting where there is orthogonal information for protein interaction. Complexes annotated as ‘Complex mixture’ contain more than two proteins with a number of different proposed functional properties or sub-cellular localisations.

| Complex no. | Name | Propor-tion ‘hypothe-tical’ | Propor-tion 'essen-tial' | Description |
|---|---|---|---|---|
| **1** | Glycerophospholipid metabolism complex | 0/4 | 1/4 | 2 of 4 are components of glycerophospholipid metabolism - glycerophosphoryl diester phosphodiesterase; glycerol-3-phosphate dehydrogenase |
| **2** | DNA topoisomerase IB and hypothetical | 1/2 | 1/2 | Hypothetical described as localised to FAZ |
| **3** | AMPK | 1/5 | 1/5 | AMPK beta and gamma (28), two predicted AMPK alpha subunits and a hypothetical protein |
| **4** | Mitochondrial ribosome complex 1 | 3/6 | 2/6 | 5 of 6 have been identified in multiple mitochondrial ribosome Ips, including MRPL13 and KRIT2 (27), and one remaining is a DEAD-box helicase, with homolofy to RRP3 |
| **5** | Complex mixture 1 | 0/6 | 3/6 | GAPDH, HSP70, quenine tRNA ribosyltransferase, METK1, NGG1 interacting factor 3-like protein and PCNA |
| **6** | Membrane protein complex | 1/15 | 5/15 | Membrane transporters of multiple categories including 3 multi-drug resistance transporters and two genes linked to pentamidine efficacy and resistance (42) |
| **7** | Complex mixture 2 | 0/6 | 2/6 | RNA binder, fructose-bisphosphate aldolase, ribonuclease, RHS, DUF2779 and tubulin tyrosine ligase |
| **8** | Complex mixture 3 | 2/6 | 0/6 | Pex19, a dynein light chain, tryptophanyl tRNA synthetase, inosine-guanine hydrolase and two hypothetical proteins |

| | | | | |
|---|---|---|---|---|
| **9** | Deoxyhypusine synthase | 0/2 | 0/2 | Interaction demonstrated in (43) |
| **10** | RuvB helicase complex and mixture 1 | 1/8 | 5/8 | Two ruvB helicases with homology to RUVBL1 and 2, thought to interact in a dodecamer. Other proteins sinclude a SET domain, glutamate dehydrogenase, carnitine O-acetyltransferase, hypotheitcal, cysteinyl tRNA synthase, hsIVU protease |
| **11** | Threonyl tRNA synthase and hypotheticals complex | 2/4 | 1/4 | Two hypothetical proteins (consistent co-elution), ARM domain protein and threonyl tRNA synthase |
| **12** | Heat shock protein 70/90 complex | 1/5 | 1/5 | HSP70 and HSP90, a S/T phosphatase 5 (interacting with HSP90 in (35)) a WD domain and hypothetical protein |
| **13** | Exon junction complex | 0/3 | 0/3 | Y14 and mago-nashi protein, shown to interact in META domain (DUF1935) |
| **14** | Hypothetical complex 1 | 3/3 | 2/3 | 3 hypothetical proteins. |
| **15** | tRNA synthetase complex | 2/5 | 2/5 | 3 putative tRNA synthetases (tyrosyl/methionyl, glutaminyl and prolyl), and two hypothetical proteins |
| **16** | Pyruvate dehydrogenase complex | 0/4 | 3/4 | PDH E1 alpha and beta, dihydrolipoamide acetyltransferase E2 and threonyl-tRNA transferase |
| **17** | Nucelosome assembly complex | 0/4 | 0/4 | 2 nucleosome assembly proteins, ARM like and leucine-repeat protein |
| **18** | E2 ubiquitin conjugating enzyme and CS domain complex | 0/2 | 1/2 | E2 like enzyme (ATG3) + CS domain containing protein |
| **19** | Major vault protein complex | 1/4 | 0/4 | three major vault proteins + hypothetical |
| **20** | Trafficking protein particle complex | 0/2 | 0/2 | Transport protein particle complex putative subunits 5 and 2 |
| **21** | Proteasome activator and ankyrin repeat complex | 0/2 | 0/2 | Proteasome activator and ankyrin repeat complex |
| **22** | Ribosomal complex 1 | 0/7 | 3/7 | L35a, L7a, L7, L13 and L6 components of 60S + L14 of 40s and ribosomal protein S7 |
| **23** | SET domain protein and 60S ribosome complex | 0/2 | 1/2 | SET domain protein and putative RPL10A 60S ribosome protein |
| **24** | Heat shock protein and coronin (CRN12) complex | 0/2 | 1/2 | HSP and CRN12 |
| **25** | Ribosomal complex 2 | 0/13 | 6/13 | S19, L18 ribo proteins and L34, L10, L19, L13a, RPL27a/L27a/L28, L21e, L23, L9 of 60S and S16, S4, S8 of 40S |
| **26** | Vacuolar ATP synthase | 0/4 | 0/4 | 4 vacuolar type ATP synthases, all linked to isometamidum resistance (44) |

| | | | | |
|---|---|---|---|---|
| **27** | tRNA methyltransferase complex | 0/5 | 0/5 | Gcd10p and conserved protein (putative tRNA adenine methyltransferases). NOL1/NOP2/sun family - methyltransferase BLAST + 2 RHS proteins |
| **28** | Exosome complex | 2/12 | 5/12 | EAP1, EAP2, RRP4, RRP45, RRP41A, RRP40, CSL4 (Estevez 2001 and 2003)+ hypothetical, HSP70, translation elongation factor, RNApolI subunit (RPA135), conserved ATP binding |
| **29** | Prefoldin complex | 0/4 | 0/4 | 4 prefoldin components |
| **30** | Proteasome nonATPase regulatory complex | 0/14 | 11/14 | RPT5, RPT4, RPN5, RPN6, RPN7, RPT2, RPT3, RPT6, RPN11, RPN9 + three more unnumbered + CIFTA-4 transcription factor |
| **31** | Proteasome core complex | 0/14 | 11/14 | 14 subunits including one termed unspecified product that BLASTs as core proteasome subunit |
| **32** | Ribosomal complex 3 | 0/2 | 1/2 | L38 and L44 of 60S ribosome |
| **33** | Ribosomal complex 4 | 0/2 | 0/2 | 2 acidic ribosomal proteins |
| **34** | Hypothetical complex 2 | 2/2 | 0/2 | 2 hypothetical proteins |
| **35** | Transportin and exosome protein | 0/2 | 1/2 | Transportin and exosome (RRP44p) proteins |
| **36** | Adenylyl cyclase associated and RNA binding protein | 0/2 | 0/2 | Adenyly cyclase associated protein and RBP |
| **37** | Tudor domain containing protein HSP84 | 0/2 | 1/2 | Tudor domain protein and HSP84 |
| **38** | 2-oxoglutarate dehydrogenase complex | 0/3 | 2/3 | 2 E1 and 1 E2 2-oxoglutarate dehydrogenase components |
| **39** | Dynein light chain and putative tRNA methyltransferase | 0/2 | 2/2 | Dynein light chain and tRNA methyltransferase |
| **40** | Mitochondrial processing peptidase complex | 0/2 | 0/2 | alpha and beta subunits |
| **41** | NOT5 and DUF protein | 0/2 | 0/2 | NOT5 and DUF protein |
| **42** | Radial spoke protein complex | 0/2 | 2/2 | RSP4/6 and RSP10 |
| **43** | Hypothetical complex 3 | 2/2 | 1/2 | Two hypothetical proteins |
| **44** | eIF3 complex | 0/2 | 1/2 | eIF3I and eIF3B |
| **45** | Hypothetical complex 4 | 2/2 | 1/2 | Two hypothetical proteins |
| **46** | Resolvase domain containing protein and cysteine peptidase | 0/2 | 2/2 | Resolvase domain containing protein and cysteine peptidase |

| | | | | |
|---|---|---|---|---|
| **47** | SEC24.1 and hypothetical protein | 1/2 | 1/2 | SEC24.1 and hypothetical protein |
| **48** | TatD related DNase and pescadillo domain containing protein | 0/2 | 1/2 | TatD related DNase and pescadillo domain containing protein |
| **49** | Mitochondrial elongation factor and HSP10 | 0/2 | 1/2 | Mitochondrial elongation factor and HSP10 |
| **50** | Paraflagellar rod protein and NADH fumarate reductase | 0/2 | 1/2 | Paraflagellar rod protein and NADH fumarate reductase |
| **51** | eIF4e and hypothetical protein | 1/2 | 1/2 | eIF4e and hypothetical protein |
| **52** | AMP deaminase and ZFP family member ZC3H10 | 0/2 | 1/2 | AMP deaminase and ZFP family member ZC3H10 |
| **53** | mitochondrial RNA binding protein and ATP-dependent DEAD/H RNA helicase | 0/2 | 1/2 | mitochondrial RNA binding protein and ATP-dependent DEAD/H RNA helicase |
| **54** | polyA binding complex protein and zinc finger protein | 1/2 | 1/2 | polyA binding complex protein and zinc finger protein |
| **55** | Hypothetical and galactose oxidase domain | 1/2 | 0/2 | Hypothetical and galactose oxidase domain |
| **56** | Cysteine peptidase complex | 0/2 | 0/2 | two C2 domain containing proteins complex |
| **57** | GrpE and small glutamine tetratricopeptide repeat protein | 0/2 | 1/2 | GrpE and small glutamine tetratricopeptide repeat protein |
| **58** | Mitochondrial ribosome complex 2 | 2/2 | 0/2 | 2 hypothetical proteins, both identified in (27), one MRPL29, and other also annotated as MAPK5 |
| **59** | Hypothetical protein and ras like GTPase (NST) | 1/2 | 0/2 | Hypothetical protein and ras like GTPase (NST) |
| **60** | Putative transport protein complex | 1/2 | 1/2 | Vta1 (similar to vacuolar protein sorting associated) and hypothetical (blast match to USX1, intracellular transport protein) |
| **61** | NMD3 and regulator of chrosome condensation RCC1 | 0/2 | 1/2 | RCC1 (annotated as ISWI complex, E3 ligase or GTPase) and NMD3 is ribosomal export protein |

| | | | | |
|---|---|---|---|---|
| **62** | AcylCoA dehydrogenase and hypothetical mitochondrial import protein | 1/2 | 2/2 | Hypothetical protein annotated as Tim62, a mitochondrial membrane transport complex involved in tRNA import. Both proteins identified in a pulldown of Tim17 (45) |
| **63** | Kinesin complex | 0/2 | 1/2 | Two kinsesins, kin-C and kin-D, shown to interact in (46) |
| **64** | Hypothetical complex 5 | 2/2 | 1/2 | Both annotated with BLAST hit - trichohyalin. One also as myosin heavy chain and other also inner centromere protein |
| **65** | DIGIT and NPAPL | 0/2 | 2/2 | A polyA polymerase protein and flagellar protein |
| **66** | Replication factor and glycosomal malate deydrogenase | 0/2 | 1/2 | Replication factor and glycosomal malate deydrogenase |
| **67** | ATG24 phosphoinositide binding complex | 0/2 | 0/2 | Both proteins contain PX domains and linked to suramin efficacy and resistance (42), both classed as SNX proteins previously with one classed as ATG24 (47) |
| **68** | cAMP specific phosphodiesterase complex | 0/2 | 1/2 | PDEB1 and 2 |
| **69** | Hypothetical complex 6 | 2/2 | 2/2 | Both BLASTs match Rad50 ATPase |
| **70** | Flagellar protein complex | 0/2 | 1/2 | KH1 (flagellum targetting protein) and PFC7 (paraflagellar protein) |
| **71** | 2-oxoisovalerate dehydrogenase complex | 0/2 | 0/2 | alpha and beta subunits |
| **72** | PUF10 complex | 1/2 | 1/2 | PUF10 and hypothetical protein |
| **73** | Pyruvate dehydrogenase kinase complex | 0/2 | 0/2 | One named developmental regulated phospho-protein - BLASTs also as PDH kinase |
| **74** | SRP68-72 complex | 1/3 | 3/3 | SRP72 and 68 and glutamine hydrolysing carbomoyl phosphate synthase. Interaction between SRPs are identified previously in (48) |
| **75** | Translation associated complex | 1/3 | 2/3 | BFR (associated with yeast polysomal mRNP, also chromosome segregation), hypothetical protein (BLAST to Bromodomain chromosome ass./RNAPII deg factor/splicing factor) and MKT1 (translation elongation in yeast) |
| **76** | H/ACA ribonucleoprotein complex | 0/3 | 0/3 | Cbf5p, GAR1 and putative Nhp2 |
| **77** | Pseudouridine synthase complex | 0/3 | 0/3 | 2 pseudouridine synthases and a putative tRNA methyltransferase |
| **78** | GRESAG complex | 0/3 | 0/3 | Three receptor type adenylate cyclase |
| **79** | N-acetyltransferase complex | 0/3 | 2/3 | Three N-acetyltransferase subunit |
| **80** | Heat shock protein 70 complex | 0/3 | 1/3 | 2 HSP70s and BiP (also with HSP70 homology) |
| **81** | snRNP complex | 0/3 | 2/3 | SmD2, SmB, SmD3 |

| | | | | |
|---|---|---|---|---|
| **82** | Vaculolar sorting, Ran binding and hypothetical protein | 1/3 | 1/3 | Vaculolar sorting, Ran binding and hypothetical protein |
| **83** | Translation initiation factor, flagellar protein and hypothetical flagellar protein | 1/3 | 0/3 | Translation initiation factor, flagellar protein and hypothetical flagellar protein |
| **84** | ALBA complex | 0/3 | 2/3 | ALBA1, 3 and 4 |
| **85** | F1/F0 ATP synthase complex 1 | 3/3 | 1/3 | 2 mitochondrial ATP synthase components identified in (26) and a hypothetical protein |
| **86** | U6 spliceosome complex | 0/3 | 0/3 | LSm7p, LSm4p and PARN1. LSm proteins identified interacting in (Tkacz et al 2008) |
| **87** | Histone complex | 0/3 | 0/3 | H2A, H2B, H4 |
| **88** | Complex mixture 4 | 0/3 | 2/3 | Ribosome biogenesis, nucleolar RNA helicase and DNA topoisomerase proteins |
| **89** | Hypothetical + DUF proteins | 1/2 | 1/2 | Hypothetical + DUF proteins |
| **90** | Complex mixture 5 | 1/3 | 0/3 | AMP binding protein thought to be part of NADH-ubiquinone oxidoreductase complex |
| **91** | Hypothetical complex 7 | 3/3 | 1/3 | 3 hypothetical proteins, one associated with cytoskeleton and one with ISWI domain homology. |
| **92** | Mitochondrial ribosome complex 3 | 2/3 | 0/3 | All identified in (27) |
| **93** | Complex mixture 6 | 1/3 | 0/3 | Vesicular fusion protein SEC18, hypothetical protein and cold-shock DNA binding domain protein |
| **94** | Complex mixture 7 | 1/3 | 2/3 | NUP-1 protein with CEP250 domain, hypothetical protein annotated as FAZ10 also with predicted CEP250 domain and SRP40 C-terminal domain protein |
| **95** | Complex mixture 8 | 2/3 | 1/3 | DNA directed RNA polymerase and two hypotheticals |
| **96** | Complex mixture 9 | 2/3 | 0/3 | 2 hypotheticals annotated as flagellar protein and POMP respectively and an endo-beta-N-acetylglucosaminadase |
| **97** | Complex mixture 10 | 0/3 | 2/3 | Lsm12 protein, cytoskeleton associated protein 17 and RNA binding protein 20 |
| **98** | Mitochondrial inner membrane Tim10 and hypothetical protein | 1/2 | 0/2 | Mitochondrial inner membrane Tim10 and hypothetical protein |
| **99** | mRNA methytransferase cap and elongation factor complex | 1/5 | 1/5 | 3 elongation factors, cap methyltransferase and hypothetical shown to associate in (37) |
| **100** | Complex mixture 11 | 0/4 | 0/4 | Twy3 homolog, tRNA methyltransferase, two pyrophosphorylases and a WD domain containing protein |

| | | | | |
|---|---|---|---|---|
| **101** | Complex mixture 12 | 1/4 | 1/4 | Pestivirus endopeptidase, a hypothetical protein, lysosomal alpha-mannosidase and leucine rich repeat protein |
| **102** | Complex mixture 13 | 0/4 | 2/4 | valsolin containing protein, WD40 ribosome biogensis protein, nucleolar GTP binding protein NOG1 and ribosome production factor protein |
| **103** | Complex mixture 14 | 2/4 | 0/4 | 2 hypothetical proteins, an oxidoreductase protein and leucine carboxymethyltransferase |
| **104** | Hypothetical and transport protein | 1/2 | 0/2 | Hypothetical protien and transport protein particle complex component |
| **105** | Peptidase complex | 0/3 | 1/3 | M76 and papain cysteine peptidase and DNL Zn finger type protein with Tim15 domain homology. M76 peptidase also has homology to ATP23 a mitochondrial inner membrane protein |
| **106** | 1,2-diOH-3-keto-5-methylthiopentene deoxygenase and hypothetical | 1/2 | 1/2 | Hypothetical identified in (27) as mitochondrial ribosome subunit |
| **107** | Complex mixture 15 | 0/3 | 2/3 | RNA binding protein 17, mRNA export receptor Mtr2 and Zn finger containing protein |
| **108** | U3 ribonucleoprotein sub-complex | 0/4 | 1/4 | Pwp2 yeast homolog and three proteins containing Utp21, Utp13 and Utp12 domains indicitative of U3 ribonucleoprotein complex |
| **109** | Hypothetical protein and RNA binder (RBP29) | 1/2 | 0/2 | Hypothetical protein and RNA binder (RBP29) |
| **110** | Kinesin complex II | 2/4 | 2/4 | One hypothetical with flagellar pocket localisation, two kinesins and another hypothetical protein |
| **111** | Complex mixture 16 | 0/4 | 1/4 | GAPDH, cyclophilin, glycerol-3-phosphate dehydrogenase and fructose 1-6-biphosphatase |
| **112** | ARP2/3 complex | 0/5 | 0/5 | 5 ARP2/3 components |
| **113** | 3-methylcrotonyl-coA carboxylase and dihydrolipoamide dehydrogenase complexes | 0/5 | 0/5 | 3-methylcrotonyl-coA carboxylase alpha and beta subunits and three dihydrolipoamide dehydrogenase subunits |
| **114** | Complex mixture 17 | 1/4 | 2/4 | 2 predicted 40S ribosomal components, a Zn finger protein and a hypothetical protein |
| **115** | Hypothetical complex 8 | 3/4 | 1/4 | Intraflagellar transport hypothetical protein, a START domain protein and a hypothetical protein with motile cillium GO annotation |
| **116** | Complex mixture 18 | 1/5 | 0/5 | Mak10 N-acetyltransferase, Cgm1 mRNA N-methyltransferase, a third N-acetyltransferase, a hypothetical protein and a predicted heat shock protein |
| **117** | Complex mixture 19 | 1/5 | 3/5 | Nup149, SEC31, RBP16, a hypothetical annotated as RBP8 and poly(A) export protein GLE2 |

| | | | | |
|---|---|---|---|---|
| **118** | Complex mixture 20 | 1/3 | 1/3 | A hypothetical protein, a KRE33 ribosomal biogenesis protein, and UTP15 C-terminal domain putative U3 ribonucleoprotein component |
| **119** | eIF4G3 and ATP dependent DEAD/H helicase | 0/2 | 1/2 | eIF4G3 and ATP dependent DEAD/H helicase |
| **120** | eIF5 and hypothetical protein | 1/2 | 1/2 | eIF5 and hypothetical protein |
| **121** | Guide RNA associated complex | 1/5 | 2/5 | MRB1 associated proteins - GAP1 and 2 (49), and HslU2 protease, eIF2a and hypothetical protein |
| **122** | Complex mixture 21 | 1/3 | 2/3 | Pex7, hypothetical protein and phosphatase |
| **123** | Complex mixture 22 | 2/3 | 1/3 | 2 hypothetical proteins, one annotated as mitochondrial cytochrome bc1 component and a V-type ATPase |
| **124** | Ankyrin repeat and alanine racemase protein | 0/2 | 1/2 | Ankyrin repeat and alanine racemase protein |
| **125** | Complex mixture 23 | 1/3 | 1/3 | RPB7 RNA polymerase subunit, succinate dehydrogenase and hypothetical protein |
| **126** | Mitochondrial RNA binding complex | 1/7 | 3/7 | GBP25, GBP21, MRB1590 + tRNA uracil methyltransferase, NOL1/NOP2/sun domain, an XRN exonuclease and a hypothetical protein |
| **127** | Complex mixture 24 | 1/6 | 0/6 | Hypothetical and alpha/beta hydrolase were identified as mitochondrial ribosome components in (27),XRN exoribonuclease, a unspecified product (cAMP response gene), a mitochondrial DNA polymerase subunit and a DNA topoisomerase |
| **128** | Phosphoribosyltransferase complex | 0/8 | 3/7 | 3 PRS subunits and PRPS5 kinase, acetyl coA carboxylasem transitional ER ATPase (VCP) and phosphatidylinositol 4-phosphate 5-kinase |
| **129** | T-complex | 0/8 | 7/8 | All 8 chaperonin T-complex subunits |
| **130** | F1/F0 ATP synthase complex 2 | 0/8 | 4/8 | 6 ATP synthase subunits identified in (26) and aspartyl aminopeptidase and O-phosphoryl-rRNA selenium transferase |
| **131** | Protein phosphatase complex | 3/4 | 1/4 | Protein phosphatase 1 and 3 hypothetical proteins (1 phosphatase inhibitor, 1 translating ribosome interactor, and 1 eIF3) |
| **132** | Complex mixture 25 | 1/4 | 2/4 | A hypothetical protein, an adenylate kinase (ADKE), threonine dehydrogenase and deoxyuridine triphosphatase |
| **133** | Complex mixture 26 | 0/3 | 1/3 | Peroxidoxin, tubulin-specific chaperone and an ATP-dependent RNA helicase |
| **134** | Mitochondrial ribosome 4 | 10/18 | 4/18 | All proteins identified in (27) |
| **135** | Complex mixture 27 | 0/4 | 2/4 | seryl tRNA synthase, guanine deaminase, PFK/FBP and adenylosuccinate lyase |
| **136** | Hypothetical protein and unspecified product | 1/2 | 0/2 | RNA interference factor and iron sulfur cluster assembly protein |

| | | | | |
|---|---|---|---|---|
| **137** | Pyruvate dehydrogenase binding protein and adenylosuccinate lyase | 0/2 | 1/2 | PDH E3 binding protien and adenoylsuccinate lyase |
| **138** | Complex mixture 28 | 0/5 | 2/5 | Paraflagellar rod component, proteasome activator 26, poly(A) ribonuclease 2 and dipthamide biosynthesis enzyme |
| **139** | Hypothetical complex 9 | 3/6 | 2/6 | Glutamine synthase, polyadenylation factor, 3 hypothetical proteins and an unspecified product |
| **140** | Complex mixture 29 | 2/4 | 0/4 | 2 hypothetical proteins, an HSP20 annotated as a putative tRNA import complex and agmatinase |
| **141** | E2 ubiquitin conjugation enzyme and mago nashi protein | 0/2 | 1/2 | Exon junction component and ATG autophagy associated E2 ubiquitin conjugation enzyme |
| **142** | RuvB helicase complex and mixture 2 | 1/6 | 4/6 | 2 ruvB like DNA helicase proteins, phenylalanyl tRNA synthetase, MORN1, a hypothetical protein and glutamate dehydrogenase |
| **143** | Complex mixture 30 | 0/6 | 2/6 | amidohydrolase, pyruvate kinase, aminopeptidase, pseudouridine synthase, YjeF N-terminal domain protein and calpain like cysteine peptidase |
| **144** | Ribosome biogenesis complex | 1/16 | 9/16 | CIFTA 1,3 and 6, PUF7, and mostly ribosomal biogenesis or rRNA associated proteins |
| **145** | Complex mixture 31 | 0/3 | 1/3 | HSP110, leucine rich repeat protein and SET domain protein |
| **146** | Complex mixture 32 | 0/3 | 2/3 | Kinetoplast associated protein 3, DRBD10 and mitochondrial polymerase subunit |
| **147** | Complex mixture 33 | 1/3 | 0/3 | Bromodomain protein, glucose-6-phosphate 1-dehydrogenase and hypothetical protein |
| **148** | Complex mixture 34 | 0/4 | 0/4 | 2 retrotransposon hotspot proteins, dipthamide biosynthesis enzyme and ankyrin repeat protein |
| **149** | tRNA specific adenosine deaminase | 0/2 | 0/2 | ADAT2 and 3 |
| **150** | Hypothetical complex 10 | 3/3 | 3/3 | 3 hypothetical proteins |
| **151** | Mitochondrial tRNA methylase and membrane bound acid phosphatase | 0/2 | 1/2 | Mitochondrial tRNA methylase and membrane bound acid phosphatase |
| **152** | Complex mixture 35 | 0/6 | 1/6 | RWD domain protein, ARF like 2 binding protein, radial spoke protein 11, WD domain protein, tRNA guanine methyltransferase and ribosome interacting GTPase |
| **153** | UBA domain, diphthamide synthesis, phospanttocystein e decar, hypo | 1/4 | 0/4 | Ubiquitin like protein dipthamide biosynthesis enzyme, phosphopantothenoylysteine decarboxylase and a hypothetical protein |

| | | | | |
|---|---|---|---|---|
| **154** | Nuclear cap binding protein and peptidase | 0/2 | 0/2 | CBP110 |
| **155** | Complex mixture 36 | 0/3 | 2/3 | SET domain (putative lysine metransferase), N6-adenine methyltransferase and HSP70 |
| **156** | Queine tRNA ribosytransferase complex and mixture | 1/5 | 4/5 | 2 queine tRNA ribosyltransferase, a lysine decarboxylase, a hypothetical protein and PCNA |
| **157** | Complex mixture 37 | 1/3 | 0/3 | A hypothetical protein, aldehyde dehdrogenase and a histidine phopsphatase |
| **158** | eIF3 complex | 1/2 | 1/2 | eIF3C and hypothetical identified as eIF3A in (50) |
| **159** | tRNA dihydrouridine synthase 3 and METK1 | 0/2 | 0/2 | tRNA dihydrouridine synthase 3 and METK1 |
| **160** | Heat shock protein complex | 0/3 | 2/3 | HSP110, HSP70 and RRP45(exosome) |
| **161** | Phenylalanyl tRNA synthetase complex | 0/3 | 2/3 | Phenylalanyl tRNA synthetse alpha and beta and hslVU peptidase component |
| **162** | inositol-3-phosphate synthase and glucosamine-6-phosphate isomerase | 0/2 | 0/2 | inositol-3-phosphate synthase and glucosamine-6-phosphate isomerase |
| **163** | Complex mixture 38 | 0/5 | 1/5 | calpain peptidase, Rab GAP, Ubiquitin E1 enzyme, pyrophophatase and RHS4 |
| **164** | GPI associated complex and mixture | 0/9 | 4/9 | GPI anchor transamidase (GPI8), signal peptidase, GPI transamidase (TTA1), dolicholphosphate-mannose synthase, Ankyrin repeat protein, ISWI, IFT57/55, 3-keto-dihydrosphingosine reductase and hypothetical protein |
| **165** | Nucleolar associated complex | 1/10 | 3/10 | splicing factor (TSR1), RSBR1 RNA binding protein, 2 arginine methyltransferases (functional association from personal communication with Laurie Read), NOP1 (fibrillarin) NOP56, nucleolar RNA bind, cytosine tRNA metransferase, fibrillarin, a hypothetical protein |
| **166** | Complex mixture 39 | 2/5 | 0/5 | Serine peptidase, 2 hypothetical proteins, disulfide isomerase, and ALG9 mannosyltransferase |
| **167** | Putative Golgi/ER biogenesis complex | 0/2 | 0/2 | NSF attachment protein (SNARE type) and SEP containing protein with NSFL1 cofactor Blast |
| **168** | Trans-sialidase and ubiquitin like domain protein | 0/2 | 0/2 | Trans-sialidase and ubiquitin like domain protein |
| **169** | FE(II) oxygenase and hypothetical protein | 1/2 | 0/2 | Hypothetical protein annotate as FLA1BP |

| | | | | |
|---|---|---|---|---|
| **170** | NADH fumarate reductase and pyrroline-5-carboxylate reductase | 0/2 | 1/2 | NADH fumarate reductase and pyrroline-5-carboxylate reductase |
| **171** | Hypothetical complex 11 | 2/2 | 1/2 | 2 hypothetical proteins |
| **172** | Arginine N-metrans (PRMT7) and nascent polypeptide associated complex protein | 0/2 | 1/2 | PRMT7 and dubious annotation of nascent polypeptide associated complex protein |
| **173** | Hypothetical protein and deoxyribose-phosphate aldolase | 1/2 | 0/2 | Hypothetical protein and deoxyribose-phosphate aldolase |
| **174** | CNOT 10 and 11 complex | 1/2 | 2/2 | Hypothetical protein, human homolog CNOT11 and CNOT11 protein. CNOT10 and 11 known to interact in human (32) |
| **175** | Helicase and ubiquitin ligase | 0/2 | 0/2 | Ubiquitin ligase also annotated as yeast TOM1 homolog with role in nuclear mRNA export |
| **176** | DRBD2 and RHS4 | 0/2 | 0/2 | DRBD2 and RHS4 |
| **177** | Unspecified product and fatty acyl CoA synthetase 2 | 0/2 | 1/2 | Unspecified product annotated as ribosomal protein |
| **178** | Peroxisome biogenesis associated complex | 0/2 | 0/2 | Peroxisome biogenesis protein and assembly protein PEX1 and 6 |
| **179** | eIF3L and COP9 signalosome (eIF3K) and tatD deoxyribonuclease | 0/3 | 1/3 | COP9 signalsome protein annotated as eIF3K (50) |
| **180** | U5 spliceosome complex | 0/3 | 2/3 | U5 spliceosome protein, PRP8 and ATP dependent RNA helicase with hoology to U5 spliceosome component. All identified in pull down experimetn of U5 spliceosome in (51) |
| **181** | Complex mixture 40 | 1/3 | 0/3 | E2 ubiquitin conjugating enzyme, hypothetical protein and eIF1A |
| **182** | Complex mixture 41 | 1/4 | 3/4 | hypo, MEX67 mRNA export, kinetoplastid phosphatase, SEC7 |
| **183** | Complex mixture 42 | 1/4 | 0/4 | hypothetical protein, 3-hydroxy-3methyl glutaryl coA reductase, endonuclease, enoyl CoA hydratase |
| **184** | Complex mixture 43 | 1/4 | 2/4 | hypothetical protein, snoRNA binder, Dpy-30 domain protein and SEC13 |
| **185** | Importin alpha and M2 dsRNA replication associated protein | 0/2 | 2/2 | Importin annotated as Kap60 and M2 dsRNA protein is annotated as putative splicing protein associated with nuclear speckles |
| **186** | Cleavage and poly(A) | 0/2 | 1/2 | 2 cleavage and poly(A) specficity factor proteins, CPSF2 and 3 |

| | | | | |
|---|---|---|---|---|
| | specificity complex | | | |
| **187** | Paraflagellar rod complex | 0/2 | 0/2 | PFC4 and 16 |
| **188** | DUF complex | 0/2 | 0/2 | two DUF proteins with same domain (DUF2009) |
| **189** | Coatomer complex and mixture | 1/6 | 4/6 | Coatomer beta and gamma, together with PFC5, SRP54, hypothetical protein and NUP93 |
| **190** | Complex mixture 44 | 0/3 | 1/3 | PGI, 6-phosphogluconate dehydrogenase, citrate synthase |
| **191** | NOP86 and cytoskeleon associated protein | 0/2 | 2/2 | NOP86 and cytoskeleon associated protein |
| **192** | Complex mixture 45 | 1/5 | 0/5 | 2 methyltranferase domain containing proteins, a hypothetical protein, monthiol glutaredoxin and a cysteine peptidase |
| **193** | Acyl carrier protein and lipoic acid carrier protein | 0/2 | 0/2 | ACP, member of NADHubiquinone oxidoreductase complex and lipoic acid carrier protein, GCVH |
| **194** | Electron transfer flavoprotein complex | 0/2 | 0/2 | 2 electron transfer flavoproteins |
| **195** | Hydroxy-meglutaryl-coA lyase and NAD+ synthase | 0/2 | 1/2 | Hydroxy-meglutaryl-coA lyase and NAD+ synthase |
| **196** | D-alanine D-alanine ligase and N2227 protein | 0/2 | 1/2 | D-alanine D-alanine ligase and N2227 protein |
| **197** | Inositol-1 monophosphatase complex | 0/2 | 0/2 | 2 IMPases |
| **198** | Ubiquitin E2 conjugation enzyme and hypothetical protein | 1/2 | 2/2 | Ubiquitin E2 conjugation enzyme and hypothetical protein |
| **199** | Hypothetical protein and S/T kinase NrkA | 1/2 | 1/2 | Hypothetical protein and S/T kinase NrkA |
| **200** | RGG domain protein and adenine phosphoribosyltransferase | 0/2 | 1/2 | RGG domain protein and adenine phosphoribosyltransferase |
| **201** | maoC dehydratase and DJ-1 family protein | 0/2 | 1/2 | maoC dehydratase and DJ-1 family protein |
| **202** | ras-like GTPase complex | 0/2 | 0/2 | 2 ras GTPases, GTR and GTP |
| **203** | Ubiquitin conjugation complex | 0/2 | 0/2 | 2 E2 ubiqutin conjugation enzymes |
| **204** | ADP ribosylation factor and tryparedoxin 1a | 0/2 | 1/2 | ADP ribosylation factor and tryparedoxin 1a |

| | | | | |
|---|---|---|---|---|
| **205** | Farnesyltransferase complex | 0/3 | 2/3 | Farnesyltranfserase alpha and beta subunits and PARN2 |
| **206** | Glutathione synthetase and ankyrin repeat protein | 0/2 | 0/2 | Glutathione synthetase and ankyrin repeat protein |
| **207** | Nuclear pore complex | 0/3 | 2/3 | NUP155, Nup93 and POMP1 a mitochondrial outer membrane protein |
| **208** | N-acetyltransferase complex | 0/3 | 1/3 | N-acetyltransferases ARD1 and NatB and ribonucleoside-diphosphate reductase RNR1 |
| **209** | Tryparedoxin-peroxidase complex | 0/2 | 1/2 | TDPX1 and 2 |
| **210** | RAB11 and translation machinery associated protein | 0/2 | 1/2 | RAB11 and translation machinery associated protein |
| **211** | Glutamine amidotransferase and choline kinase | 0/2 | 1/2 | Glutamine amidotransferase and choline kinase |
| **212** | Fatty acid CoA ligase and enoyl CoA hydratase | 0/2 | 0/2 | Fatty acid CoA ligase and enoyl CoA hydratase |
| **213** | Complex mixture 46 | 1/5 | 1/5 | Hypothetical protein, SET domain protein, MAPK4, homocysteineSmethyltransferase and ornithine decarboxylase |
| **214** | malic enzymes | 0/2 | 0/2 | 2 malic enzyme proteins |
| **215** | Complex mixture 47 | 0/4 | 3/4 | ribose 5-phosphate isomerase, spermidine synthase, uracil phosphoribosyltransferase, PGAM |
| **216** | Valyl tRNA synthetase and METK1 | 0/2 | 0/2 | Valyl tRNA synthetase and METK1 |
| **217** | glyceraldehyde-3-ph DH, NGG1, SET dom, serine peptidase and PLP G decarboxylase | 0/5 | 1/5 | Glyceraldehyde-3-phosphate dehydrogenase, NGG1 interacting factor like protein, SET domain protein, serine peptidase and pyridoxal phosphate containing glycine decarboxylase |
| **218** | Complex mixture 48 | 0/3 | 1/3 | Fatty acyl coA synthase (ACS1), proline dehydrogenase and sterol alpha demethylase |
| **219** | Mitochondrial processing peptidase complex | 0/3 | 1/3 | Mitochondrial processing peptidase alpha and beta subunits shown to interact in (52) and an alpha beta hydrolase |
| **220** | Metallopeptidase and ubiquitin activating enzyme | 0/2 | 1/2 | Metallopeptidase and E1 ubiqutin activating enzyme UBA1 |
| **221** | S/T protein phosase 2A and T complex like protein | 0/2 | 1/2 | S/T protein phosase 2A and T complex like protein |
| **222** | Complex mixture 49 | 0/3 | 0/3 | aminopeptidase, alanine aminotransferase and aspartate aminotransferase |

| | | | | |
|---|---|---|---|---|
| **223** | cytosolic malate dehydrogenase and uridine phosphorylase | 0/2 | 0/2 | cytosolic malate dehydrogenase and uridine phosphorylase |
| **224** | Complex mixture 50 | 0/4 | 0/4 | GMP synthase, branched chain amino acid aminotransferase, phosphoacetylglucosamine mutase, methylglutaconyl-coA hydratase |
| **225** | Complex mixture 51 | 0/3 | 1/3 | UTP-glucose-1-phosphate uridyltransferase, nitrilase, MAPKKK |
| **226** | Complex mixture 52 | 0/3 | 2/3 | adenosine kinase, transaldolase and enoyl CoA hydratase |
| **227** | adenine phribosyltrans, diO2ase, iron superoxide dismutase | 0/3 | 0/3 | adenine phosphoribosyltransferase, dioxygenase, iron superoxide dismutase |
| **228** | Kynureninase and aminopeptidase | 0/2 | 1/2 | Kynureninase and aminopeptidase |
| **229** | Phosphatase and metalloprotease | 0/2 | 0/2 | Phosphatase and metalloprotease |
| **230** | Trypanothione reductase and glutamine aminotransferase | 0/2 | 0/2 | Trypanothione reductase and glutamine aminotransferase |
| **231** | Complex mixture 53 | 0/3 | 0/3 | Pyridoxal kinase, nucleosidehydrolase, dihydroorotate oxidase |
| **232** | 14-3-3 protein complex | 0/2 | 2/2 | 14-3-3 subunits I and II, identified in (53) |
| **233** | Mito ADP/ATP carrier protein and voltage-dependent anion-selective channel | 0/2 | 0/2 | MCP5a and VDAC2 |
| **234** | Complex mixture 54 | 1/3 | 1/3 | Hypothetical protein, tatD deoxyribonuclease and MAPK3 |

**Supp Table 4. List of proteins identified in Complex 31 – proteasome core complex.**

| Gene ID | GeneDB Annotation | BLAST search | Interpro domain search |
|---|---|---|---|
| **Tb11.v5.0196** | Proteasome subunit A N-terminal signature/Proteasome subunit, putative | 20S proteasome subunit alpha-6 (2.5e-141) | Proteasome subunit A N-terminal signature (IPR000426, 1.3e-13); Proteasome subunit (IPR001353, 1.4e-53) |
| **Tb927.10.230** | proteasome subunit alpha type-5, putative | 20S proteasome subunit alpha-5 (3.6e-126) | Proteasome subunit A N-terminal signature (IPR000426, 2e-13); Proteasome subunit (IPR001353, 6.7e-57) |
| **Tb927.10.290** | proteasome alpha 2 subunit, putative | 20S proteasome subunit alpha-2 (3.5e-121) | Proteasome subunit A N-terminal signature (IPR000426, 1.5e-9); Proteasome subunit (IPR001353, 2.7e-63) |
| **Tb927.10.4710** | 20S proteasome subunit, proteasome subunit beta type-2, putative (PSB4) | 20S proteasome subunit beta-4 (6.8e-109) | Proteasome subunit (IPR001353, 1e-27) |
| **Tb927.10.6080** | proteasome subunit beta type-5, putative, proteasome subunit beta type-5, putative | Proteasome subunit beta type-5 (2.8e-71) | Proteasome subunit (IPR001353, 2.2e-50) |

| | | | |
|---|---|---|---|
| **Tb927.11.7020** | proteasome alpha 7 subunit, putative (PSA4) | 20S proteasome subunit alpha-4 (5.1e-127) | Proteasome subunit A N-terminal signature (IPR000426, 1.3e-14); Proteasome subunit (IPR001353, 4.8e-58) |
| **Tb927.11.7270** | proteasome beta 3 subunit, putative (PSB3) | 20S proteasome subunit beta-3 (5.5e-107) | Proteasome subunit (IPR001353, 1e-39) |
| **Tb927.3.780** | proteasome alpha 7 subunit (TbPSA7) | 20S proteasome subunit alpha-7 (1.6e-59) | Proteasome subunit A N-terminal signature (IPR000426, 5.7e-11); Proteasome subunit (IPR001353, 5.6e-44) |
| **Tb927.4.430** | proteasome beta 7 subunit | Proteasome subunit beta type-4 (1.2e-40) | Proteasome subunit (IPR001353, 7.2e-36) |
| **Tb927.6.1260** | proteasome beta-1 subunit, putative (PSB1) | Proteasome subunit beta type-6 (8.6e-54) | Proteasome subunit (IPR001353, 1e-42) |
| **Tb927.7.4420** | proteasome alpha 3 subunit, putative | 20S proteasome subunit alpha-3 (4.5e-64) | Proteasome subunit A N-terminal signature (IPR000426, 1.3e-12); Proteasome subunit (IPR001353, 6.4e-48) |
| **Tb927.7.4790** | proteasome beta 6 subunit, 20S proteasome beta 6 subunit, putative (BETA6) | 20S proteasome subunit beta-6 (2.6e-139) | Proteasome subunit (IPR001353, 2.2e-32) |
| **Tb927.9.11310** | unspecified product | Proteasome subunit beta type-2 (2.7e-73) | Proteasome subunit (IPR001353, 8.9e-47) |
| **Tb927.9.9670** | proteasome alpha 1 subunit, putative, 20S proteasome subunit alpha-6, (putative) (TbPSA6) | Proteasome subunit alpha type-1 (4e-120) | Proteasome subunit A N-terminal signature (IPR000426, 1.2e-11); Proteasome subunit (IPR001353, 1.7e-38) |

17 **Supp Table 5. List of proteins identified in Complex 130 - $F_0F_1$-ATP synthase complex.**

| Gene ID | GeneDB Annotation | Homology search | BLAST search | Interpro domain search |
|---|---|---|---|---|
| **Tb927.10.180** | ATP synthase F1 subunit gamma protein, putative | ATP synthase gamma chain (2.1e-20) | ATP synthase (IPR000131, 4.7e-33); ATP synthase (F1-ATPase), gamma subunit (IPR023633, 4.5e-49) | Classed as gamma subunit of $F_0F_1$ ATP synthase in (26) |
| **Tb927.10.5050** | Mitochondrial ATP synthase epsilon chain, putative | - | Epsilon subunit of mitochondrial F1F0-ATP synthase (IPR006721, 9.7e-13) | Classed as epsilon subunit of $F_0F_1$ ATP synthase in (26) |
| **Tb927.11.13070** | O-phosphoseryl-tRNA(Sec) selenium transferase, putative | Selenocysteinyl-tRNA(Sec) synthase (9.4e-100) | Soluble liver antigen/liver pancreas antigen (IPR008829, 9.2e-59) | - |
| **Tb927.3.1380** | ATP synthase subunit beta, mitochondrial, ATP synthase F1, beta subunit (ATPB) | ATP synthase subunit beta (1.3e-171) | ATP synthase alpha/beta family, nucleotide-binding domain (IPR000194, 5.7e-64) | Classed as beta subunit of $F_0F_1$ ATP synthase in (26) |
| **Tb927.3.3410** | aspartyl aminopeptidase, putative, metallo-peptidase, Clan MH, Family M20 | Aspartyl aminopeptidase (3.3e-111) | - | - |

| | | | | |
|---|---|---|---|---|
| **Tb927.5.1710** | ribonucleoprotein p18, mitochondrial precursor, putative | Protein P18 (1.4e-76) | - | Classed as b subunit of $F_0F_1$ ATP synthase in (26) |
| **Tb927.6.4990** | ATP synthase, epsilon chain, putative | ATP synthase subunit delta (6.7e-15) | ATP synthase, Delta/Epsilon chain, beta-sandwich domain (IPR020546, 3.1e-17) | Classed as delta subunit of $F_0F_1$ ATP synthase in (26) |
| **Tb927.7.7430** | ATP synthase alpha chain, mitochondrial precursor, ATP synthase F1, alpha subunit | ATP synthase subunit alpha (1.3e-107) | ATP synthase alpha/beta family, nucleotide-binding domain (IPR000194, 4.8e-69) | Classed as alpha subunit of $F_0F_1$ ATP synthase in (26) |

**Supp Table 6. List of proteins identified in Complex 85 - $F_0F_1$-ATP synthase complex.**

| Gene ID | GeneDB Annotation | BLAST search | Interpro domain search | Comments |
|---|---|---|---|---|
| **Tb927.10.8030** | hypothetical protein, conserved | - | ATP synthase delta (OSCP) subunit (IPR000711, 2.6e-5) | Classed as OSCP subunit of $F_0F_1$ ATP synthase in (26) |
| **Tb927.11.6250** | hypothetical protein, conserved | - | - | Classed as trypanosome specific subunit of $F_0F_1$ ATP synthase in (26) |
| **Tb927.5.1780** | hypothetical protein, conserved | - | - | - |

**Supp Table 7. List of proteins identified in Complex 4 – mitochondrial ribosome.**

| Gene ID | GeneDB Annotation | BLAST search | Interpro domain search | Comments |
|---|---|---|---|---|
| **Tb927.11.12930** | DEAD-box helicase, putative | ATP-dependent rRNA helicase RRP3 (2e-30) | DEAD/DEAH box helicase (IPR011545, 3.8e-26) | - |
| **Tb927.11.5990** | hypothetical protein, conserved | Pseudouridylate synthase 7 homolog-like protein (6.9e-9) | Pseudouridine synthase (IPR020103, 8.8e-35) | mitochondrial LSU protein (27) |
| **Tb927.4.1070** | 50S ribosomal protein L13, putative | 50S ribosomal protein L13 (6.5e-17) | Ribosomal protein L13 (IPR023564, 2e-30) | MRPL13 (27) |
| **Tb927.6.4080** | hypothetical protein, conserved | - | - | mitochondrial SSU or LSU protein (27) |
| **Tb927.7.1640** | ras-like small GTPase, putative (TbEAR) | GTPase Der (1.5e-56) | 50S ribosome-binding GTPase (IPR006073, 1.8e-15) | mitochondrial LSU protein (27) |
| **Tb927.7.3460** | hypothetical protein, conserved | - | - | mitochondrial LSU protein, KRIT2 (27) |

**Supp Table 8. List of proteins identified in Complex 92 – mitochondrial ribosome.**

| Gene ID | GeneDB Annotation | BLAST search | Interpro domain search | Comments |
|---|---|---|---|---|

| | | | | |
|---|---|---|---|---|
| **Tb927.10.3580** | hypothetical protein, conserved | - | - | mitochondrial SSU protein (27) |
| **Tb927.11.2530** | Mitochondrial SSU ribosomal protein, putative | - | - | mitochondrial SSU protein (27) |
| **Tb927.7.3050** | hypothetical protein, conserved | - | - | mitochondrial SSU protein (27) |

**Supp Table 9. List of proteins identified in Complex 134 – mitochondrial ribosome.**

| Gene ID | GeneDB Annotation | BLAST search | Interpro domain search | Comments |
|---|---|---|---|---|
| **Tb927.1.1200** | SSU ribosomal protein, mitochondrial (MRPS15) | - | S15/NS1 RNA-binding domain (IPR009068, 1.9e-7) | MRPS15 (27) |
| **Tb927.10.7380** | hypothetical protein, conserved | - | - | mitochondrial LSU protein (27) |
| **Tb927.11.11630** | hypothetical protein, conserved | - | - | mitochondrial LSU protein (27) |
| **Tb927.11.1250** | Mitochondrial SSU ribosomal protein, putative | - | - | mitochondrial SSU protein (27) |
| **Tb927.11.6000** | ribosomal protein L4/L1 family, putative | 50S ribosomal protein L4 (1.3e-6) | Ribosomal protein L4 (IPR023574, 4.6e-40) | MRPL4 (27) |
| **Tb927.11.870** | hypothetical protein, conserved | - | Ribosomal proteins S24e, L23 and L15e (IPR012678, 7.6e-9) | MRPL23 (27) |
| **Tb927.2.4890** | ribosomal protein L11, putative | 54S ribosomal protein L19, mitochondrial (1.3e-13) | Ribosomal protein L11/L12 (IPR000911, 1.2e-9) | MRPL11 (27) |
| **Tb927.3.5610** | ribosomal protein L3 mitochondrial, putative | 39S ribosomal protein L3, mitochondrial (2e-27) | Ribosomal protein L3 (IPR000597, 1.2e-11) | MRPL3 (27) |
| **Tb927.5.3980** | hypothetical protein, conserved | 54S ribosomal protein L10, mitochondrial (1.9e-8) | Ribosomal proteins L15p and L18e (IPR021131, 3.1e-9) | MRPL15 (27) |
| **Tb927.5.4120** | hypothetical protein, conserved | - | - | mitochondrial LSU protein (27) |
| **Tb927.6.2080** | hypothetical protein, conserved | - | - | mitochondrial SSU protein (Zikova 2008) |
| **Tb927.6.4560** | hypothetical protein, conserved | - | - | mitochondrial SSU protein (27) |
| **Tb927.7.2760** | ribosomal protein L22p/L17e, putative | 50S ribosomal protein L22 (7.6e-9) | Ribosomal protein L22 (IPR001063, 6.4e-20) | MRPL22 (27) |
| **Tb927.7.3030** | hypothetical protein, conserved | - | - | |
| **Tb927.7.4140** | ribosomal protein L21, putative | Probable 39S ribosomal protein L21, mitochondrial (9.8e-14) | Ribosomal prokaryotic L21 protein (IPR001787, 7e-20) | MRPL21 (27) |
| **Tb927.8.5200** | hypothetical protein, conserved | - | - | mitochondrial SSU protein (27) |

| | | | | |
|---|---|---|---|---|
| **Tb927.9.7170** | Mitochondrial 39-S ribosomal protein L47 (MRP-L47), putative | 39S ribosomal protein L47, mitochondrial (1.2e-13) | Mitochondrial 39-S ribosomal protein L47 (IPR010729, 2.3e-25) | MRPL47 (27) |
| **Tb927.9.8290** | hypothetical protein, conserved | - | Ribosomal protein L30p/L7e (IPR016082, 9.9e-12) | MRPL30 (27) |

**Supp Table 10. List of proteins identified in Complex 3 – AMPK.**

| Gene ID | GeneDB Annotation | BLAST search | Interpro domain search | Comments |
|---|---|---|---|---|
| **Tb927.10.3700** | AMP-activated protein kinase, gamma regulatory subunit, SNF1-related protein kinase regulatory subunit gamma, AMPK subunit gamma (AMPKG) | AMPK subunit gamma-3 (2.2e-14) | - | identified in (28) |
| **Tb927.10.5310** | SNF1-related protein kinases, putative | AMPK subunit alpha-1 (3.1e-77) | - | - |
| **Tb927.3.4560** | 5'-AMP-activated protein kinase catalytic subunit alpha, putative, AMPK subunit alpha, putative, SNF1-related protein kinase catalytic subunit alpha, putative | AMPK subunit alpha-2 (4.2e-90) | - | - |
| **Tb927.8.2450** | SNF1-related protein kinase regulatory subunit beta, 5'-AMP-activated protein kinase subunit beta, AMPK subunit beta (AMPKB) | AMPK subunit beta-1 (1.1e-10) | 5'-AMP-activated protein kinase beta subunit, interation domain (IPR006828, 9.6e-41) | identified in (28) |
| **Tb927.9.9270** | hypothetical protein, conserved | Ankyrin repeat and zinc finger domain-containing protein 1 (1.6e-12) | - | - |

**Supp Table 11. List of proteins identified in Complex 72 – PUF10 complex.**

| Gene ID | GeneDB Annotation | BLAST search | Interpro domain search | Comments |
|---|---|---|---|---|
| **Tb927.11.6740** | pumilio/PUF RNA binding protein 10, putative | - | ARM repeat (IPR016024, 2.4e-19) | mRNA binding (29) |
| **Tb927.7.2170** | hypothetical protein, conserved | ANP32/acidic nuclear phosphoprotein-like protein 2 (3.3e-10) | - | mRNA binding (29) |

**Supp Table 12. List of proteins identified in Complex 174 – CNOT 10 and 11 complex.**

| Gene ID | GeneDB Annotation | BLAST search | Interpro domain search | Comments |
|---|---|---|---|---|
| **Tb927.10.8720** | CCR4-NOT transcription complex subunit 10, putative | - | - | Identified in Caf1 pulldown (30) |

| | | | | |
|---|---|---|---|---|
| **Tb927.8.1960** | hypothetical protein, conserved | - | - | Copurified with Caf1 protein of CNOT complex, and Tb927.10.8720 and is human homolog of CNOT11 (30-32) |

**Supp Table 13. List of proteins identified in Complex 108 – U3 ribonucleoprotein.**

| Gene ID | GeneDB Annotation | BLAST search | Interpro domain search |
|---|---|---|---|
| **Tb927.10.13270** | Periodic tryptophan protein 2 homolog, putative | Periodic tryptophan protein 2 homolog (2.2e-116) | WD40 repeat-like (IPR017986, 2.9e-52) |
| **Tb927.11.10480** | PQQ-like domain/WD domain, G-beta repeat/Utp21 specific WD40 associated putative domain containing protein, putative | U3 snoRNA-associated protein 21 (2.9e-21) | Utp21 specific WD40 associated putative domain (IPR007319, 4e-19) |
| **Tb927.11.460** | predicted WD40 repeat protein | U3 snoRNA-associated protein 13 (2.2e-51) | Utp13 specific WD40 associated domain (IPR013934, 2.3e-42) |
| **Tb927.7.4220** | WD domain, G-beta repeat/Dip2/Utp12 Family, putative | U3 snoRNA-associated protein 12 (1.6e-54) | Dip2/Utp12 Family (IPR007148, 2.6e-6) |

**Supp Table 14. List of proteins identified in Complex 76 – H/ACA ribonucleoprotein.**

| Gene ID | GeneDB Annotation | BLAST search | Interpro domain search |
|---|---|---|---|
| **Tb927.10.170** | pseudouridine synthase, Cbf5p | H/ACA ribonucleoprotein complex subunit 4 (9.6e-142) | - |
| **Tb927.4.470** | snoRNP protein GAR1, putative | snoRNP protein GAR1 (8.6e-54) | Gar1/Naf1 RNA binding region (IPR007504, 1.4e-38) |
| **Tb927.4.750** | 50S ribosomal protein L7Ae, putative | snoRNP protein NHP2 (4e-24) | Ribosomal protein L7Ae/L30e/S12e/Gadd45 family (IPR004038, 2.1e-17) |

**Supp Table 15. List of proteins identified in Complex 12 – HSP70/90 complex.**

| Gene ID | GeneDB Annotation | Homology search | BLAST search | Interpro domain search |
|---|---|---|---|---|
| **Tb927.10.13670** | serine/threonine protein phosphatase 5 | Serine/threonine-protein phosphatase 5 (3.2e-132) | - | interacts with HSP90 (35) |
| **Tb927.10.14030** | hypothetical protein, conserved | - | - | - |
| **Tb927.3.3580** | heat shock protein 90, putative (LPG3) | HSP90.2 (7.8e-132) | ATPase domain of HSP90 chaperone/DNA topoisomerase II/histidine kinase (IPR003594, 8.6e-61) | - |
| **Tb927.5.3260** | WD domain, G-beta repeat, putative | WD repeat-containing protein 17 (4.7e-58) | WD40 repeat-like (IPR017986, 2.6e-46) | - |
| **Tb927.9.9860** | Hsp70 protein, putative | Hsp70-17 (8.6e-68) | Hsp70 protein (IPR013126, 1.6e-67) | - |

**Supp Table 16. List of proteins identified in Complex 99 – MTR1 and eIFB complex.**

| Gene ID | GeneDB Annotation | BLAST search | Interpro domain search | Comments |
|---|---|---|---|---|
| **Tb927.10.5840** | translation elongation factor 1-beta, putative | EF-1-beta (1.8e-62) | eEF-1beta-like (IPR014038, 7.7e-30) | - |
| **Tb927.10.7940** | methyltransferase, putative | MTr1 (4.8e-202) | FtsJ-like methyltransferase (IPR002877, 1.3e-30) | - |
| **Tb927.11.13190** | elongation factor 1 gamma, putative | eEF-1B gamma (8.3e-175) | eEF1-gamma domain (IPR001662, 8.4e-63) | - |
| **Tb927.11.16490** | hypothetical protein, conserved | Telomerase Cajal body protein 1 (2.4e-9) | WD40 repeat-like (IPR017986, 1.6e-14) | Identified interacting with Mtr1 in (37) |
| **Tb927.4.3590** | translation elongation factor 1-beta, putative | EF-1-beta (3.3e-84) | eEF-1beta-like (IPR014038, 1.3e-29) | - |

**Supp Table 17. List of proteins identified in Complex 165 – nucleolar complex.**

| Gene ID | GeneDB Annotation | BLAST search | Interpro domain search |
|---|---|---|---|
| **Tb927.10.11310** | intraflagellar transport protein 57/55 (IFT57/55) | Intraflagellar transport protein 57 homolog (7.7e-53) | Intra-flagellar transport protein 57 (IPR019530, 8.3e-125) |
| **Tb927.10.13860** | GPI-anchor transamidase subunit 8 (GPI8) | GPI transamidase (1.8e-51) | Peptidase C13 family (IPR001096, 1.1e-31) |
| **Tb927.10.4040** | 3-keto-dihydrosphingosine reductase | 3-ketodihydrosphingosine reductase (1.9e-33) | short chain dehydrogenase (IPR002198, 6.7e-35) |
| **Tb927.10.4610** | dolicholphosphate-mannose synthase, putative (DPMS) | Dolichol-phosphate mannosyltransferase (2e-61) | Glycosyl transferase family 2 (IPR001173, 4.3e-33) |
| **Tb927.11.13820** | hypothetical protein, conserved | - | - |
| **Tb927.11.15760** | GPI transamidase subunit Tta1 (TTA1) | - | - |
| **Tb927.2.1810** | transcription silencer (ISWI) | ISW2-like (1.4e-168) | SNF2 family N-terminal domain (IPR000330, 1.2e-82) |
| **Tb927.5.1930** | signal peptidase subunit, putative | - | Signal peptidase subunit (IPR007653, 5.9e-13) |
| **Tb927.8.5760** | Ankyrin repeats (many copies)/Alpha/beta hydrolase family, putative | - | - |

**Supp Table 18. List of proteins identified in Complex 164 – GPI associated complex.**

| Gene ID | GeneDB Annotation | BLAST search | Interpro domain search |
|---|---|---|---|
| **Tb927.1.4690** | arginine N-methyltransferase (PRMT1) | Histone-arginine N-methyltransferase PRMT1 (7.7e-85) | - |
| **Tb927.10.12980** | Multisite-specific tRNA:(cytosine-C(5))-methyltransferase, putative | tRNA (cytosine-5-)-methyltransferase NCL1 (1.5e-38) | NOL1/NOP2/sun family (IPR001678, 9.2e-21) |
| **Tb927.10.14750** | fibrillarin, putative | rRNA 2-X-methyltransferase fibrillarin (2e-102) | Fibrillarin (IPR000692, 6.7e-103) |
| **Tb927.10.1960** | hypothetical protein, conserved | - | ARM repeat (IPR016024, 1.5e-13) |

| | | | |
|---|---|---|---|
| **Tb927.10.3560** | arginine N-methyltransferase, putative | Probable protein arginine N-methyltransferase 1 (4.8e-37) | - |
| **Tb927.10.7500** | fibrillarin (NOP1) | rRNA 2-X-methyltransferase fibrillarin (4.2e-132) | Fibrillarin (IPR000692, 2.2e-106) |
| **Tb927.8.3750** | Nucleolar protein 56, putative (NOP56) | Nucleolar protein 56 (2.1e-107) | Putative snoRNA binding domain (IPR002687, 4.1e-56) |
| **Tb927.8.900** | splicing factor TSR1 (TSR1) | Splicing factor, arginine/serine-rich 2 (1.7e-18) | - |
| **Tb927.9.5320** | nucleolar RNA binding protein, putative | Nucleolar protein 58-2 (6.5e-100) | Putative snoRNA binding domain (IPR002687, 1.6e-52) |
| **Tb927.9.6870** | RNA-binding protein, putative (RBSR1) | Probable splicing factor, arginine/serine-rich 6 (7.2e-18) | - |

**Supp Table 19. Single peptide identification reports.**

For each experiment type (SEC300, SEC1000 and SAX) the table reports the proteins identified with one single peptide (-1) or not identified (0) in each biological replicate of the SEC 300 (E3017, E3019, E3021, E3023) and SEC100 (E3020, E3024 E3027) or in the SAX experiment (E3028). Each biological replicate and the SAX experiment contain several fractions. For this reason, the count of the unique peptide bigger than one reports only one value. This value is selected from the fraction of the biological replicates that shows the maximum number of unique peptides.

**Supp Table 20. Identified protein complex report.**

The table lists the id of the predicted protein complex (id_predicted) and the number of proteins that belong to the predicted complex (len_predicted). The table further reports the name (name_gd) and the number of components of the gold standard protein complex that is most similar to the predicted protein complex. Finally, the table reports the protein in common (len_common) between the predicted complex and the gold standard complex with the number of proteins of the gold standard complex that were missed from the prediction (missed_component).

**Supp Table 21. Single peptide identification reports.**

The excel file collects 101 tables, one table with the positive pairs and 100 tables with the random selection of negative pairs. Each table reports the protein ids (prot_1 and prot_2) and the protein descriptions (desc_1 and desc_2) of the protein pairs.

**Supp Table 22. MaxQuant results**

LFQ quantitation results for the SEC300, SEC1000 and SAX experiments. For each identification, the table reports the TriTrypDB accession number (Protein ID), the number of distinct peptides assigned for each protein (Peptides), the percentage coverage of each protein assigned (Sequence converage [%]) anc the LFQ quantification measurements for each analysed fraction (LFQ intensity).

# Supplementary Figure Captions

**Supp Fig 1. Characterisation of Size Exclusion Chromatography columns.**

Panels (A) and (B) chromatograms of 280 nm absorbance of indicated molecular weight markers (coloured lines) or *Trypanosoma brucei* lysates (black line) loaded onto an SEC300 or SEC1000 column respectively. (C) Linear regression of molecular weight markers and retention time on SEC300 column. (D) SDS-PAGE of pooled fractions collected from SEC300 fractionation of *T. brucei* lysates in (i) PBS or in an (ii) SDS containing buffer.

**Supp Fig 2. Characterisation of Strong Anion Exchange column.**

Chromatogram of 280 nm absorbance of individual proteins (coloured lines) or *Trypanosoma brucei* lysate (black line) loaded onto an SAX column.

**Supp Fig 3. Reproducibility of SEC300 and SEC1000 fractionation.**

(A) Median Pearson correlation across experiments between collected fractions. (B) Elution profiles of three representative proteins across five replicates of SEC300 or SEC1000 experiments. (C) Median Pearson correlation of elution profiles across biological replicates using either iBAQ, LFQ, MS/MS counts or unique peptide counts as metrics for protein quantitation.

**Supp Fig 4. Machine learning output.**

(A) Frequency of interaction prediction score values for the positive (green) and negative (red) protein interaction datasets. (B) False Positive Rate of protein interaction prediction reported across a range of interaction prediction score thresholds. (C) Receiver operator curves for random forest predictor 1 or 2 across 100 iterations with different sets of true negative interactions. (D) Feature importance output for each random forest predictor,

demonstrating the utility of each scoring feature for predicting gold standard interactions (Supplementary Table 1).

**Supp Fig 5. Frequency of number of subunits within protein complexes predicted by machine learning.**

Distribution of the number of proteins present within each protein complex predicted by machine learning.

**Supp Fig 6. Elution profiles of novel predicted complexes.**

Elution profiles of complexes (A) 72, (B) 174, (C) 108 and (D) 76, predicted from machine learning analysis. These predicted complexes contain novel predictions of interaction between proteins of unknown, or poorly characterised function.

**Supp Fig 7. Elution profiles of novel interactions between characterised proteins.**

Elution profiles of complexes (A) 12, (B) 99, (C) 165 and (D) 164, predicted from machine learning analysis. These predicted complexes demonstrate previously uncharacterised putative interactions between proteins of known function.

**Supp Fig 8. Evaluation of hierarchical clustering and machine learning methods.**

Mean value of precision (A) and recall (B) for the prediction of gold standard complexes (Supplementary Table 1) using either the machine learning pipeline (Machine Learning), or hierarchical clustering of SEC300, SEC1000 or SAX data (left hand panels). The distribution of precision (A) and recall (B) scores for all identified gold standard complexes are displayed as a kernel distribution plot (right hand panels).

**Supp Fig 9. Comparison to previous publications.**

(A) The number of shared (Common) and unique protein complexes predicted in this work (Unique in this work) and in (19) (Unique in PLoS). The mean Pearson correlation coefficient of elution profiles within complexes are reported in the table below, for each dataset from work published here (SEC300, SEC1000 and SAX) and from (19) (IEX-cyto, IEX-mito, GG-WCL and GG-mito). (B) The probability (Frequency) of a random protein pair (Counts) elution profile with a Pearson correlation coefficient >0.7 is plotted for the SAX data produced in the work published here, and the IEX-cyto experiment from (19).

**Supp Fig 10. Data visualisation tool – Profile Explorer.**

Interactive web application which allows users to search for and display all elution profiles characterised in this manuscript and in (19), and compare up to twenty proteins to assess their potential interaction.

**Supp Fig 11. Data visualisation tool – Cluster Explorer.**

Interactive web application to browse lower confidence protein complex predictions from hierarchical clustering of elution profiles as shown in Fig 2.

Supp Fig 1

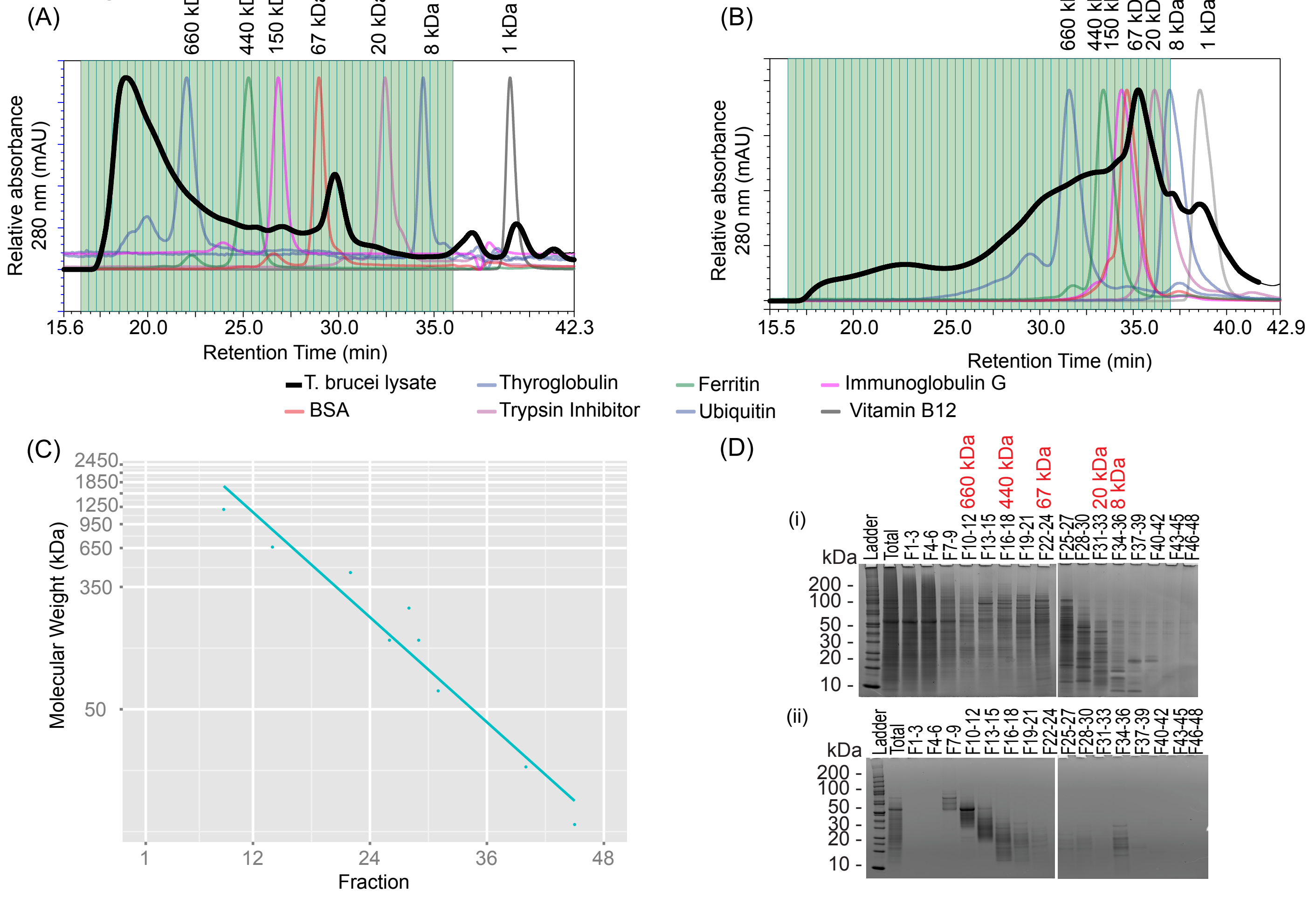
(A)
660 kDa
440 kDa
150 kDa
67 kDa
20 kDa
8 kDa
1 kDa
Relative absorbance 280 nm (mAU)
15.6
20.0
25.0
30.0
35.0
42.3
Retention Time (min)
(B)
660 kDa
440 kDa
150 kDa
67 kDa
20 kDa
8 kDa
1 kDa
Relative absorbance 280 nm (mAU)
15.5
20.0
25.0
30.0
35.0
40.0
42.9
Retention Time (min)
T. brucei lysate
Thyroglobulin
Ferritin
Immunoglobulin G
BSA
Trypsin Inhibitor
Ubiquitin
Vitamin B12
(C)
2450
1850
1250
950
650
350
50
Molecular Weight (kDa)
1
12
24
36
48
Fraction
(D)
660 kDa
440 kDa
67 kDa
20 kDa
8 kDa
(i)
Ladder
Total
F1-3
F4-6
F7-9
F10-12
F13-15
F16-18
F19-21
F22-24
F25-27
F28-30
F31-33
F34-36
F37-39
F40-42
F43-45
F46-48
kDa
200
100
50
30
20
10
(ii)
Ladder
Total
F1-3
F4-6
F7-9
F10-12
F13-15
F16-18
F19-21
F22-24
F25-27
F28-30
F31-33
F34-36
F37-39
F40-42
F43-45
F46-48
kDa
200
100
50
30
20
10

Supp Fig 2

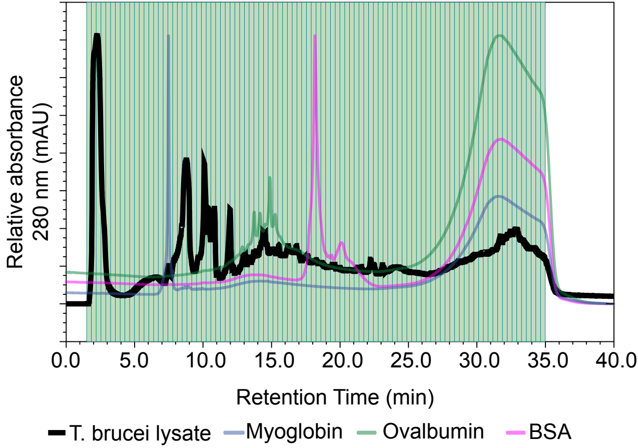

Relative absorbance
280 nm (mAU)
0.0
5.0
10.0
15.0
20.0
25.0
30.0
35.0
40.0
Retention Time (min)
T. brucei lysate
Myoglobin
Ovalbumin
BSA

Supp Fig 3

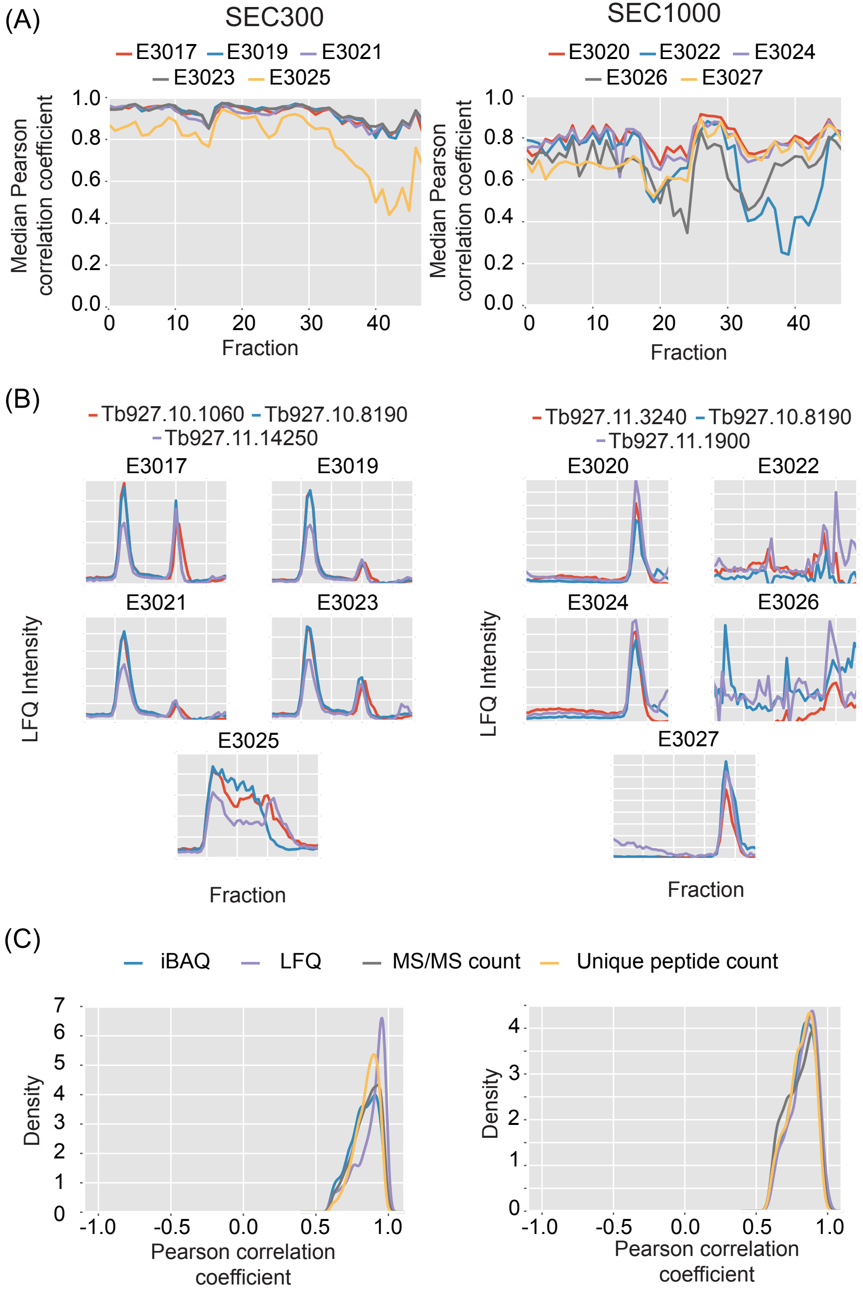
(A)
SEC300
E3017
E3019
E3021
E3023
E3025
Median Pearson correlation coefficient
1.0
0.8
0.6
0.4
0.2
0.0
0
10
20
30
40
Fraction
SEC1000
E3020
E3022
E3024
E3026
E3027
Median Pearson correlation coefficient
Fraction
(B)
Tb927.10.1060
Tb927.10.8190
Tb927.11.14250
E3017
E3019
E3021
E3023
E3025
LFQ Intensity
Fraction
Tb927.11.3240
Tb927.10.8190
Tb927.11.1900
E3020
E3022
E3024
E3026
E3027
LFQ Intensity
Fraction
(C)
iBAQ
LFQ
MS/MS count
Unique peptide count
Density
7
6
5
4
3
2
1
0
-1.0
-0.5
0.0
0.5
1.0
Pearson correlation coefficient
Density
4
3
2
1
0
Pearson correlation coefficient

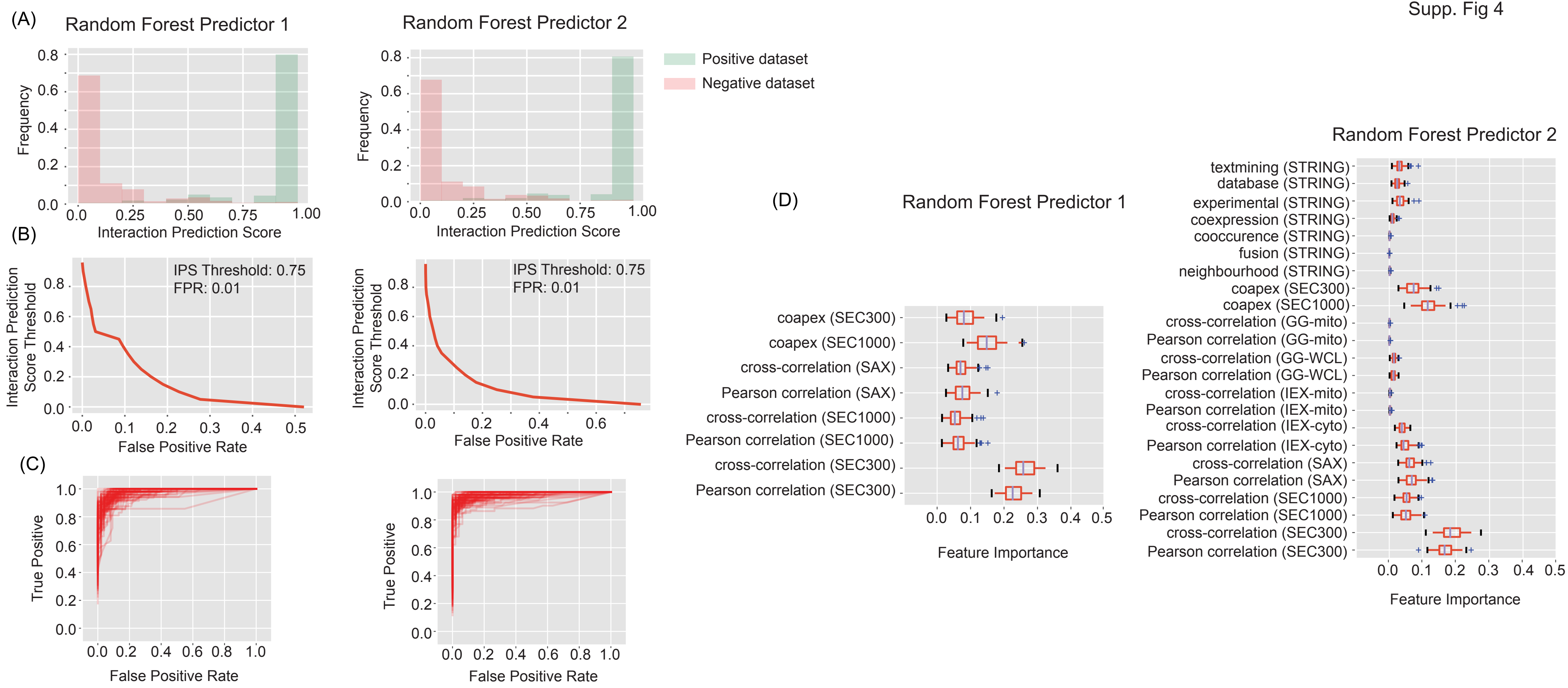
(A)
Random Forest Predictor 1
Random Forest Predictor 2
Positive dataset
Negative dataset
Frequency
Interaction Prediction Score
(B)
IPS Threshold: 0.75
FPR: 0.01
Interaction Prediction Score Threshold
False Positive Rate
(C)
True Positive
False Positive Rate
(D)
Random Forest Predictor 1
coapex (SEC300)
coapex (SEC1000)
cross-correlation (SAX)
Pearson correlation (SAX)
cross-correlation (SEC1000)
Pearson correlation (SEC1000)
cross-correlation (SEC300)
Pearson correlation (SEC300)
Feature Importance
Random Forest Predictor 2
textmining (STRING)
database (STRING)
experimental (STRING)
coexpression (STRING)
cooccurence (STRING)
fusion (STRING)
neighbourhood (STRING)
coapex (SEC300)
coapex (SEC1000)
cross-correlation (GG-mito)
Pearson correlation (GG-mito)
cross-correlation (GG-WCL)
Pearson correlation (GG-WCL)
cross-correlation (IEX-mito)
Pearson correlation (IEX-mito)
cross-correlation (IEX-cyto)
Pearson correlation (IEX-cyto)
cross-correlation (SAX)
Pearson correlation (SAX)
cross-correlation (SEC1000)
Pearson correlation (SEC1000)
cross-correlation (SEC300)
Pearson correlation (SEC300)
Feature Importance

Supp. Fig 5

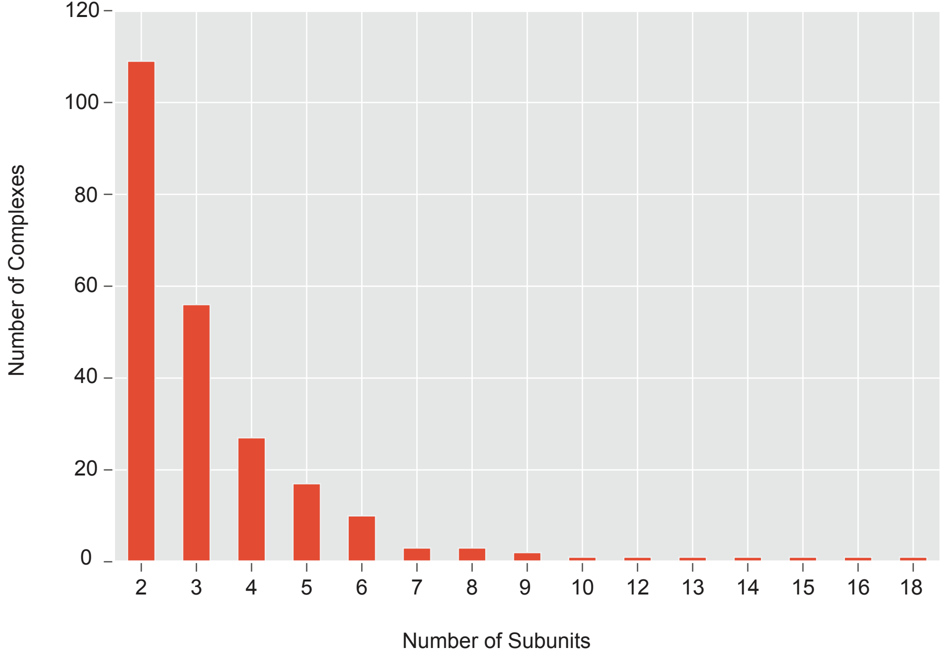
120
100
80
60
40
20
0
Number of Complexes
2
3
4
5
6
7
8
9
10
12
13
14
15
16
18
Number of Subunits

Supp Fig 6

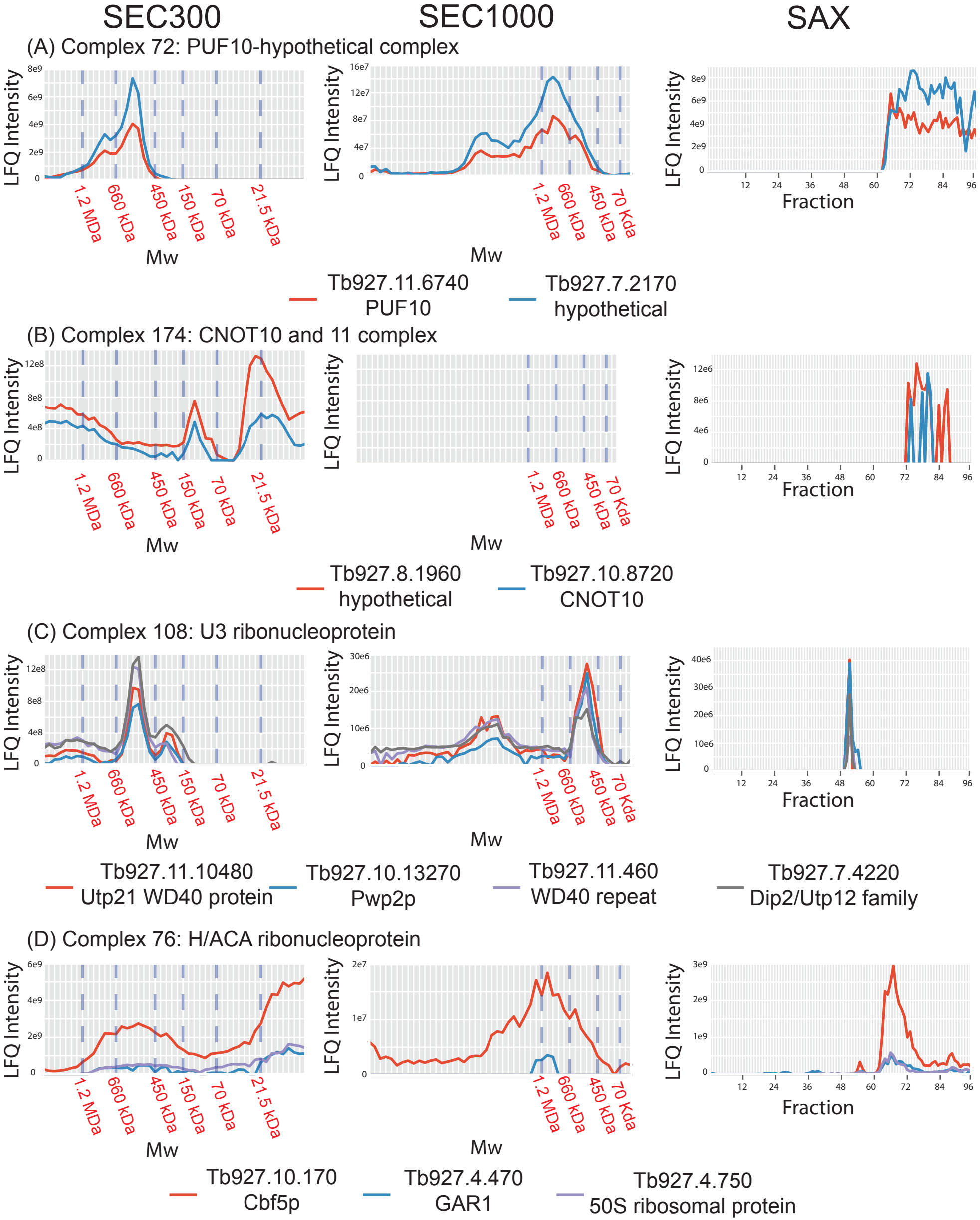
SEC300
SEC1000
SAX
(A) Complex 72: PUF10-hypothetical complex
LFQ Intensity
Mw
Fraction
1.2 MDa
660 kDa
450 kDa
150 kDa
70 kDa
21.5 kDa
70 Kda
Tb927.11.6740
PUF10
Tb927.7.2170
hypothetical
(B) Complex 174: CNOT10 and 11 complex
Tb927.8.1960
hypothetical
Tb927.10.8720
CNOT10
(C) Complex 108: U3 ribonucleoprotein
Tb927.11.10480
Utp21 WD40 protein
Tb927.10.13270
Pwp2p
Tb927.11.460
WD40 repeat
Tb927.7.4220
Dip2/Utp12 family
(D) Complex 76: H/ACA ribonucleoprotein
Tb927.10.170
Cbf5p
Tb927.4.470
GAR1
Tb927.4.750
50S ribosomal protein

Supp Fig 7

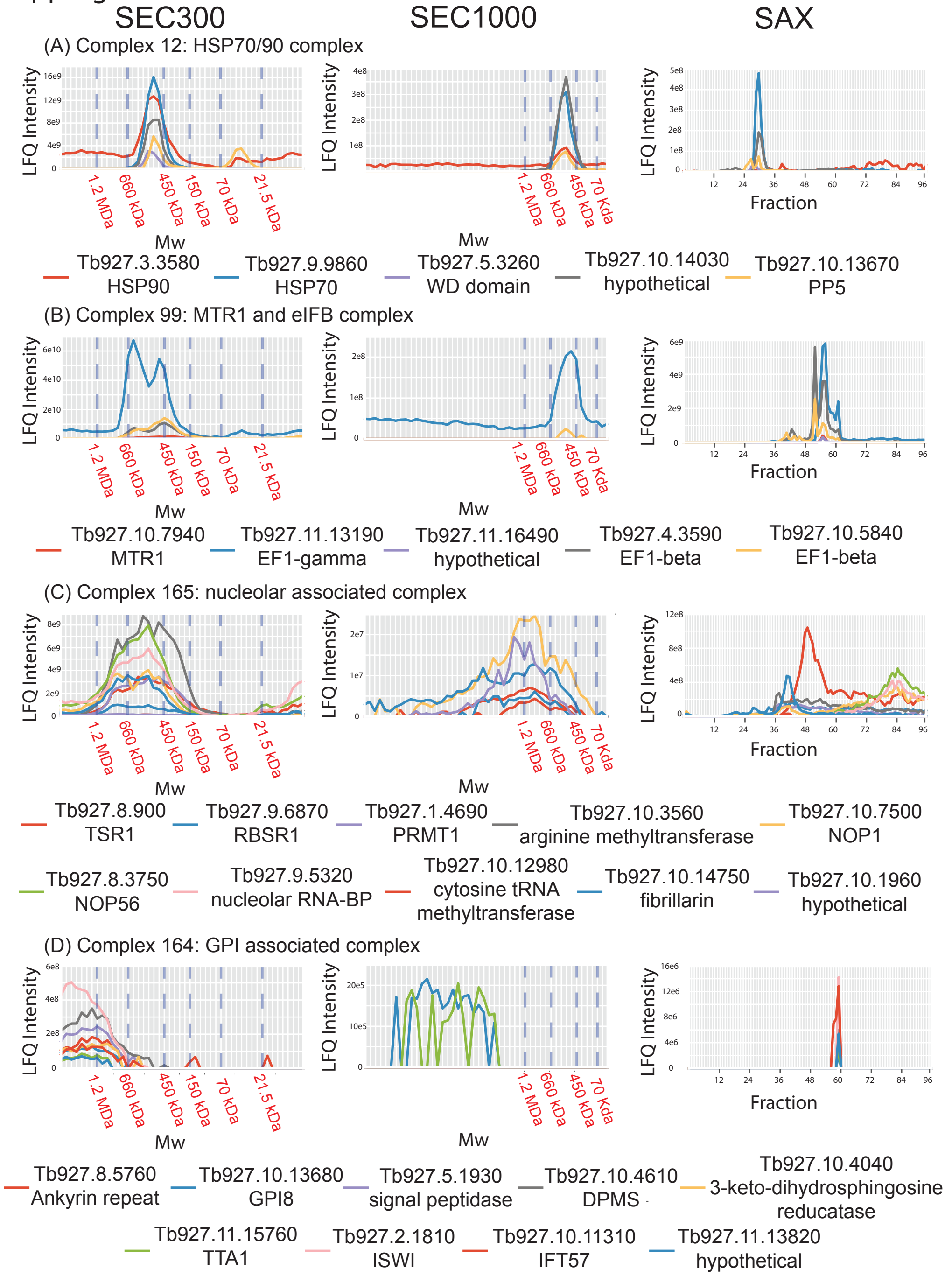
SEC300
SEC1000
SAX
(A) Complex 12: HSP70/90 complex
LFQ Intensity
Mw
Fraction
1.2 MDa
660 kDa
450 kDa
150 kDa
70 kDa
21.5 kDa
70 Kda
Tb927.3.3580 HSP90
Tb927.9.9860 HSP70
Tb927.5.3260 WD domain
Tb927.10.14030 hypothetical
Tb927.10.13670 PP5
(B) Complex 99: MTR1 and eIFB complex
Tb927.10.7940 MTR1
Tb927.11.13190 EF1-gamma
Tb927.11.16490 hypothetical
Tb927.4.3590 EF1-beta
Tb927.10.5840 EF1-beta
(C) Complex 165: nucleolar associated complex
Tb927.8.900 TSR1
Tb927.9.6870 RBSR1
Tb927.1.4690 PRMT1
Tb927.10.3560 arginine methyltransferase
Tb927.10.7500 NOP1
Tb927.8.3750 NOP56
Tb927.9.5320 nucleolar RNA-BP
Tb927.10.12980 cytosine tRNA methyltransferase
Tb927.10.14750 fibrillarin
Tb927.10.1960 hypothetical
(D) Complex 164: GPI associated complex
Tb927.8.5760 Ankyrin repeat
Tb927.10.13680 GPI8
Tb927.5.1930 signal peptidase
Tb927.10.4610 DPMS
Tb927.10.4040 3-keto-dihydrosphingosine reducatase
Tb927.11.15760 TTA1
Tb927.2.1810 ISWI
Tb927.10.11310 IFT57
Tb927.11.13820 hypothetical

Supp. Fig 8

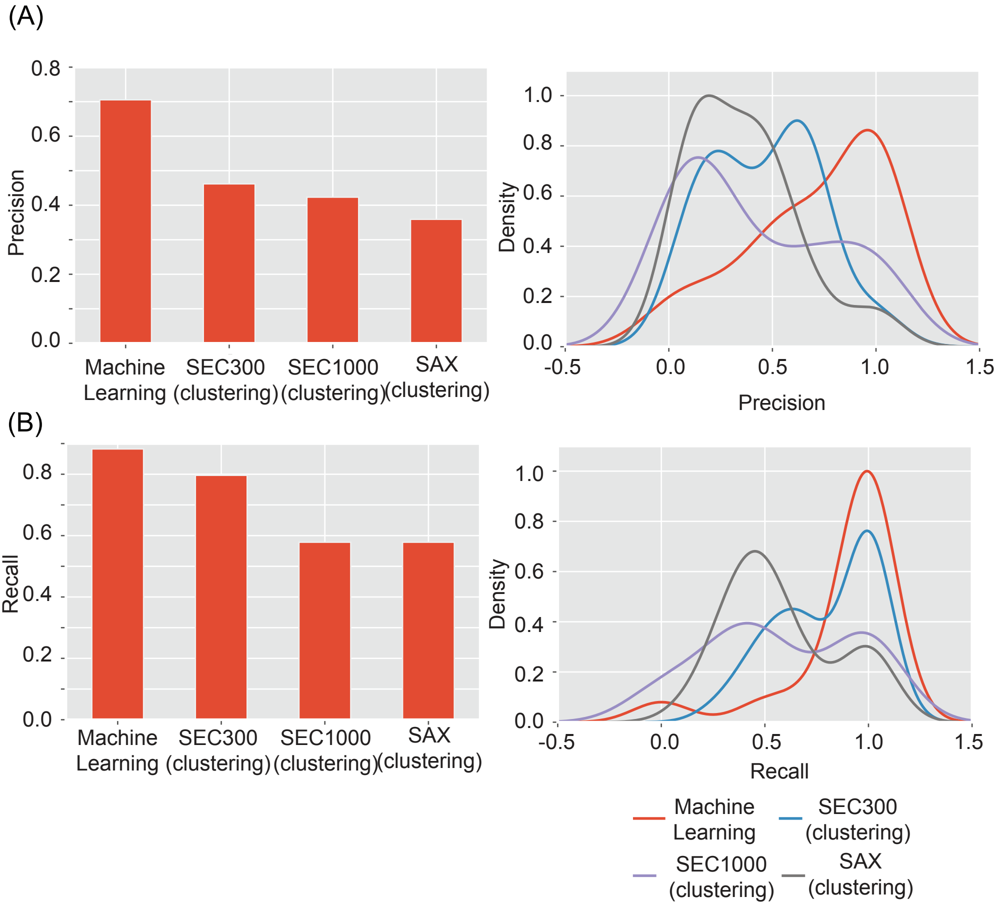
(A)
Precision
0.0
0.2
0.4
0.6
0.8
Machine Learning
SEC300 (clustering)
SEC1000 (clustering)
SAX (clustering)
Density
0.0
0.2
0.4
0.6
0.8
1.0
-0.5
0.0
0.5
1.0
1.5
Precision
(B)
Recall
0.0
0.2
0.4
0.6
0.8
Machine Learning
SEC300 (clustering)
SEC1000 (clustering)
SAX (clustering)
Density
0.0
0.2
0.4
0.6
0.8
1.0
-0.5
0.0
0.5
1.0
1.5
Recall
Machine Learning
SEC300 (clustering)
SEC1000 (clustering)
SAX (clustering)

Supp. Fig 9

(A)

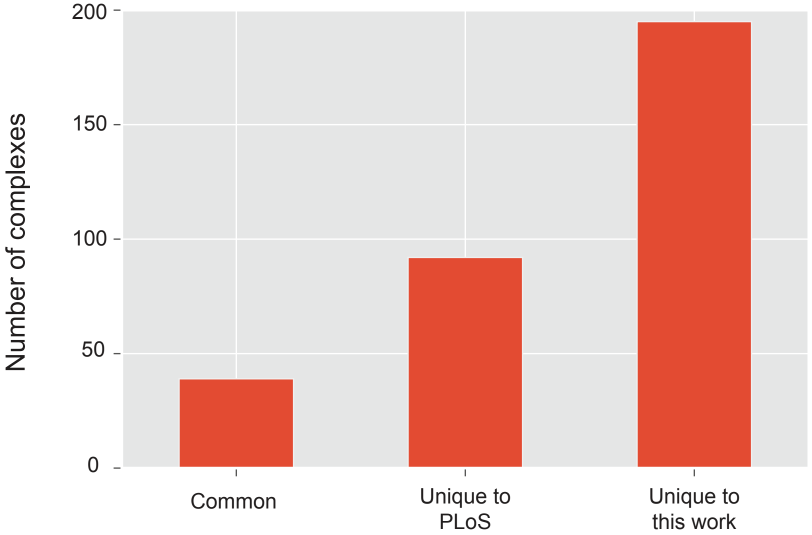


Mean Pearson Correlation Coefficient

| | Common | Unique to PLoS | Unique to this work |
|---|---|---|---|
| SEC300 | 0.89 | 0.24 | 0.91 |
| SEC1000 | 0.89 | 0.37 | 0.83 |
| SAX | 0.58 | 0.09 | 0.57 |
| IEX-cyto | 0.56 | 0.7 | 0.51 |
| IEX-mito | 0.36 | 0.67 | 0.55 |
| GG-WCL | 0.84 | 0.56 | 0.46 |
| GG-mito | 0.8 | 0.41 | 0.61 |

(B)

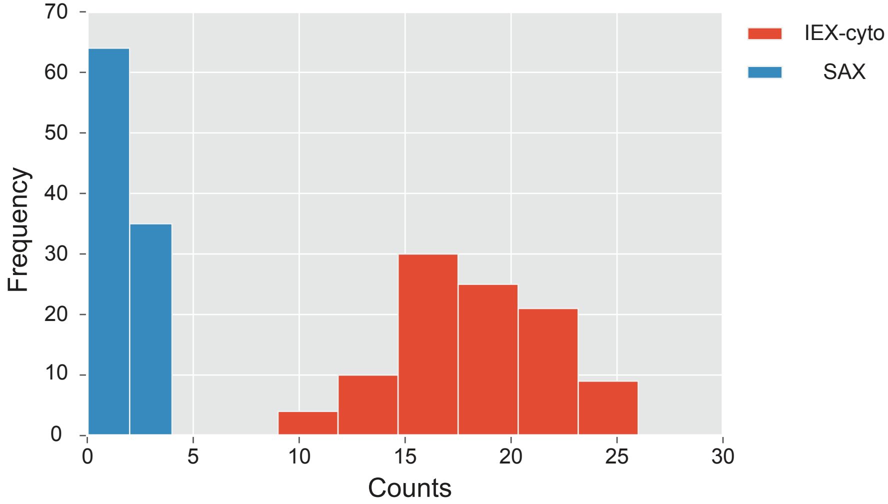

Supp Fig 10

search for user defined list of proteins

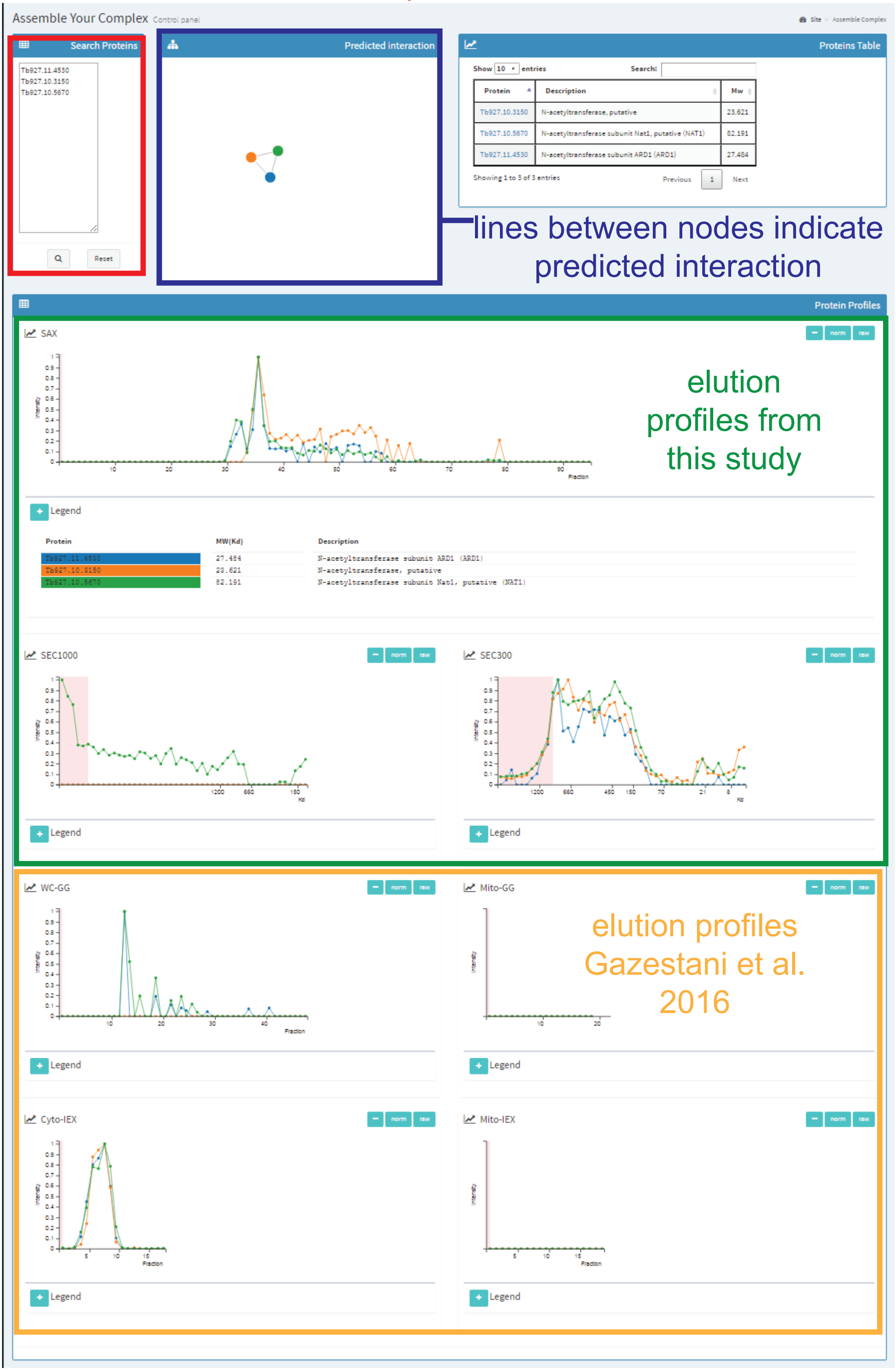

Supp Fig 11

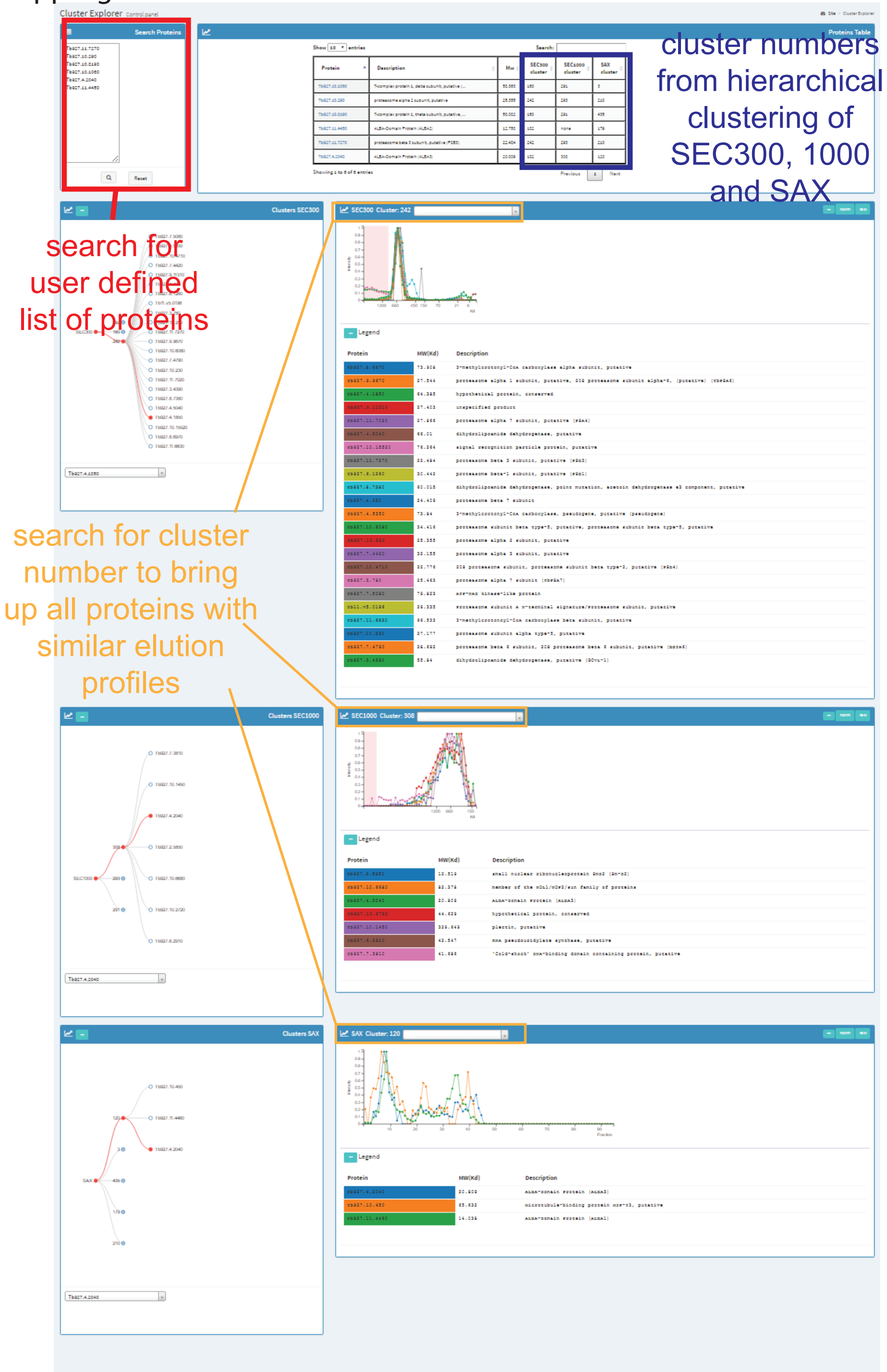
Cluster Explorer
Search Proteins
Proteins Table
cluster numbers from hierarchical clustering of SEC300, 1000 and SAX
search for user defined list of proteins
Clusters SEC300
SEC300 Cluster: 242
search for cluster number to bring up all proteins with similar elution profiles
Clusters SEC1000
SEC1000 Cluster: 308
Clusters SAX
SAX Cluster: 120
Legend
